# Comparative Genomic Analysis of Two *Vibrio harveyi* Strains from *Larimichthys crocea* with Divergent Virulence Profiles

**DOI:** 10.3390/microorganisms13051129

**Published:** 2025-05-14

**Authors:** Kequan Wang, Chaozheng Zhang, Hetron Mweemba Munang’andu, Cheng Xu, Wenlong Cai, Xiaojun Yan, Zhen Tao

**Affiliations:** 1 School of Fisheries, Zhejiang Ocean University, Zhoushan 316022, China; wangkequan@zjou.edu.cn (K.W.); 2022180@zjou.edu.cn (C.Z.); yanxj@zjou.edu.cn (X.Y.); 2 Faculty of Biosciences and Aquaculture, Nord University, 8049 Bodø, Norway; hetron.m.munangandu@nord.no; 3 Department of Paraclinical Sciences, Faculty of Veterinary Medicine, Norwegian University of Life Sciences, 1433 Ås, Norway; cheng.xu@nmbu.no; 4 Department of Infectious Diseases and Public Health, Jockey Club College of Veterinary Medicine and Life Sciences, City University of Hong Kong, Hong Kong, China; wenlocai@cityu.edu.hk

**Keywords:** *Vibrio harveyi*, comparative genomics, virulence factors, mobile genetic elements, plasmid transfer

## Abstract

*Vibrio harveyi* is a significant pathogen in marine aquaculture, causing vibriosis in various marine species. This study presents a comparative genomic analysis of two *V. harveyi* strains, N8T11 and 45T2, which exhibit differing virulence profiles. Virulence assays revealed that N8T11 caused 92% mortality in infected fish, while 45T2 resulted in 0% mortality. Whole-genome sequencing revealed that strain N8T11 harbors five plasmids (pN8T11a, pN8T11b, pN8T11c, pN8T11d and pN8T11e) absent in 45T2, encoding genes potentially linked to virulence, such as siderophore-mediated iron acquisition and stress response mechanisms. Pan-genome analysis highlighted substantial genomic plasticity within *V. harveyi*, with mobile genetic elements, including plasmids and prophages, contributing to horizontal gene transfer. Conjugation experiments demonstrated that all five N8T11 plasmids can transfer to 45T2 with efficiencies up to 87%, with pN8T11b remaining stable across multiple subcultures, enabling the dissemination of virulence-associated genes. These findings suggest that plasmid-mediated gene transfer plays a role in the virulence variability observed between *V. harveyi* strains. This study contributes to understanding the genomic factors underlying pathogenicity in *V. harveyi* and provides insights for future research aimed at controlling vibriosis in aquaculture.

## 1. Introduction

*Vibrio harveyi* is a major pathogenic bacterium in marine aquaculture, affecting a wide range of species, including fish, shrimp, and mollusks [1,2,3]. This pathogen causes vibriosis, characterized by hemorrhagic septicemia, skin lesions, tissue necrosis, and significant mortality in aquaculture systems [1]. Research suggests vibriosis could contribute to half of the economic losses in Asian marine fish aquaculture [4]. The economic burden of vibriosis on global aquaculture underscores the urgent need to better understand the genomic factors that drive *V. harveyi* pathogenicity and its environmental adaptability.

The pathogenicity of *V. harveyi* is mediated by a diverse array of virulence factors, including extracellular toxins, hemolysins, proteases, and quorum-sensing systems, which collectively enable host colonization, immune evasion, and environmental adaptation [5,6]. The genomic plasticity of this bacterium plays a critical role in its ability to acquire new genetic traits that enhance its survival and virulence. This adaptability is largely driven by mobile genetic elements (MGEs), such as plasmids, transposons, and prophage, which facilitate horizontal gene transfer (HGT) [7,8,9]. MGEs contribute not only to genetic diversity within *V. harveyi* populations but also facilitate the rapid spread of genes associated with virulence, antibiotic resistance, and environmental stress response [10,11]. Among these elements, plasmids have been identified as significant contributors to *V. harveyi* virulence [11]. However, the precise genomic differences, particularly in plasmid content, that distinguish highly pathogenic *V. harveyi* strains from non-pathogenic ones are not well understood and require further investigation.

Comparative genomic analysis has proven to be a powerful approach for identifying the molecular determinants of bacterial pathogenicity and environmental adaptability [12,13]. Advances in whole-genome sequencing technologies, phylogenetic reconstruction methods, and systematic functional annotations have enabled researchers to pinpoint critical genomic elements that distinguish pathogenic strains from non-pathogenic ones, such as MGEs and horizontally acquired virulence factors [13,14,15]. Horizontal gene transfer (HGT), mediated by mobile genetic elements, particularly plasmids, constitutes a critical evolutionary mechanism driving bacterial pathogenicity. In Vibrionaceae, plasmid-mediated HGT is a key evolutionary mechanism driving pathogenicity, with well-documented examples including the biotype II plasmid in Vibrio vulnificus and the pVA1 plasmid in *V. parahaemolyticus*, which enhance virulence through toxin production and host invasion [16,17,18,19].

This study builds on prior research involving *V. harveyi* strains isolated from large yellow croaker (LYC; *Larimichthys crocea*) with contrasting virulence profiles: the highly virulent N8T11 strain, which carries multiple plasmids, and the plasmid-deficient, less pathogenic 45T2 strain [20]. We investigate whether these plasmids encode traits and facilitate their dissemination via HGT. The genomic divergence between N8T11 and 45T2 makes them an ideal model for studying the role of plasmid-driven HGT in bacterial virulence. Using integrated approaches, including whole-genome sequencing, phylogenetic analysis, plasmid transfer experiments, and functional annotation, this study identifies the genetic factors underlying their pathogenicity differences. These findings advance our understanding of the genomic basis of *V. harveyi* virulence and provide a foundation for developing targeted interventions to control vibriosis in aquaculture systems.

## 2. Materials and Methods

### 2.1. Bacterial Strains and Culture Conditions

Two *V. harveyi* strains, N8T11 and 45T2, were used in this study. Strains N8T11 and 45T2 were isolated using standardized protocols optimized from our laboratory’s screening of 129 presumptive Vibrio isolates from aquaculture systems in Zhejiang and Fujian provinces. Tissue samples (liver, spleen, kidney, and ulcerative lesions) from moribund fish were aseptically collected with inoculation loops and streaked onto thiosulfate-citrate-bile salts-sucrose (TCBS, Hopebio Co., Ltd., Qingdao, China) selective agar using a three-zone quadrant method. Following 24 h incubation at 28 °C, dominant yellow-pigmented colonies were purified through three successive streak plates on TCBS to ensure monoculture. Strains were cryopreserved at −80 °C in our laboratory’s glycerol-based preservation system (20% *v*/*v* glycerol in tryptic soy broth) [20].

The highly virulent N8T11 strain was isolated in October 2019 from LYC liver at a fish farm in Zhoushan, Zhejiang Province, China, while the less-pathogenic 45T2 strain was isolated in August 2015 from LYC kidney in Ningbo, Zhejiang Province, China. Through plasmid extraction and gel electrophoresis, it was preliminarily determined that strain 45T2 does not harbor any plasmids, whereas strain N8T11 contains at least four plasmids.

Both strains were cultured on Tryptic Soy Agar (TSA, Hopebio Co., Ltd., Qingdao, China) or in Tryptic Soy Broth (TSB, Hopebio Co., Ltd., Qingdao, China) supplemented with 1% NaCl (Sinopharm Chemical Reagent Co., Ltd., Shanghai, China) (TSA-1 or TSB-1) at 28 °C to mimic marine conditions. For plasmid propagation, Escherichia coli strains were grown in Luria-Bertani (LB, Hopebio Co., Ltd., Qingdao, China) medium with appropriate antibiotics at 37 °C. The *E. coli* DH5α strain was commercially obtained from Vazyme Biotech Co., Ltd. (Nanjing, China). Details of *E. coli* strains and plasmid tools are provided in Table 1.

### 2.2. Virulence Assays

Virulence of *V. harveyi* strains N8T11 and 45T2 were verified in one-year-old LYC, based on previous findings indicating N8T11 as highly virulent and 45T2 as less-pathogenic [20]. Strains were cultured in TSB-1 at 28 °C for 18 h, centrifuged at 5000× *g* for 10 min, resuspended in PBS, and adjusted to 10^6^ CFU/mL. Fish (n = 30 per group; mean weight 112.4 ± 8.9 g) were lightly anesthetized with 100 mg/L tricaine methane sulfonate (MS-222) in aerated water and intramuscularly injected with 100 μL of bacterial suspension (10^6^ CFU/fish) or 100 μL PBS as a negative control. Post-injection, fish were maintained in aerated tanks at 28 ± 2 °C with daily water exchanges and fed a commercial diet (Fuzhou Haida Feed Co., Ltd., Fuzhou, China) from day 3 onward. Mortality was recorded daily over 7 days, and necropsies were performed on deceased fish to recover *V. harveyi* from spleen samples. The experiment was repeated twice. Surviving fish or those reaching humane endpoints were euthanized with 400 mg/L MS-222, with euthanasia confirmed by cessation of opercular movement and absence of response to stimuli. Survival differences were analyzed using a log-rank test. All procedures complied with national guidelines for laboratory animal care and were approved by the Ethics Committee of Zhejiang Ocean University (Approval no. ZJOU 2024146).

### 2.3. Genomic DNA Extraction

Genomic DNA was extracted from *V. harveyi* strains N8T11 and 45T2 using the Wizard^®^ Genomic DNA Purification Kit (Promega, Madison, WI, USA) as per the manufacturer’s protocol. The quantity and purity of the DNA were assessed using a TBS-380 fluorometer (Turner BioSystems, Sunnyvale, CA, USA) and NanoDrop 2000 spectrophotometer (Thermo Fisher Scientific, Waltham, MA, USA). Only samples with an OD^260/280^ ratio of 1.8–2.0 and concentrations above 50 ng/μL (total ≥ 20 μg) were used for sequencing. The extracted DNA was stored at −20 °C. DNA integrity was assessed using agarose gel electrophoresis to confirm the presence of high-molecular-weight DNA without degradation.

### 2.4. Whole-Genome Sequencing

A hybrid sequencing approach, combining short-read Illumina sequencing and long-read Nanopore technology, was utilized, with sequencing services provided by Majorbio (Shanghai, China). For Illumina Sequencing, genomic DNA (1 μg per sample) was fragmented into 400–500 bp fragments using a Covaris M220 Focused Acoustic Shearer (Covaris, Woburn, MA, USA). Libraries were prepared with the NEXTflex™ Rapid DNA-Seq Kit (PerkinElmer, Waltham, MA, USA), involving end-repair, A-tailing, and adapter ligation, followed by PCR amplification. Sequencing was performed as 150 bp paired-end reads on an Illumina HiSeq X Ten [22]. For Nanopore sequencing, Nanopore libraries were prepared using the SQK-LSK109 ligation sequencing kit and multiplexed with the EXP-NBD104 barcoding kit (Oxford Nanopore Technologies, Oxford, UK). Sequencing was conducted on an R9.4.1 flow cell using a MinION device (Oxford Nanopore Technologies, Oxford, UK). Raw reads were basecalled and demultiplexed using Guppy base caller (version 3.1.5), obtained from the Oxford Nanopore Technologies community site.

### 2.5. Genome Assembly

A hybrid approach combining Nanopore long reads and Illumina short reads was employed for de novo genome assembly. Raw Illumina reads were quality-filtered using fastp (version 0.23.0) to remove low-quality bases and adapter sequences [23]. Nanopore reads were basecalled, demultiplexed, and trimmed with a minimum Q score threshold of 7. Cleaned short and long reads were subsequently assembled using Unicycler (version: 0.4.8) [24] to generate complete genome sequences. The final assembly was polished with Pilon (version: 1.22), which utilized short-read alignments to correct residual errors, enhancing the base-level accuracy of the assembly [25]. Raw sequence data were submitted to the National Center for Biotechnology Information (NCBI) Sequence Read Archive (SRA) under the BioProject IDs SAMN41105143 and SAMN41104981.

### 2.6. Genome Annotation

Genome annotation was performed using widely adopted tools to ensure comprehensive functional and structural analysis. Prokaryotic Genome Annotation Pipeline (PGAP version: 6.7) from NCBI [26] was used to predict coding sequences (CDS) and generate basic genomic annotations, which were subsequently uploaded to NCBI for validation. For further refinement, Prokka [27] was employed to predict open reading frames (ORFs), transfer RNAs (tRNAs), and ribosomal RNAs (rRNAs). To visualize the genomic structure, Proksee was used to generate circular genome maps and integrate Prokka’s output for a more intuitive presentation of the genomic data [28]. All gene designations are based on annotations from PGAP as performed by NCBI. Annotation results are summarized in Table 2. To enable comparative analysis of Clusters of Orthologous Groups (COGs) between the two strains, we utilized the EggNOG database (version 5.0) [29] with Diamond blastp (version 2.1.8) [30], applying stringent criteria (E-value threshold of ≤1 × 10^−5^ and a maximum of five blast hits per query). This multistep approach allowed us to systematically identify statistically significant differences in COG functional categories between the two genomes.

### 2.7. Identification and Analysis of Mobile Genetic Elements and CRISPR-Cas Systems

Plasmid sequences were identified using PlasFlow (version 1.1) [31], which helped distinguish plasmid-associated contigs from the whole-genome assembly. Contigs were filtered using a threshold of 0.7 to ensure the accuracy of plasmid predictions. The assembly was refined based on the predicted plasmid sizes and quantities, as outlined in previous work. To compare plasmid sequences, we used Mash screen searches against the PLSDB plasmid sequence database [32]. A maximum *p*-value of 0.1 and a maximum distance of 0.1 were used for plasmid comparison. Insertion sequences were identified using IS Finder, employing the BlastN algorithm with the following settings: E-value ≤ 1 × 10^−10^, a maximum of 30 blast hits, word size = 11, and gap penalties of 5 for existence and 2 for extension [33]. Genomic Islands (GIs) were predicted using Island Viewer (version: 4), which integrates multiple methods for the detection of horizontally acquired genomic regions [34]. Prophage regions were identified and analyzed using PHASTER, using default parameters optimized for sensitivity to identify complete and incomplete prophage regions within the genome [35]. CRISPR-Cas systems were detected using CRISPRCasFinder (version: 2022_04_14) [36] with the following prediction parameters: minRL = 23, maxRL = 50, Minimal Spacers size in function of Repeat size = 0.6, Maximal Spacers size in function of Repeat size = 2.5, Maximal allowed percentage of similarity between Spacers = 60%, Percentage mismatches allowed between Repeats = 20%, Percentage mismatches allowed for truncated Repeat = 33%. The Cas model was detected by typing and sub typing.

### 2.8. Phylogenetic Analysis and Average Nucleotide Identity (ANI)

To confirm the identification of the sequenced strains as *V. harveyi* and assess their species-level relatedness, we performed a genome-wide phylogenetic analysis. Genome sequences of 16 closely related Vibrio species were retrieved from the GenBank database. A phylogenetic tree was constructed using kSNP3.0 [37], following the guidelines provided in the kSNP 3.0 manual, with k-mer values calculated using the kchooser tool. To further explore the phylogenetic relationships of strains N8T11 and 45T2 with other *V. harveyi* strains from diverse hosts and geographical regions, we constructed an additional phylogenetic tree using the same kSNP3.0 approach. ANI values were calculated using the method described by Yoon et al. [38]. The ANI analysis included genomes from 12 closely-related *Vibrio* species, such as *V. parahaemolyticus* and *V. alginolyticus*, along with other publicly available genomes from NCBI. The ANI results provided a quantitative measure of the genomic similarity and relatedness among the strains at the species level. The available genomic information used for the phylogenetic comparison of strains N8T11 and 45T2 was sourced from the NCBI database. Detailed information about the bacterial strains used, including host, geographical location, and accession numbers, is available in Supplementary Table S1.

### 2.9. Genomic Collinearity Analysis Between V. harveyi Strains N8T11 and 45T2

Genomic collinearity between strains N8T11 and 45T2 was assessed using the TBtools-II software (version 2.056) [39], a versatile bioinformatics software for genomic data analysis. The integrated MCScanX module [40] was utilized to identify collinear regions based on BLAST (version 2.13.0) alignments, with parameters set to an E-value threshold of ≤1 × 10^−10^ and a maximum of five blast hits per query. Genome alignments were visualized using the Genome Ks Dot Plot module within TBtools, applying parameters of an E-value threshold of ≤1 × 10^−3^ and a maximum of ten blast hits per query.

### 2.10. Pan-Genome Analysis

The pan-genome analysis of 33 *V. harveyi* strains was performed using the Integrated Pan-genome Analysis (IPGA) web service [41], a tool designed for comprehensive analysis, comparison, and visualization of prokaryotic genomes. Predicted genes were grouped based on sequence similarity and categorized into four groups: Core genome (≥99% presence, present in all 33 strains), Soft core genome (99% > presence ≥ 95%, found in 32 strains), Shell genome (95% > presence ≥ 5%, found in 2–31 strains), and Private genome (5% > presence ≥ 0%, found in only one strain), following the classification scheme described by Sonnenberg et al. [42]. The clustering analysis was conducted with the following parameters: an identity threshold of 70%, a core gene ratio of 0.95, and a support threshold of −1. Additional genomic datasets for comparative analysis were sourced from publicly available sequences in the NCBI databases.

### 2.11. Identification of Virulence Factors, Resistance Genes, and Secreted Proteins

Virulence-associated genes were assessed using the Virulence Factor Database (VFDB) with BLAST (version 2.13.0) alignments set to an E-value threshold of ≤1 × 10^−5^ and a maximum of five blast hits per query [43,44]. Antibiotic resistance genes were predicted using the Comprehensive Antibiotic Resistance Database (CARD) [45]. DNA sequences were analyzed with strict hit criteria (≥95% similarity), and only strict hits were retained for further analysis. Genes encoding secretion system components were identified using Diamond (version 2.1.8) [30], with searches performed against the KEGG database [46]. The E-value threshold was set to ≤1 × 10^−5^, with a maximum of five blast hits per query. The prediction of signal peptides indicative of secreted proteins was carried out using SignalP (version: 4.1), applying Dmaxcut values of 0.51 for transmembrane proteins and 0.57 for non-transmembrane proteins, with default positional limits [47].

### 2.12. Plasmid Conjugation Assay

The transfer of plasmids from the pathogenic *V. harveyi* strain N8T11 to the non-pathogenic strain 45T2 was assessed using a standard bacterial conjugation assay. A kanamycin-resistant variant of strain 45T2 (45T2-KmR) was generated through the electroporation of the mini-Tn7T-Km plasmid, following the protocol established by Choi and Schweizer (2006) and adapted by Tao et al. (2024) [21,48]. The kanamycin resistance marker facilitated the selective identification of transconjugants on agar plates supplemented with kanamycin (100 µg/mL), ensuring the accurate detection of successful plasmid transfer events.

Conjugation was performed using a solid mating approach. In brief, overnight cultures of the donor strain N8T11 and the recipient strain 45T2-KmR were diluted into fresh TSB-1 medium and grown to the late exponential growth phase (approximately 3 × 10^9^ CFU/mL). The donor and recipient strains were mixed at a 1:1 ratio in a final volume of 200 µL. This mixture was placed onto a 0.22 µm filter membrane positioned on TSA-1 agar plates and incubated at 30 °C for either 24 or 48 h. After incubation, cells on the filter membrane were resuspended in 1 mL of PBS, and ten-fold serial dilutions were prepared. These dilutions were plated onto selective agar containing kanamycin and incubated at 30 °C for 24 h to allow colony formation. Successful plasmid acquisition by the kanamycin-resistant 45T2-KmR strain was confirmed via PCR using plasmid-specific primers. The primers were designed to target unique plasmid genes, enabling precise identification based on the whole-genome sequences of the strains. Additionally, primers targeting the *ompF* gene of 45T2 and the *moxR* gene of N8T11 were employed to differentiate between the donor and recipient strains. Conjugation efficiency was calculated as the proportion of PCR-confirmed transconjugants relative to the total number of recipient cells. Efficiency was assessed at two time points (24 h and 48 h post-conjugation) to evaluate the initial success and stability of plasmid transfer. Primer sequences are provided in Supplementary Table S2. All experiments were conducted in triplicate to ensure statistical reliability and reproducibility.

## 3. Results

### 3.1. Virulence Assessment in Large Yellow Croaker

**Figure 1:**
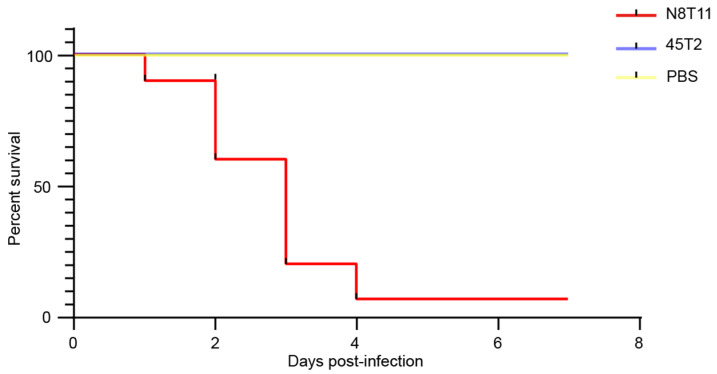
Comparative virulence assessment of *V. harveyi* strains N8T11 and 45T2 in large yellow croaker. Kaplan–Meier survival curves showing cumulative mortality over seven days following intramuscular infection with *V. harveyi* strains N8T11 (red line) and 45T2 (blue line). The higher mortality rate observed in fish infected with strain N8T11 highlights its elevated virulence compared to the less-virulent strain 45T2.

Two independent virulence experiments were conducted with *V. harveyi* strains N8T11 and 45T2 in 12-month-old LYC, producing consistent results. Data from one representative experiment are presented. In this experiment, intramuscular injection of 100 μL containing 10^6^ CFU of N8T11 resulted in a mortality rate of 93% (14/15 fish), whereas 45T2 caused no mortality (0%, 0/15 fish) over a 7-day observation period. Log-rank test analysis confirmed a significant difference in survival between groups (*p* < 0.001), as shown in Figure 1. Fish infected with N8T11 developed characteristic vibriosis symptoms, including hemorrhagic septicemia, ulcerative skin lesions, and lethargy, within 72 h post-infection. In contrast, fish injected with 45T2 or PBS exhibited no clinical symptoms during the same period. Necropsy of N8T11-infected fish confirmed the presence of *V. harveyi* in internal organs and ascite formation, whereas no *V. harveyi* was recovered from the tissues of 45T2-injected fish.

### 3.2. Genome Sequencing Quality Control and Coverage Analysis

The genomes of *V. harveyi* strains 45T2 and N8T11 were sequenced using Oxford Nanopore and Illumina platforms, yielding high-quality assemblies. For strain 45T2, the average sequencing depth was 198.93× (Nanopore) and 214.35× (Illumina), with coverages of 99.56% and 99.60%, respectively. For strain N8T11, the average depth was 172.18× (Nanopore) and 196.26× (Illumina), with coverages of 96.74% and 95.68%, respectively. The 45T2 assembly exhibited an N50 of 14,425 bp and an L50 of 22,448, indicating high contiguity, whereas the N8T11 assembly had an N50 of 12,720 bp and an L50 of 23,770. After quality control, Illumina sequencing for 45T2 produced 4,112,031 paired-end reads (1,245,240,150 clean bases), with Q20 and Q30 scores of 97.83% and 93.75%, respectively. For N8T11, Illumina sequencing generated 4,154,314 paired-end reads (1,258,102,217 clean bases), with Q20 and Q30 scores of 97.79% and 93.69%, respectively.

### 3.3. Genomic Features and Annotation of V. harveyi Strains 45T2 and N8T11

**Figure 2:**
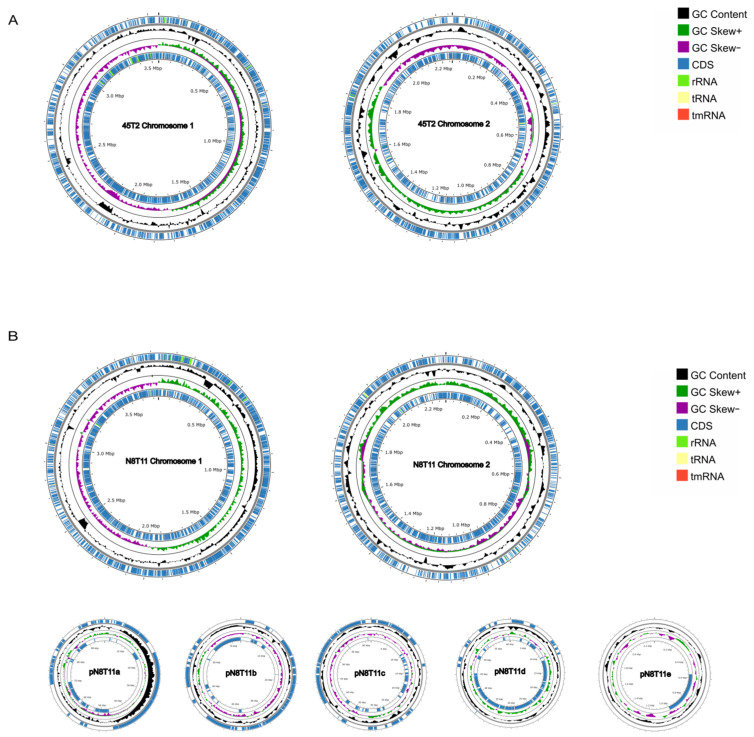
Circular genome plots of *V. harveyi* strains 45T2 and N8T11. (**A**) represents the circular genome of strain 45T2, while (**B**) illustrates the genome of strain N8T11, which includes five plasmids (pN8T11a–pN8T11e). From the outermost to the innermost rings: the first ring displays coding sequences (CDS), tRNA, rRNA, and mRNA on the positive strand; the second ring represents the genome size; the third ring illustrates GC content, where outward peaks indicate regions with higher-than-average GC content and inward peaks indicate lower-than-average GC content, with peak height reflecting the deviation from the mean; the fourth ring shows GC-skew, calculated as (G − C)/(G + C), where positive and negative values reflect strand asymmetry. The innermost ring displays CDS, tRNA, and rRNA on the negative strand.

The complete genome of *V. harveyi* strain 45T2 consists of two chromosomes. Chromosome 1 is 3,555,107 base pairs (bp) in length, while Chromosome 2 measures 2,254,156 bp, with a total of 5286 genes. In comparison, the genome of strain N8T11 also contains two chromosomes. Chromosome 1 is 3,842,609 bp long, and Chromosome 2 spans 2,274,153 bp. Additionally, N8T11 harbors five circular plasmids (pN8T11a, pN8T11b, pN8T11c, pN8T11d, and pN8T11e), which vary in size from 97,019 bp to 2285 bp. A total of 5943 genes have been annotated in the N8T11 genome. Detailed genomic data are provided in Table 2 and Figure 2.

**Figure 3:**
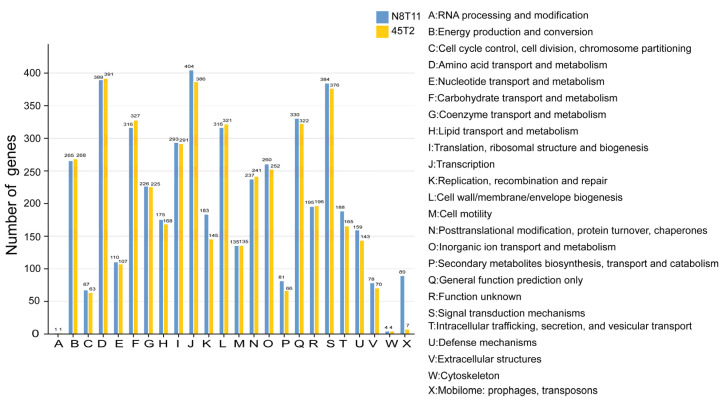
COG-based functional classification of genes in *V. harveyi* strains N8T11 and 45T2. The horizontal axis displays the COG functional categories, while the vertical axis indicates the number of genes assigned to each category. Each bar represents the distribution of genes across the COG categories for strains N8T11 and 45T2. A legend on the right provides descriptions of the functional categories, highlighting differences and similarities in gene function between the two strains.

Functional annotation using Clusters of Orthologous Groups (COG) revealed 4131 genes in 45T2, constituting 78.14% of the genome, and 4320 genes in N8T11, covering 72.69% of the genome. One key difference between the two strains lies in the mobilome, prophages, and transposons. Strain N8T11 contains over 90 genes in these categories, while strain 45T2 contains fewer than 20 (Figure 3).

### 3.4. ANI and Phylogenetic Analysis

**Figure 4:**
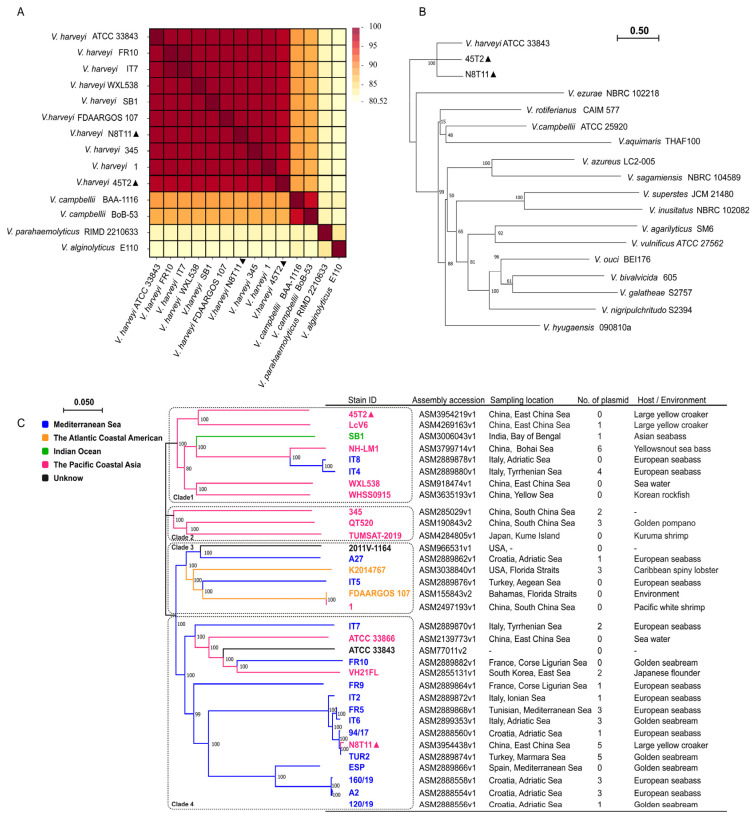
ANI heatmap and phylogenetic analysis of *V. harveyi* strains. The strains N8T11 and 45T2 sequenced in this study are marked with “▲” in the figures. (**A**) ANI heatmap showing pairwise similarity scores among 14 genomes, including *V. harveyi* strains N8T11 and 45T2, eight additional *V. harveyi* strains, two *V. campbellii* strains, one *V. parahaemolyticus* strain, and one *V. alginolyticus* strain. Higher ANI values indicate greater genomic similarity. (**B**) A maximum likelihood phylogenetic tree was constructed based on whole-genome concatenated alignments, with branch support assessed using 100 bootstrap replicates. The tree provides insights into the evolutionary relationships among the analyzed strains. (**C**) An intraspecies phylogenetic tree of *V. harveyi* illustrates five distinct color-coded clades representing geographic and genomic traits: Mediterranean (blue), Atlantic American (yellow), Indian Ocean (green), Pacific Asian (pink), and Unknown (black).

ANI and interspecies phylogenetic analysis confirmed the classification of strains N8T11 and 45T2 within the *V. harveyi* species. ANI analysis (Figure 4A) supported this classification, with ANI values surpassing the species-level threshold of 97%. Specifically, strain N8T11 exhibited 98.52% similarity to the reference strain SB1, while strain 45T2 demonstrated 97.72% similarity. In contrast, comparisons with non-*V. harveyi* species, such as *V. campbellii* and *V. alginolyticus*, yielded ANI values below 95%, indicating significant genetic divergence and affirming the species-level identification of both strains. The whole-genome phylogenetic tree (Figure 4B) illustrated that both strains clustered with the reference strain *V. harveyi* ATCC 33843, distinctly separating them from closely related *Vibrio* species.

Phylogenetic analysis of *V. harveyi* strains identified four distinct clades (Figure 4C), which showed limited correlation with geographic origin, host species, and environmental context. Strains originating from Asia, Europe, and North America were distributed across multiple clades, often overlapping. Strain N8T11, associated with large yellow croaker from the East China Sea, clustered with strain TUR2, which was isolated from golden seabream [49], within Clade 4 alongside Mediterranean strains linked to gilthead seabream and European seabass. Strain 45T2, also from the East China Sea, was positioned within Clade 1 along with other East Asian strains associated with large yellow croaker and yellow snout seabass. Strains from the South China Sea were predominantly found in Clade 2, primarily associated with aquaculture hosts such as golden pompano and kuruma shrimp. Clade 3 demonstrated intercontinental mixing, encompassing strains from Asia, Europe, and North America associated with diverse aquaculture species and environmental sources. While European strains, particularly those from the Mediterranean and Adriatic Seas, were mainly distributed in Clades 3 and 4, the presence of environmental and aquaculture-associated strains across all clades underscores the lack of strict clustering based on geography or host type.

### 3.5. Genomic Collinearity Between V. harveyi Strains N8T11 and 45T2

**Figure 5:**
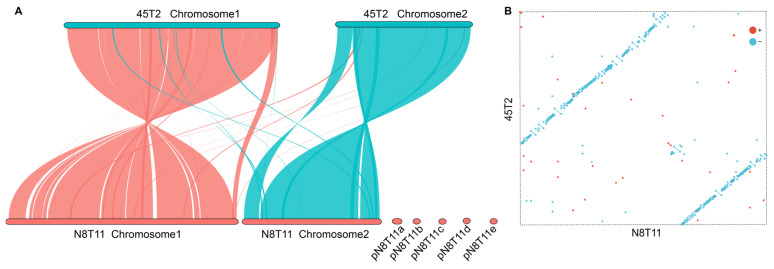
Comparative genomic collinearity of *V. harveyi* strains N8T11 and 45T2. (**A**) Collinearity analysis illustrating the conservation of gene order and sequence similarity between the two strains. Each axis represents the genomic sequence of one strain, with genes plotted based on their genomic positions. Lines connecting the axes indicate orthologous gene pairs, highlighting regions of high conservation and structural synteny. (**B**) Two-dimensional dot plot comparison of the genomes. The vertical axis represents the 45T2 genome, and the horizontal axis represents the N8T11 genome. Red dots indicate co-linear genomic fragments aligned in the same direction, while blue dots represent inverted fragments, reflecting structural rearrangements between the two strains.

Genomic collinearity analysis between strains 45T2 and N8T11 revealed substantial conservation at the chromosomal level. A total of 95.27% of the 45T2 genome aligned with the N8T11 genome, while 86.33% of the N8T11 genome aligned with 45T2 (Figure 5A). When plasmid sequences were excluded, the alignment percentage increased to 95.02%, suggesting that plasmid content plays a significant role in genomic divergence between the strains. The synteny dot plot (Figure 5B) further emphasized the alignment, with red dots indicating co-linear sequences in the same orientation, and blue dots representing localized inversions or reversed alignments.

### 3.6. Pan-Genome and Accessory Gene Variability in V. harveyi Strains

**Figure 6:**
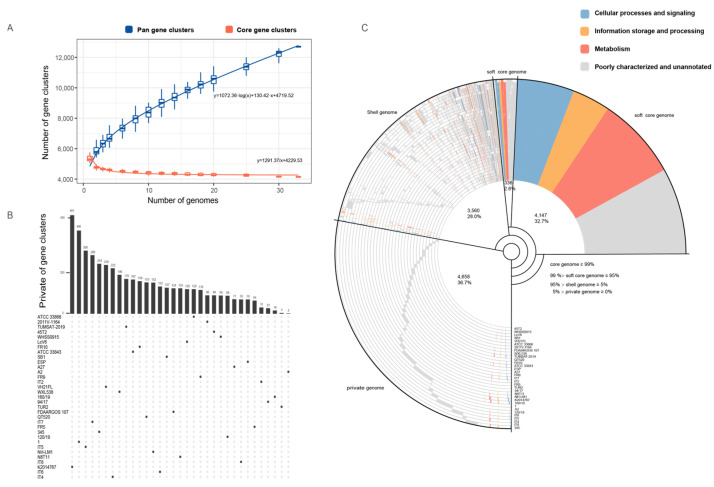
Pan-genome analysis of *V. harveyi*. (**A**) Pan-genome and core genome growth curves illustrating the relationship between the number of strains analyzed and gene cluster accumulation. The growth trends for core gene clusters (red line: y = 1291.37/x + 4229.53) and pan-gene clusters (blue line: y = 1072.36·log(x) + 130.42·x + 4719.52) were modeled based on data from 33 *V. harveyi* strains. (**B**) An Upset plot shows the distribution of unique and shared gene clusters across the analyzed strains, highlighting strain-specific and conserved gene sets. (**C**) COG annotation categorizes gene clusters into four groups: Core genome, Soft-core genome, Shell genome, and Private genome, as described in the text. Functional classifications are represented by blue for Cellular Processes and Signaling, yellow for Information Storage and Processing, red for Metabolism, and gray for poorly characterized or unannotated genes.

We obtained almost complete genome sequences of 31 *V. harveyi* strains (Supplementary Table S1). After including the two *V. harveyi* strains sequenced by our institute, a total of 33 *V. harveyi* strains were utilized for pan-genomic analysis. Pan-genome analysis of *V. harveyi* strains revealed an open pan-genome, with the number of gene clusters increasing as more genomes were added, reaching a total of 11,905 clusters (Figure 6A, blue curve). The core genome stabilized at 4147 clusters (Figure 6A, red curve), representing a conserved set of genes shared across all strains. Strains N8T11 and 45T2, both isolated from the East China Sea, contributed 124 and 90 unique genes, respectively (Figure 6B). Of the 124 private genes in N8T11, 55 were located on plasmids, accounting for approximately 44.4% of its strain-specific genomic content. The pan-genome composition (Figure 6C) revealed that the core genome comprised 32.7% of the total gene clusters, while the soft-core, shell, and private genomes accounted for 2.6%, 28.0%, and 36.7%, respectively.

### 3.7. Predicted Secretion Systems and Secreted Proteins

Comparative analysis of KEGG pathways revealed that both strains possess an identical repertoire of genes encoding secretion systems, including the Type II, Type III, and Type VI-P secretion systems, as well as components of the Sec-Srp and Tat pathways (Supplementary Table S3). To identify secreted proteins, the SignalP program was used, which predicted a significant difference in the number of unique secreted proteins between the two strains. Strain 45T2 was found to encode 21 unique secreted proteins, while strain N8T11 encoded 60 distinct secreted proteins, as summarized in Supplementary Table S4.

### 3.8. Antibiotic Resistance Genes and Virulence Factors

The CARD analysis identified five antibiotic resistance-related ORFs in strain N8T11 and six in strain 45T2, with substantial overlap between the two strains (Table 3). Shared resistance genes, including parE, vanT, CRP, adeF, and qacG, mediate resistance to multiple antibiotic classes, such as fluoroquinolones, glycopeptides, macrolides, tetracyclines, and disinfectants. These genes primarily function through mechanisms like target site modifications and efflux pump systems. Despite this difference, the two strains displayed comparable antibiotic susceptibility profiles, with all resistance genes localized on chromosomes.

The VFDB analysis revealed 147 virulence-related genes in strain N8T11 and 152 in strain 45T2, categorized into ten functional groups, including adhesion, immune evasion, and toxin production (Supplementary Table S5). While no significant differences were observed in their overall virulence gene profiles, further analysis revealed 35 unique virulence-related genes in strain N8T11 (Supplementary Table S6).

### 3.9. Genomic Islands, Prophages, Insertion Sequences, and CRISPR-Cas Systems

**Figure 7:**
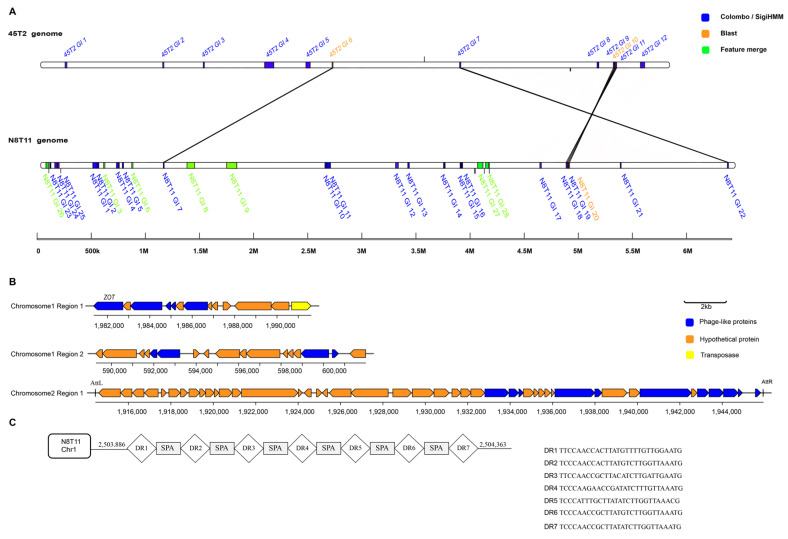
(**A**) Genomic islands detected in the genomes of N8T11 and 45T2, including several located on plasmids. Predictions were made using Colombo/SigiHMM (blue), BLAST (version 2.13.0; orange), and feature merging (green). (**B**) Prophage structures identified in the N8T11 genome are illustrated, highlighting intact prophages with detailed annotations of their open reading frames (ORFs) and phage attachment sites. (**C**) CRISPR-Cas systems identified in N8T11 are shown, displaying the genomic organization and arrangement of CRISPR arrays.

Comparative genomic analysis revealed substantial differences in GIs, IS elements, prophages, and CRISPR-Cas systems between *V. harveyi* strains N8T11 and 45T2. N8T11 contained 28 GIs, including six located on plasmids, while 45T2 had only 12 GIs, with five homologous pairs shared between the two strains (Figure 7A, Supplementary Table S7). N8T11 harbored a total of 1162 IS elements, distributed as 777 on chromosome 1, 292 on chromosome 2, and 93 on plasmids, whereas 45T2 contained only 13 IS elements across both chromosomes (Supplementary Table S8). Additionally, three intact prophages were identified in N8T11, including one located on chromosome 2 with multiple ORFs and phage attachment sites, while no prophages were detected in 45T2 (Figure 7B, Supplementary Table S9). Additionally, a credible CRISPR structure was identified exclusively in N8T11 (Figure 7C). However, no functional Cas enzymes were predicted in 45T2.

### 3.10. Plasmid Characterization in V. harveyi Strain N8T11

**Figure 8:**
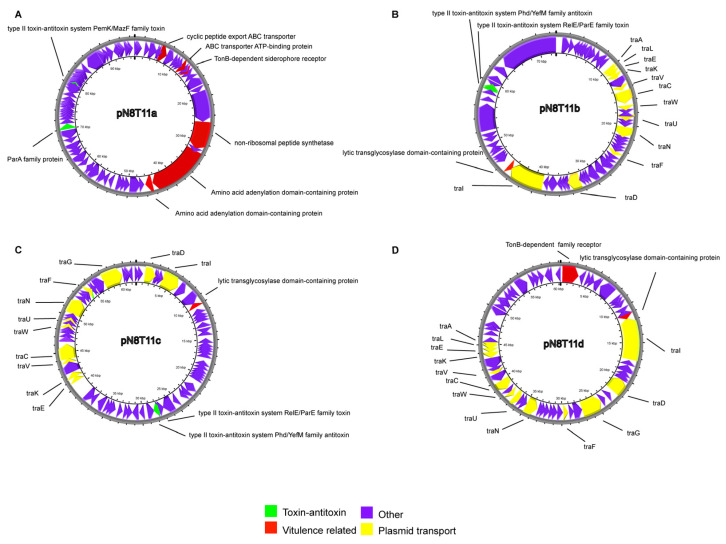
Annotation and functional characterization of *V. harveyi* N8T11 plasmids. Genomic maps of plasmids pN8T11a (**A**), pN8T11b (**B**), pN8T11c (**C**), and pN8T11d (**D**), highlighting key functional gene clusters. Yellow ORFs represent genes associated with F-type transport systems, red ORFs indicate potential strain-specific virulence genes, and green ORFs denote toxin-antitoxin system genes. Plasmid pN8T11e is not shown, as it contains only a single annotated ORF. Plasmid pN8T11e is not shown due to its single annotated ORF.

The plasmids of *V. harveyi* strain N8T11 were analyzed to identify genetic features and assess their evolutionary relationships. The annotated features of the five plasmids are detailed in Supplementary Table S10 and represented in Figure 8. Plasmid pN8T11a, the largest plasmid of the five, contains genes involved in siderophore-mediated iron acquisition, including a TonB-dependent receptor (AAIA71_28445), MbtH family non-ribosomal peptide synthetase proteins (AAIA71_28450), and TauD/TfdA dioxygenases (AAIA71_28455) (Figure 8A), along with a type II toxin-antitoxin system (PemK/MazF family, AAIA71_28460). Plasmid pN8T11b encoded an outer membrane protein OmpA (AAIA71_28740) (Figure 8B), while pN8T11c carried DNA repair-associated genes, including RadC (AAIA71_29120) and recombinase family proteins (AAIA71_29130) (Figure 8C). Plasmid pN8T11d contained an additional TonB-dependent receptor (AAIA71_29420) and multiple lytic transglycosylase domain-containing proteins (AAIA71_29425– AAIA71_29440) (Figure 8D). The smallest plasmid, pN8T11e, contained genes encoding a type III toxin-antitoxin system (AAIA71_29715). Plasmids pN8T11b, pN8T11c, and pN8T11d include components of the type IV secretion system (T4SS), indicating potential conjugative transfer capability.

Sequence comparisons with the PLSDB database, using Mash distance (maximum *p*-value 0.1, maximum distance 0.1), revealed evolutionary relationships (Supplementary Table S11). Plasmids pN8T11b, pN8T11c, and pN8T11d exhibited high similarity to plasmids from *V. harveyi*, *V. parahaemolyticus*, and *V. vulnificus*, whereas pN8T11a and pN8T11e showed no close matches. Specifically, pN8T11b was identical to *V. harveyi* QT520 plasmid p1 (Mash distance = 0.00) and similar to *V. harveyi* NH-LM1 plasmids Pvh4 (distance = 0.04) and Pvh5 (distance = 0.06). Plasmid pN8T11c matched *V. harveyi* QT520 plasmid p2 (distance = 0.00) and an unnamed *V. harveyi* VH21FL plasmid (distance = 0.06). Plasmid pN8T11d was most similar to *V. harveyi* NH-LM1 Pvh2 (distance = 0.06), followed by Pvh4 and Pvh5. Additionally, N8T11 plasmids shared genetic features with plasmids from *V. parahaemolyticus* (Fujian Province, China), and *V. vulnificus* (Spain), indicating broad phylogenetic distribution.

### 3.11. Conjugation and Stability of N8T11 Plasmids in 45T2

**Figure 9:**
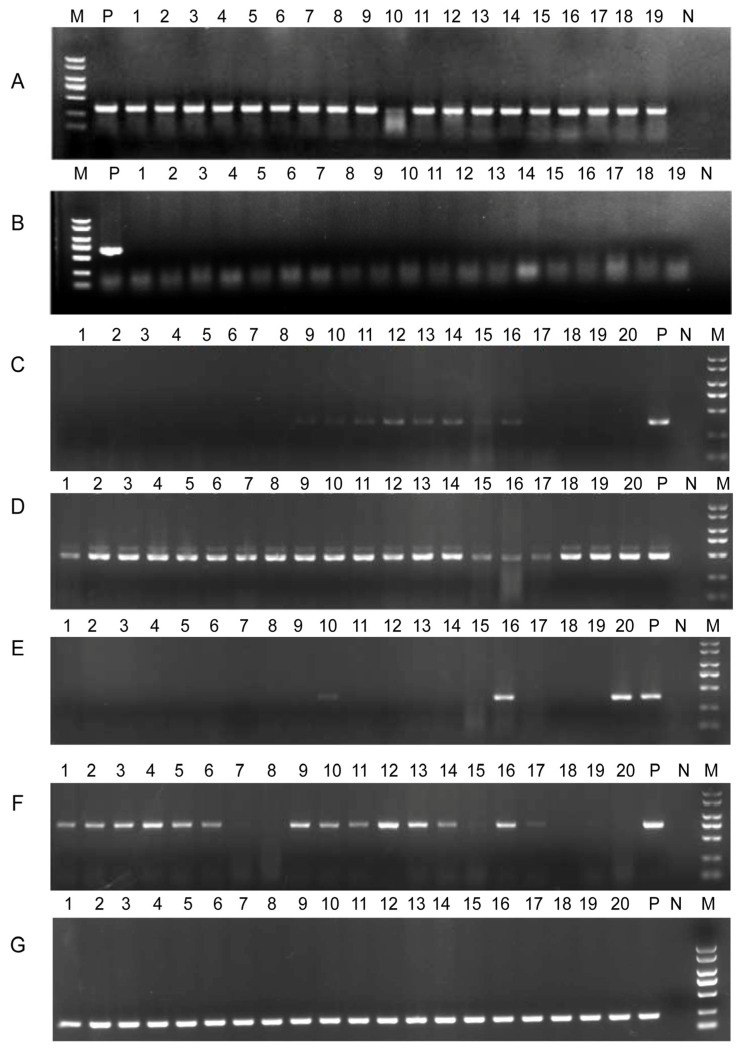
Validation of plasmid transfer from *V. harveyi* N8T11 to 45T2. (**A**,**B**) PCR validation of donor strain N8T11 and recipient strain 45T2 after antibiotic selection, confirming the presence of plasmid-specific target genes. (**C**–**G**) Specific PCR amplification patterns for individual plasmids pN8T11a, pN8T11b, pN8T11c, pN8T11d, and pN8T11e using plasmid-specific primers. Each panel displays distinct bands corresponding to plasmid-carried genes, verifying their presence and stability in the recipient strain 45T2. Lanes are labeled as M (DNA marker), P (positive control), and N (negative control).

We performed conjugation experiments between strains N8T11 and 45T2 to assess the transferability of plasmids carried by N8T11. The conjugation results showed variable plasmid transfer efficiencies. After 24 h, only plasmids pN8T11b and pN8T11e were successfully transferred, with efficiencies of 28/44 (63%) and 36/44 (82%), respectively. Extending the conjugation period to 48 h resulted in the transfer of all five plasmids, with the following efficiencies: pN8T11a (19/64; 29%), pN8T11b (56/64; 87%), pN8T11c (11/64; 17%), pN8T11d (49/64; 76%), and pN8T11e (55/64; 85%). After successful conjugation, the recipient strains were cultured, and only plasmid pN8T11b exhibited stable persistence in strain 45T2 across five subcultivation cycles, indicating its superior integration and stability. PCR validation of plasmid transfer and stability is shown in Figure 9.

## 4. Discussion

Comparative genomic analysis of *V. harveyi* strains N8T11 and 45T2 revealed significant differences in their virulence profiles, with N8T11 causing 93% mortality in LYC compared to 0% for 45T2. These disparities are primarily attributable to five plasmids (pN8T11a–e) in N8T11, which encode virulence-related factors, such as siderophore-mediated iron acquisition genes (e.g., TonB-dependent receptor, AAIA71_28445). Additionally, plasmids encode lytic transglycosylases (LTs) and toxin-antitoxin (TA) systems, which enhance bacterial fitness and plasmid stability, respectively, potentially supporting pathogenic persistence. Conjugation experiments confirmed that all five plasmids can transfer to 45T2, with pN8T11b exhibiting stable persistence across subcultures, underscoring HGT as a mechanism for disseminating virulence-associated traits.

The transfer of plasmid through conjugation has been shown to facilitate the acquisition of virulence traits. For instance, in *V. vulnificus* biotype II, a transferable plasmid has been shown to mediate virulence in eels, enabling the emergence of host-specific pathogenic clones through plasmid-mediated HGT [19]. Similarly, in *V. parahaemolyticus*, the acquisition of the pVA1 plasmid via natural transformation facilitates the production of toxins responsible for acute hepatopancreatic necrosis disease [17]. In this study, the transfer of N8T11 plasmids to 45T2, coupled with their sequence homology to plasmids from *V. harveyi*, *V. parahaemolyticus*, and *V. vulnificus*, indicates that plasmid exchange in aquaculture settings may amplify virulence. High-density farming likely exacerbates HGT by fostering bacterial interactions, increasing the risk of disease outbreaks [50].

GIs and prophages might also contribute to the pathogenic potential of the strain N8T11. In particular, these GIs harbor genes implicated in bacterial recognition, adhesion, and biofilm formation, including *VpadFA* and glycosyltransferases. Additionally, genomic islands encode proteins such as an AraC-family transcriptional activator, which regulates virulence gene expression, and phospholipase D family proteins, which might contribute to virulence by disrupting host cell membranes [51]. Prophages are known to facilitate the integration of toxin-encoding genes into bacterial genomes, as demonstrated by the ZOT enterotoxin located within an intact prophage region on *V. harveyi* strain N8T11 chromosome 1. This prophage shares structural similarities with PH1009 from *V. harveyi* strains isolated in the Philippines, which also contains the ZOT toxin, previously implicated in the strain’s virulence [52].

The distinct phylogenetic clustering of *V. harveyi* strains, such as N8T11 and 45T2, despite shared geographic origins, highlights the influence of environmental pressures, host adaptation, and human activities on the evolution and dissemination of this bacterium across diverse environments. The grouping of N8T11 with strains from diverse geographic origins, such as those from the Mediterranean region, indicates that *V. harveyi* strains can thrive in similar ecological niches across different environments. This is further supported by the intercontinental mixing observed in Clade 3, which includes strains from Asia, Europe, and North America associated with diverse aquaculture species and environmental sources. Such mixing likely results from human activities, such as aquaculture practices and global shipping activities, as well as natural water movements, which collectively facilitate strain dissemination and the spread of virulence traits [53,54,55].

Despite its significant findings, this study has some limitations. The analysis was restricted to two strains, which may not fully represent the genetic diversity of *V. harveyi* populations in aquaculture settings. Additionally, the virulence assays were conducted under controlled laboratory conditions, which may not fully replicate the complex environmental interactions in natural fish farms. The conjugation experiments, although rigorous, focused on a single recipient strain (45T2-KmR), limiting insights into plasmid transfer dynamics across diverse *Vibrio* strains. Future research should expand to include a broader range of *V. harveyi* isolates from different geographic regions and host species to better understand plasmid dissemination patterns.

In summary, this investigation highlights the crucial role of mobile genetic elements, particularly plasmids, in shaping the virulence and adaptability of *V. harveyi*. Our findings demonstrate that genomic plasticity, driven by these elements, contributes to the pathogenic diversity observed among *V. harveyi* strains. While the study focuses on a limited number of strains from a single host and location, future research should broaden the scope to include diverse strains, host species, and environmental contexts. Additionally, understanding plasmid-encoded gene functions and developing strategies to block the horizontal transfer of virulence factors are essential for improving disease management in marine aquaculture systems.

## Figures and Tables

**Table 1:** Bacterial strains and plasmids used for genetic manipulation in this study.

Strains/ Plasmids	Species/ Vector Strain	Description	References or Sources
N8T11	*V. harveyi*	Wild-type strain isolated from LYC liver	Yang et al. [20]
45T2	*V. harveyi*	Wild-type strain isolated from LYC kidney	Yang et al. [20]
45T2-KanR	*V. harveyi*	*V. harveyi* 45T2 strain carrying a kanamycin resistance marker via transposition	This work
DH5α	*E. coli*	Strain for general cloning	Vazyme
pUC18T-mini-Tn7T-Km	*E. coli*	Mini-Tn7 vector for chromosomal DNA integration; confers Ampicillin and Kanamycin resistance	Choi et al. [21]
pTNS2	*E. coli*	Helper plasmid, providing the Tn7 transposition function; confers Ampicillin resistance	Choi et al. [21]

**Table 2:** Genomic characteristics of *V. harveyi* strains N8T11 and 45T2.

Strain	Chromosome/Plasmid	Size (bp)	GC Content (%)	GenBank Accession No.	No. of Genes	No. of tRNA	No. of rRNA
N8T11	Chromosome 1	3,842,609	44.61	CP154266	3610	109	31
	Chromosome 2	2,274,153	44.93	CP154267	2063	16	3
	pN8T11a	97,019	41.97	CP154268	72	0	0
	pN8T11b	72,910	41.49	CP154269	66	0	0
	pN8T11c	61,668	43.86	CP154270	72	0	0
	pN8T11d	60,275	44.13	CP154271	59	0	0
	pN8T11e	2285	44.96	CP154272	1	0	0
45T2	Chromosome 1	3,555,107	45.05	CP154264	3298	110	34
	Chromosome 2	2,254,156	44.89	CP154265	1988	16	3

**Table 3:** Antibiotic resistance gene profiles in *V. harveyi* strains N8T11 and 45T2.

Gene Locus	Antibiotic Resistance Ontology Term	Detection Criteria	AMR Gene Family	Drug Class	Resistance Mechanism	Identity (%)	Reference Length (%)	Location
Shared resistance genes (present in both N8T11 and 45T2)								
AAIA70_01850/ AAIA71_15865	Fluoroquinolone-resistant *parE*	Protein variant	ParE	Fluoroquinolone antibiotics	Antibiotic target alteration	78.98	99.37	Chr 1
AAIA70_14670/ AAIA71_01910	Glycopeptide resistance gene cluster *vanT*	Protein homolog	VanT	Glycopeptide antibiotics	Altered cell wall precursor targets	32.5	50.7	Chr 1
AAIA70_14950/ AAIA71_01350	RND antibiotic efflux pump	Protein homolog	RND ^1^ family	Macrolide, fluoroquinolone, penam antibiotics	Antibiotic efflux	95.24	100	Chr 1
AAIA70_16780/ AAIA71_18920	RND antibiotic efflux pump	Protein homolog	AdeF	Fluoroquinolone, tetracycline antibiotics	Antibiotic efflux	43.25	99.15	Chr 2
AAIA70_06905/ AAIA71_10245	SMR antibiotic efflux pump	Protein homolog	QacG	Disinfecting agent antiseptics	Efflux pump for disinfectants	35.64	109.35	Chr 1
45T2-specific resistance genes								
AAIA70_05875	Tetracycline resistance ABC efflux pump	ABC model	TxR	Tetracycline antibiotics	Antibiotic efflux	85.76	99.37	Chr 1

Note: ^1^ Resistance-Nodulation-Division.

## Data Availability

The datasets generated and/or analyzed during the current study are available in the NCBI repository under the BioProject IDs SAMN41105143 and SAMN41104981.

## Supplementary Materials

The following supporting information can be downloaded at: https://www.mdpi.com/article/10.3390/microorganisms13051129/s1, Table S1. Genomic information of Vibrio strains included in this study; Table S2. Primers used in this study; Table S3. Predicted secretory system genes in *V. harveyi* strains N8T11 and 45T2; Table S4. Predicted secretory protein-encoding genes in *V. harveyi* strains N8T11 and 45T2; Table S5. Comparative analysis of predicted virulence genes in *V. harveyi* strains N8T11 and 45T2 based on VFDB; Table S6. Unique virulence genes identified in *V. harveyi* strain N8T11; Table S7. Genomic islands predicted in *V. harveyi* strains N8T11 and 45T2; Table S8. Insertion sequences predicted in *V. harveyi* strains N8T11 and 45T2; Table S9. Prophage regions and associated genes in *V. harveyi* strain N8T11; Table S10. Genetic features of plasmids in *V. harveyi* strain N8T11; Table S11. Plasmids from other sources similar to those found in N8T11.

## Abbreviations

The following abbreviations are used in this manuscript:

ANI	Average Nucleotide Identity
HGT	Horizontal Gene Transfer
PCR	Polymerase Chain Reaction
GIs	Genomic Islands
RND	Resistance Nodulation Division.
