# Supplementary material for: Comparative Genomic Analysis of Two Vibrio harveyi Strains from Larimichthys crocea with Divergent Virulence Profiles
Source: Microorganisms. 2025 May 14;13(5):1129. doi: 10.3390/microorganisms13051129 (PMC12114485; doi:10.3390/microorganisms13051129)
Supplement: Supplementary file 1 [file microorganisms-13-01129-s001.zip › microorganisms-3495592-supplementary.pdf]

**Table S1.** Genomic information of *Vibrio* strains included in this study

| Genome assembly | Strain | Species | Host / environmental origin | Geographic location |
|---|---|---|---|---|
| ASM1264146v1 | SM6 | *V. agarilyticus* | Seawater | China |
| ASM139945v2 | 605 | *V. bivalvicida* | Grooved carpet shell | Spain |
| ASM155843v2 | FDAARGOS_107 | *V. harveyi* | Environment | Bahamas |
| ASM190843v2 | QT520 | *V. harveyi* | Golden pompano | China |
| ASM19609v1 | RIMD 2210633 | *V. parahaemolyticus* | Human | Japan |
| ASM2139773v1 | XH2145 | *V. harveyi* | Seawater | China |
| ASM216375v1 | ATCC 25920 | *V. campbellii* | Seawater | USA |
| ASM222426v1 | ATCC 27562 | *V. vulnificus* | Estuarine | USA |
| ASM2365091v1 | E110 | *V. alginolyticus* | Pacific white shrimp | - |
| ASM2497193v1 | 1 | *V. harveyi* | Penaeus vannamei | China |
| ASM274198v1 | CAIM 577 | *V. rotiferianus* | Rotifer from water | Belgium |
| ASM284985v1 | LC2-005 | *V. azureus* | Seawater | Japan |
| ASM285029v1 | 345 | *V. harveyi* | Grouper | China |
| ASM2855131v1 | VH21FL | *V. harveyi* | Japanese flounder | South Korea |
| ASM2888554v1 | A2 | *V. harveyi* | European seabass | Croatia |

| | | | | |
|---|---|---|---|---|
| ASM2888556v1 | 120-19 | *V. harveyi* | Golden seabream | Croatia |
| ASM2888558v1 | 160-19 | *V. harveyi* | European seabass | Croatia |
| ASM2888560v1 | 94/17 | *V. harveyi* | European seabass | Croatia |
| ASM2889862v1 | A27 | *V. harveyi* | European seabass | Croatia |
| ASM2889864v1 | FR9 | *V. harveyi* | European seabass | France |
| ASM2889866v1 | ESP | *V. harveyi* | Golden seabream | Spain |
| ASM2889868v1 | FR5 | *V. harveyi* | European seabass | Tunisia |
| ASM2889868v1 | IT5 | *V. harveyi* | European seabass | Turkey |
| ASM2889870v1 | IT7 | *V. harveyi* | European seabass | Italy |
| ASM2889872v1 | IT2 | *V. harveyi* | European seabass | Italy |
| ASM2889874v1 | TUR2 | *V. harveyi* | Golden seabream | Turkey |
| ASM2889878v1 | IT8 | *V. harveyi* | European seabass | Italy |
| ASM2889880v1 | IT4 | *V. harveyi* | European seabass | Italy |
| ASM2889882v1 | FR10 | *V. harveyi* | Golden seabream | France |
| ASM2899353v1 | IT6 | *V. harveyi* | Golden seabream | Italy |
| ASM290647v1 | BoB-53 | *V. campbellii* | Marine environments | India |
| ASM290665v1 | 090810a | *V. hyugaens* | Seawater | Japan |
| ASM2942895v1 | PH1009 | *V. harveyi* | Grass shrimp | Philippines |

| | | | | |
|---|---|---|---|---|
| ASM3006043v1 | SB1 | *V. harveyi* | Asian seabass | India |
| ASM3038840v1 | K2014767 | *V. harveyi* | Caribbean spiny lobster | USA |
| ASM3635193v1 | WHSS0915 | *V. harveyi* | Korean rockfish | China |
| ASM3799714v1 | NH-LM1 | *V. harveyi* | Yellowsnout sea bass | China |
| ASM4269163v1 | LcV6 | *V. harveyi* | Large yellow croaker | China |
| ASM4284805v1 | TUMSAT-2019 | *V. harveyi* | Kuruma shrimp | Japan |
| ASM455152v1 | BEI176 | *V. ouci* | Seawater | China |
| ASM46443v1 | BAA-1116 | *V. campbellii* | Seawater | - |
| ASM46718v1 | NBRC 102218 | *V. ezurae* | Abalone | Japan |
| ASM653852v1 | NBRC 102082 | *V. inusitatus* | Red abalone | USA |
| ASM77011v2 | ATCC 33843 | *V. harveyi* | Marine environments | USA |
| ASM799093v1 | NBRC 104589 | *V. sagamiensis* | Seawater | Japan |
| ASM918474v1 | WXL538 | *V. harveyi* | Estuarine water | China |
| ASM936341v1 | THAF100 | *V.aquimaris* | Water | Germany |
| ASM966531v1 | 2011V-1164 | *V. harveyi* | Brown shark | USA |
| S2394 | S2394 | *V. nigripulchritudo* | Jellyfish | Australia |
| S2757 | S2757 | *V. galatheae* | Mussel | Australia |
| Vsuperstes | JCM 21480 | *V. superstes* | Red Abalone | Australia |

**Table S2.** Bacterial strains and plasmids used for genetic manipulation in this study

| Primer | Sequence (5'-3') | Description |
|---|---|---|
| 45T2-F-*ompF* | AATGGGGCTACAACTCTGGC | To amplify a 337-bp gene fragment of *ompF* |
| 45T2-R-*ompF* | TTCAGTGAGGCTGTCGCATT | |
| N8T11-F-*moxR* | AGGAGCTCAGCGCGTTATAC | To amplify a 668-bp gene fragment of *moxR* |
| N8T11-R-*moxR* | TTCCATCCAAGGGGGATTGC | |
| Plasmid conjugation efficiency and stability | | |
| pN8T11a-*yejB*-F | TGTGCTCCGCAGGCTCTTAT | To amplify a 372- gene fragment of *yejB* |
| pN8T11a-*yejB*-R | AATGTCCACCCACCCAATGA | |
| pN8T11b-*OmpA*-F | TTTCTCGTTGGGTCTTATCGT | To amplify a 499-bp gene fragment of *OmpA* |
| pN8T11b-*OmpA*-R | GCTCACTTCGTCTGTTCCTTG | |
| pN8T11c-AAIA71_29215-F | ATCCCTCTCACATTACACCA | To amplify a 264-bp gene fragment at gene locus AAIA71_29215 |
| pN8T11c-AAIA71_29215-R | CATAACTAACCAAGCCAACA | |
| pN8T11d-*tbpA*-F | GTGAAGGAGAACGAACGAGA | To amplify a 764- bp gene fragment of *tbpA* |
| pN8T11d-*tbpA*-R | AAATGGACTAACCGACCAAG | |
| pN8T11e-*abiQ*-F | AGAGGGGACAAGCGGAAAAGGA | To amplify a 238-bp gene fragment of *abiQ* |
| pN8T11e-*abiQ*-R | GCCGAGCGGAAGAGGTAAATGA | |

**Table S3.** Predicted secretory system genes in *V. harveyi* strains N8T11 and 45T2

| Gene name | Secretion system type | Gene Count | N8T11 Gene Locus | 45T2 Gene Locus |
|---|---|---|---|---|
| *tolC* | Type I | 2 | AAIA71_06635/ AAIA71_08980 | AAIA70_01825/ AAIA70_07235 |
| *gspM* | Type II | 1 | AAIA71_17160 | AAIA70_14505 |
| *gspL* | Type II | 1 | AAIA71_17165 | AAIA70_00595 |
| *gspK* | Type II | 1 | AAIA71_17170 | AAIA70_00590 |
| *gspJ* | Type II | 1 | AAIA71_17175 | AAIA70_00585 |
| *gspI* | Type II | 1 | AAIA71_17180 | AAIA70_00580 |
| *gspH* | Type II | 1 | AAIA71_17185 | AAIA70_00575 |
| *gspG* | Type II | 1 | AAIA71_17190 | AAIA70_00570 |
| *gspF* | Type II | 1 | AAIA71_17195 | AAIA70_00565 |
| *gspE* | Type II | 1 | AAIA71_17200 | AAIA70_00560 |
| *gspD* | Type II | 1 | AAIA71_17205 | AAIA70_00555 |
| *gspC* | Type II | 1 | AAIA71_17210 | AAIA70_00550 |
| *yscV* | Type III | 1 | AAIA71_12100 | AAIA70_05390 |
| *yscX* | Type III | 1 | AAIA71_12110 | AAIA70_05380 |
| *yscW* | Type III | 1 | AAIA71_12125 | AAIA70_05365 |
| *yscN* | Type III | 1 | AAIA71_12130 | AAIA70_05360 |
| *yscO* | Type III | 1 | AAIA71_12135 | AAIA70_05355 |
| *yscP* | Type III | 1 | AAIA71_12140 | AAIA70_05350 |
| *yscQ* | Type III | 1 | AAIA71_12145 | AAIA70_05345 |
| *yscR* | Type III | 1 | AAIA71_12150 | AAIA70_05340 |
| *yscS* | Type III | 1 | AAIA71_12155 | AAIA70_05335 |
| *yscT* | Type III | 1 | AAIA71_12160 | AAIA70_05330 |
| *yscU* | Type III | 1 | AAIA71_12165 | AAIA70_05325 |
| *yscL* | Type III | 1 | AAIA71_12200 | AAIA70_05290 |
| *yscJ* | Type III | 1 | AAIA71_12210 | AAIA70_05280 |
| *yscF* | Type III | 1 | AAIA71_12230 | AAIA70_05260 |
| *yscC* | Type III | 1 | AAIA71_12245 | AAIA70_05245 |
| *virD4* | Type IV | 1 | AAIA71_05975 | AAIA70_11270 |
| *virB11* | Type IV | 1 | AAIA71_05980 | AAIA70_11265 |
| *virB10* | Type IV | 1 | AAIA71_05990 | AAIA70_11255 |
| *virB9* | Type IV | 1 | AAIA71_05995 | AAIA70_11250 |
| *virB8* | Type IV | 1 | AAIA71_06000 | AAIA70_11245 |
| *virB6* | Type IV | 1 | AAIA71_06005 | AAIA70_11235 |
| *virB5* | Type IV | 1 | AAIA71_06015 | AAIA70_11225 |
| *virB4* | Type IV | 1 | AAIA71_06020 | AAIA70_11220 |
| *virB1* | Type IV | 1 | AAIA71_06035 | AAIA70_11205 |
| *vgrG* | Type VI | 3 | AAIA71_10000/ AAIA71_19525/ AAIA71_21160 | AAIA70_07145/ AAIA70_24455/ AAIA70_26120 |
| *hcp* | Type VI | 3 | AAIA71_10020/ AAIA71_19520/ AAIA71_21165 | AAIA70_06150/ AAIA70_24450/ AAIA70_26125 |
| *vasG* | Type VI | 3 | AAIA71_10055/ AAIA71_19515/ AAIA71_21170 | AAIA70_07090/ AAIA70_24445/ AAIA70_26130 |
| *vasD* | Type VI | 3 | AAIA71_10070/ AAIA71_19610/ AAIA71_21245 | AAIA70_07075/ AAIA70_24375/ AAIA70_26035 |

| | | | | |
|---|---|---|---|---|
| *impK* | Type VI | 3 | AAIA71_10080/ AAIA71_19620/ AAIA71_21235 | AAIA70_07065/ AAIA70_24365/ AAIA70_26025 |
| *impL* | Type VI | 3 | AAIA71_10085/ AAIA71_19590/ AAIA71_21230 | AAIA70_07060/ AAIA70_24360/ AAIA70_26020 |
| *secA* | Sec-SRP | 1 | AAIA71_15700 | AAIA70_02015 |
| *secB* | Sec-SRP | 1 | AAIA71_01135 | AAIA70_15165 |
| *secD* | Sec-SRP | 2 | AAIA71_14935/ AAIA71_18815 | AAIA70_02660/ AAIA70_16885 |
| *secE* | Sec-SRP | 1 | AAIA71_00715 | AAIA70_15585 |
| *secF* | Sec-SRP | 2 | AAIA71_14930/ AAIA71_18820 | AAIA70_16880/ AAIA70_02665 |
| *secG* | Sec-SRP | 1 | AAIA71_03945 | AAIA70_13175 |
| *secY* | Sec-SRP | 1 | AAIA71_14245 | AAIA70_01265 |
| *yidC* | Sec-SRP | 1 | AAIA71_18020 | AAIA70_16460 |
| *yajC* | Sec-SRP | 1 | AAIA71_14940 | AAIA70_02655 |
| *ffh* | Sec-SRP | 1 | AAIA71_03590 | AAIA70_13530 |
| *ftsY* | Sec-SRP | 1 | AAIA71_00500 | AAIA70_15800 |
| *tatA* | Tat | 1 | AAIA71_17355 | AAIA70_00405 |
| *tatB* | Tat | 1 | AAIA71_17350 | AAIA70_00410 |
| *tatC* | Tat | 1 | AAIA71_17345 | AAIA70_00415 |

**Table S4**. Predicted secretory protein-Encoding genes in *Vibrio harveyi* strains N8T11 and 45T2

| Gene locus | Stain | Source (Plasmid/Chromosome) | Start position | End position | Orientation | Protein description |
|---|---|---|---|---|---|---|
| AAIA70_00370 | 45T2 | Chromosome 1 | 83616 | 83176 | - | Hypothetical protein |
| AAIA70_00985 | 45T2 | Chromosome 1 | 221577 | 221353 | - | Hypothetical protein |
| AAIA70_05685 | 45T2 | Chromosome 1 | 1240716 | 1243154 | + | Collagenase |
| AAIA70_06800 | 45T2 | Chromosome 1 | 1501296 | 1501865 | + | Hypothetical protein |
| AAIA70_06805 | 45T2 | Chromosome 1 | 1502450 | 1505194 | + | TcfC E-set like domain-containing protein |
| AAIA70_06810 | 45T2 | Chromosome 1 | 1505198 | 1505947 | + | Hypothetical protein |
| AAIA70_06815 | 45T2 | Chromosome 1 | 1505944 | 1507038 | + | Hypothetical protein |
| AAIA70_06820 | 45T2 | Chromosome 1 | 1508380 | 1508153 | - | Hypothetical protein |
| AAIA70_08250 | 45T2 | Chromosome 1 | 1840511 | 1839510 | - | Haloacid dehalogenase-like hydrolase |
| AAIA70_08255 | 45T2 | Chromosome 1 | 1841842 | 1840736 | - | BatD family protein |
| AAIA70_08285 | 45T2 | Chromosome 1 | 1847035 | 1848681 | + | Sulfatase-like hydrolase/transferase |
| AAIA70_08290 | 45T2 | Chromosome 1 | 1848745 | 1849101 | + | Hypothetical protein |
| AAIA70_08300 | 45T2 | Chromosome 1 | 1850550 | 1851545 | + | Hypothetical protein |
| AAIA70_08305 | 45T2 | Chromosome 1 | 1851553 | 1851951 | + | Hypothetical protein |
| AAIA70_08335 | 45T2 | Chromosome 1 | 1856654 | 1855842 | - | Hypothetical protein |
| AAIA70_09370 | 45T2 | Chromosome 1 | 2075894 | 2075403 | - | Hypothetical protein |
| AAIA70_09865 | 45T2 | Chromosome 1 | 2145549 | 2145022 | - | Hypothetical protein |
| AAIA70_11855 | 45T2 | Chromosome 1 | 2567674 | 2567928 | + | CG2 omega domain protein |
| AAIA70_19270 | 45T2 | Chromosome 2 | 593152 | 594237 | + | Porin |
| AAIA70_23935 | 45T2 | Chromosome 2 | 1633406 | 1631643 | - | DUF3859 domain-containing protein |
| AAIA70_24605 | 45T2 | Chromosome 2 | 1789658 | 1792414 | + | NEW3 domain-containing protein |
| AAIA71_01500 | N8T11 | Chromosome 1 | 315503 | 316345 | + | BspA family leucine-rich repeat surface protein |
| AAIA71_01640 | N8T11 | Chromosome 1 | 344035 | 346113 | + | Hypothetical protein |
| AAIA71_01645 | N8T11 | Chromosome 1 | 346216 | 346746 | + | Hypothetical protein |

| | | | | | | |
|---|---|---|---|---|---|---|
| AAIA71_01665 | N8T11 | Chromosome 1 | 349932 | 350246 | + | Hypothetical protein |
| AAIA71_01670 | N8T11 | Chromosome 1 | 350248 | 350601 | + | Hypothetical protein |
| AAIA71_02565 | N8T11 | Chromosome 1 | 538472 | 539089 | + | Hypothetical protein |
| AAIA71_02600 | N8T11 | Chromosome 1 | 549581 | 547257 | - | Ig-like domain-containing protein |
| AAIA71_03435 | N8T11 | Chromosome 1 | 691274 | 691912 | + | OmpA family protein |
| AAIA71_05940 | N8T11 | Chromosome 1 | 1242891 | 1242160 | - | Peptidase |
| AAIA71_07565 | N8T11 | Chromosome 1 | 1581521 | 1581892 | + | Hypothetical protein |
| AAIA71_07585 | N8T11 | Chromosome 1 | 1583764 | 1584237 | + | DUF4476 domain-containing protein |
| AAIA71_09460 | N8T11 | Chromosome 1 | 1984583 | 1983093 | - | Hypothetical protein |
| AAIA71_09465 | N8T11 | Chromosome 1 | 1984994 | 1984752 | - | Hypothetical protein |
| AAIA71_09465 | N8T11 | Chromosome 1 | 2102555 | 2101011 | - | Trypsin-like serine protease |
| AAIA71_10425 | N8T11 | Chromosome 1 | 2212106 | 2212540 | + | Hypothetical protein |
| AAIA71_10490 | N8T11 | Chromosome 1 | 2228147 | 2228644 | + | Hypothetical protein |
| AAIA71_11035 | N8T11 | Chromosome 1 | 2360605 | 2361429 | + | Hypothetical protein |
| AAIA71_11490 | N8T11 | Chromosome 1 | 2455271 | 2455813 | + | Hypothetical protein |
| AAIA71_11565 | N8T11 | Chromosome 1 | 2468065 | 2468469 | - | Hypothetical protein |
| AAIA71_11570 | N8T11 | Chromosome 1 | 2469245 | 2468931 | - | Hypothetical protein |
| AAIA71_11575 | N8T11 | Chromosome 1 | 2470317 | 2469367 | - | Hypothetical protein |
| AAIA71_11595 | N8T11 | Chromosome 1 | 2473935 | 2473405 | - | Hypothetical protein |
| AAIA71_11600 | N8T11 | Chromosome 1 | 2476116 | 2474038 | - | Hypothetical protein |
| AAIA71_11605 | N8T11 | Chromosome 1 | 2479347 | 2476267 | - | Hypothetical protein |
| AAIA71_11735 | N8T11 | Chromosome 1 | 2504637 | 2503795 | - | BspA family leucine-rich repeat surface protein |
| AAIA71_11930 | N8T11 | Chromosome 1 | 2556683 | 2555769 | - | Alpha/beta hydrolase fold domain-containing protein |
| AAIA71_12010 | N8T11 | Chromosome 1 | 2570080 | 2570760 | + | DUF1007 family protein |
| AAIA71_13675 | N8T11 | Chromosome 1 | 2933387 | 2933569 | + | Hypothetical protein |
| AAIA71_14570 | N8T11 | Chromosome 1 | 3113280 | 3112993 | - | Hypothetical protein |
| AAIA71_14675 | N8T11 | Chromosome 1 | 3128436 | 3128945 | + | Hypothetical protein |

| | | | | | | |
|---|---|---|---|---|---|---|
| AAIA71_16605 | N8T11 | Chromosome 1 | 3534513 | 3535670 | + | Hypothetical protein |
| AAIA71_16790 | N8T11 | Chromosome 1 | 3568514 | 3568723 | + | Hypothetical protein |
| AAIA71_17410 | N8T11 | Chromosome 1 | 3705798 | 3705313 | - | Hypothetical protein |
| AAIA71_20510 | N8T11 | Chromosome 2 | 591190 | 589589 | - | Hypothetical protein |
| AAIA71_20515 | N8T11 | Chromosome 2 | 591550 | 591320 | - | Membrane protein |
| AAIA71_20560 | N8T11 | Chromosome 2 | 597959 | 596367 | - | Methyl-accepting chemotaxis protein |
| AAIA71_20565 | N8T11 | Chromosome 2 | 598323 | 598093 | - | Membrane protein |
| AAIA71_20780 | N8T11 | Chromosome 2 | 640673 | 640209 | - | Hypothetical protein |
| AAIA71_20785 | N8T11 | Chromosome 2 | 641218 | 640712 | - | Hypothetical protein |
| AAIA71_20790 | N8T11 | Chromosome 2 | 641679 | 641221 | - | Hypothetical protein |
| AAIA71_20930 | N8T11 | Chromosome 2 | 642024 | 641683 | - | Nitrite reductase small subunit NirD |
| AAIA71_20975 | N8T11 | Chromosome 2 | 681048 | 682094 | + | Hypothetical protein |
| AAIA71_21710 | N8T11 | Chromosome 2 | 846848 | 847672 | + | DUF2861 family protein |
| AAIA71_21715 | N8T11 | Chromosome 2 | 847746 | 850016 | + | Glycoside hydrolase |
| AAIA71_23075 | N8T11 | Chromosome 2 | 1145970 | 1145494 | - | Hypothetical protein |
| AAIA71_23080 | N8T11 | Chromosome 2 | 1148864 | 1146048 | - | Insulinase family protein |
| AAIA71_23090 | N8T11 | Chromosome 2 | 1152982 | 1150637 | - | TonB-dependent receptor plug domain-containing protein |
| AAIA71_23095 | N8T11 | Chromosome 2 | 1153502 | 1154245 | + | DUF3450 domain-containing protein |
| AAIA71_23120 | N8T11 | Chromosome 2 | 1157129 | 1158304 | + | Tetratricopeptide repeat protein |
| AAIA71_28040 | N8T11 | Chromosome 2 | 2203612 | 2202659 | - | DUF2860 family protein |
| AAIA71_28125 | N8T11 | Chromosome 2 | 2216275 | 2215937 | - | Cardiolipin synthetase domain protein |
| AAIA71_28410 | N8T11 | pN8T11a | 7479 | 9290 | + | extracellular solute-binding protein |
| AAIA71_28445 | N8T11 | pN8T11a | 17295 | 14998 | - | TonB-dependent siderophore receptor |
| AAIA71_28525 | N8T11 | pN8T11a | 56846 | 56157 | - | Hypothetical protein |
| AAIA71_28740 | N8T11 | pN8T11b | 2301 | 2921 | + | OmpA family protein |
| AAIA71_28910 | N8T11 | pN8T11b | 31002 | 31538 | + | Hypothetical protein |
| AAIA71_29110 | N8T11 | pN8T11c | 13369 | 13920 | + | outer membrane beta-barrel protein |

| | | | | | | |
|---|---|---|---|---|---|---|
| AAIA71_29345 | N8T11 | pN8T11c | 47795 | 48151 | + | Hypothetical protein |
| AAIA71_29520 | N8T11 | pN8T11d | 24277 | 23744 | - | Hypothetical protein |
| AAIA71_29705 | N8T11 | pN8T11d | 58616 | 58230 | - | Hydroxyisourate hydrolase |

**Table S5**. Comparative analysis of predicted virulence genes in V. harveyi strains N8T11 and 45T2 based on VFDB

| Gene ontology category | Function | Virulence Genes in N8T11 | Gene Count in N8T11 | Virulence Genes in 45T2 | Gene Count in 45T2 | Presence |
|---|---|---|---|---|---|---|
| Mannose-sensitive Hemagglutinin | Adhesion and Biofilm Formation | *mshA, mshD, mshE, mshF, mshG, mshH, mshI, mshJ,mshK, mshL, mshM, mshN* | 12 | *mshA, mshB, mshD, mshE, mshF, mshG, mshH, mshI, mshJ, mshK, mshL, mshM, mshN* | 13 | Both |
| Type IV Pilus | Assembly and Motility | *pilB, pilC, pilD* | 3 | *pilB, pilC, pilD* | 3 | Both |
| LPS O-antigen | Surface Antigen Variation | - | 0 | *wbpM* | 1 | 45T2 |
| The tad Locus | Biofilm Formation | - | 0 | *tadA* | 1 | 45T2 |
| Capsular Polysaccharide | Protective Capsule Formation | *cpsA, cpsB, cpsC, cpsD, cpsE, cpsF, cpsG, cpsH, cpsI, cpsJ, wbfV/wcvB, wecA, wza, wzb, wzc* | 15 | *cpsA, cpsB, cpsC, cpsD, cpsE, cpsF, cpsG, cpsH, cpsI, cpsJ, wbfV/wcvB, wbfY, wza, wzb, wzc* | 15 | Both |
| Flagellar Assembly | Motility and Sensory Functions | *cheA, cheB, cheR, cheV, cheW, cheY, cheZ, filM,* | 53 | *cheA, cheB, cheR, cheV, cheW, cheY,* | 53 | Both |

| | | | | | | |
|---|---|---|---|---|---|---|
| | | *flaA, flaB, flaC, flaD, flaE, flaG, flaI, flgA, flgB, flgC, flgD, flgE, flgF, flgG, flgH, flgI, flgJ, flgK, flgL, flgM, flgN, flhA, flhB, flhF, flhG, fliA, fliD, fliE, fliF, fliG, fliH, fliI, fliJ, fliK, fliL, fliN, fliO, fliP, fliQ, fliR, fliS, flrA, flrB, flrC, motA, motB, motX, motY* | | *cheZ, filM, flaA, flaB, flaC, flaD, flaE, flaG, flaI, flgA, flgB, flgC, flgD, flgE, flgF, flgG, flgH, flgI, flgJ, flgK, flgL, flgM, flgN, flhA, flhB, flhF, flhG, fliA, fliD, fliE, fliF, fliG, fliH, fliI, fliJ, fliK, fliL, fliN, fliO, fliP, fliQ, fliR, fliS, flrA, flrB, flrC, motA, motB, motX, motY* | | |
| Enterobactin Receptors | Iron Uptake | *vctA* | 1 | *vctA* | 1 | Both |
| Heme Receptors | Iron Acquisition | *hutA, hutR* | 2 | *hutA, hutR* | 2 | Both |
| Periplasmic Binding Protein-dependent ABC Transport Systems | Nutrient Transport | *vctC, vctD, vctG, vctP, vibE* | 5 | *vctC, vctD, vctG, vctP, vibE* | 5 | Both |
| Ferrous Iron Transport | Iron Transport | *sitC* | 1 | *sitC* | 1 | Both |
| Iron/Manganese Transport | Metal Ion Transport | *sitA, sitB, sitD* | 3 | *sitA, sitB, sitD* | 3 | Both |
| Autoinducer-2 | Quorum Sensing | *luxS* | 1 | *luxS* | 1 | Both |
| Cholerae Autoinducer-1 | Quorum Sensing | *cqsA* | 1 | *cqsA* | 1 | Both |

| | | | | | | |
|---|---|---|---|---|---|---|
| EPS Type II Secretion System | Extracellular Protein Secretion | *epsC, epsE, epsF, epsG, epsH, epsI, epsJ, epsK, epsL, epsM, epsN, gspD* | 12 | *epsC, epsE, epsF, epsG, epsH, epsI, epsJ, epsK, epsL, epsM, epsN, gspD* | 12 | Both |
| T3SS1 Secreted Effectors | Host Interaction and Evasion | *vopQ, vopR, ati2* | 3 | *vopQ, vopR, ati2* | 3 | Both |
| Type III serection system | Host Interaction and Evasion | *sycN, tyeA, vcrD, vcrG, vcrH, vcrR, virF, vopB, vopD, vopN, vscA, vscB, vscC, vscD, vscF, vscG, vscH, vscI, vscJ, vscK, vscL, vscN, vscO, vscQ, vscR, vscS, vscT, vscU, vscX, vscY, vxsC* | 30 | *sycN, tyeA, vcrD, vcrG, vcrH, vcrR, virF, vopB, vopD, vopN, vscA, vscB, vscC, vscD, vscF, vscG, vscH, vscI, vscJ, vscK, vscL, vscN, vscO, vscQ, vscR, vscS, vscT, vscU, vscX, vscY, vxsC* | 30 | Both |
| Heat-stable Cytotonic Enterotoxin | Toxin Production | *ast* | 1 | *ast* | 1 | Both |
| Urease | Urea Hydrolysis | *ureB, ureG* | 2 | *ureB, ureG* | 2 | Both |
| Trehalose-recycling | Sugar Utilization | *sugC* | 1 | *sugC* | 1 | Both |
| LPS Glucosylation | Surface Antigen Modification | *gtrB* | 1 | - | 0 | N8T11 |
| Capsule | Capsule Formation | - | 0 | *cap8E, cap8G* | 2 | 45T2 |

| | | | | | | |
|---|---|---|---|---|---|---|
| LOS | Lipooligosaccharide Synthesis | - | 0 | *EIIC* | 1 | 45T2 |

**Table S6.** Unique virulence genes identified in V. harveyi strain N8T11

| Gene locus | Location | Virulence Factor | Start Position | End Position | Description | E-value |
|---|---|---|---|---|---|---|
| AAIA71_02600 | Chromosome 1 | VpadF | 549581 | 547257 | Ig-like domain-containing protein | 3.60E-45 |
| AAIA71_03345 | Chromosome 1 | Hemolysin | 673697 | 675712 | Peptidase domain-containing ABC transporter | 1.30E-70 |
| AAIA71_03405 | Chromosome 1 | Flp pili | 686146 | 687048 | Type II secretion system F family protein | 2.10E-49 |
| AAIA71_03410 | Chromosome 1 | Flp pili | 687045 | 687887 | Type II secretion system F family protein | 1.40E-42 |
| AAIA71_03435 | Chromosome 1 | Polar flagella | 691274 | 691912 | OmpA family protein | 1.20E-06 |
| AAIA71_05815 | Chromosome 1 | LetA/S | 1218295 | 1216499 | ATP-binding protein | 1.10E-44 |
| AAIA71_09450 | Chromosome 1 | Zot | 1982742 | 1981357 | Zonular occludens toxin domain-containing protein | 2.70E-11 |
| AAIA71_11935 | Chromosome 1 | Ent | 2558602 | 2556683 | TonB-dependent receptor | 2.90E-24 |
| AAIA71_11975 | Chromosome 1 | Hemolysin/ Cytolysin | 2567516 | 2566668 | Dipeptide/oligopeptide/nickel ABC transporter ATP-binding protein | 7.80E-22 |
| AAIA71_11980 | Chromosome 1 | Acinetobactin | 2568298 | 2567537 | ATP-binding cassette domain-containing protein | 8.80E-17 |
| AAIA71_14610 | Chromosome 1 | F1 antigen | 3118592 | 3117741 | AraC family transcriptional regulator | 8.40E-08 |
| AAIA71_14635 | Chromosome 1 | Capsule | 3121637 | 3122350 | TIGR04283 family arsenosugar biosynthesis glycosyltransferase | 2.00E-07 |
| AAIA71_14685 | Chromosome 1 | ACF | 3131358 | 3133004 | Methyl-accepting chemotaxis protein | 1.90E-32 |
| AAIA71_16685 | Chromosome 1 | Hyaluronic acid capsule | 3548212 | 3547295 | UTP--glucose-1-phosphate uridylyl transferase GalU | 4.90E-62 |

| | | | | | | |
|---|---|---|---|---|---|---|
| AAIA71_16695 | Chromosome 1 | LOS | 3549595 | 3550677 | Nucleotide sugar dehydrogenase | 5.00E-82 |
| AAIA71_16750 | Chromosome 1 | Capsule | 3560491 | 3559526 | Glycosyl transferase family 2 protein | 3.50E-26 |
| AAIA71_16835 | Chromosome 1 | LPS | 3578543 | 3577671 | Glycosyltransferase family A protein | 2.00E-17 |
| AAIA71_16975 | Chromosome 1 | LPS | 3605009 | 3604101 | Glycosyltransferase | 8.20E-86 |
| AAIA71_18460 | Chromosome 2 | PhoP | 846201 | 846851 | LysR family transcriptional regulator | 1.20E-22 |
| AAIA71_23045 | Chromosome 2 | PDIM | 1139861 | 1140910 | Alcohol dehydrogenase family protein | 6.90E-20 |
| AAIA71_23050 | Chromosome 2 | TTSS secreted effectors | 1141298 | 1142281 | YopT-type cysteine protease domain-containing protein | 2.60E-37 |
| AAIA71_23085 | Chromosome 2 | LOS | 1150564 | 1148861 | ABC transporter ATP-binding protein/permease | 1.00E-12 |
| AAIA71_26175 | Chromosome 2 | Pyoverdine | 1831214 | 1830324 | LysR family transcriptional regulator | 2.40E-37 |
| AAIA71_28125 | Chromosome 2 | Phospholipase D | 2216275 | 2215937 | Phospholipase D family protein | 3.10E-06 |
| AAIA71_28405 | pN8T11a | Pyoverdine | 5830 | 7479 | Cyclic peptide export ABC transporter | 7.40E-77 |
| AAIA71_28430 | pN8T11a | HitABC | 11396 | 12631 | ABC transporter permease subunit | 4.30E-29 |
| AAIA71_28450 | pN8T11a | Pyoverdine | 17530 | 17330 | MbtH family NRPS accessory protein | 2.50E-16 |
| AAIA71_28460 | pN8T11a | Pyoverdine | 18631 | 25419 | Non-ribosomal peptide synthetase | 9.30E-142 |
| AAIA71_28465 | pN8T11a | Pyoverdine | 25423 | 31698 | Amino acid adenylation domain-containing protein | 3.30E-226 |
| AAIA71_28475 | pN8T11a | Pyoverdine | 32142 | 44468 | Amino acid adenylation domain-containing protein | 0 |
| AAIA71_28480 | pN8T11a | Bacillibactin | 44556 | 46109 | Thioesterase domain-containing protein | 2.30E-51 |

| | | | | | | |
|---|---|---|---|---|---|---|
| AAIA71_28965 | pN8T11B | Ysa TTSS | 45654 | 45409 | Lytic trans glycosylase domain-containing protein | 4.10E-08 |
| AAIA71_29090 | pN8T11C | TTSS | 10940 | 10512 | Lytic trans glycosylase domain-containing protein | 7.60E-18 |
| AAIA71_29420 | pN8T11d | HmbR | 230 | 2455 | TonB-dependent hemoglobin/transferrin/lactoferrin family receptor | 1.10E-83 |
| AAIA71_29470 | pN8T11d | Ysa TTSS | 10155 | 10586 | Lytic trans glycosylase domain-containing protein | 1.10E-16 |

**Table S7.** Genomic islands predicted in *V. harveyi* strains N8T11 and 45T2

| Location | Start position | End position | GI name |
|---|---|---|---|
| N8T11 Chromosome 1 | 312237 | 351141 | N8T11 GI 1 |
| | 351932 | 367700 | N8T11 GI 2 |
| | 411820 | 423427 | N8T11 GI 3 |
| | 530789 | 556400 | N8T11 GI 4 |
| | 583541 | 595635 | N8T11 GI 5 |
| | 671331 | 682745 | N8T11 GI 6 |
| | 963073 | 971710 | N8T11 GI 7 |
| | 1181987 | 1252983 | N8T11 GI 8 |
| | 1548668 | 1642056 | N8T11 GI 9 |
| | 2456308 | 2472317 | N8T11 GI 10 |
| | 2473026 | 2508099 | N8T11 GI 11 |
| | 3108541 | 3135004 | N8T11 GI 12 |
| | 3221653 | 3235910 | N8T11 GI 13 |
| | 3551595 | 3566345 | N8T11 GI 14 |
| | 3704921 | 3714607 | N8T11 GI 15 |
| | 3718309 | 3728884 | N8T11 GI 16 |
| N8T11 Chromosome 2 | 474677 | 488757 | N8T11 GI 17 |
| | 717646 | 726961 | N8T11 GI 18 |
| | 727699 | 737103 | N8T11 GI 19 |
| | 740314 | 749618 | N8T11 GI 20 |
| | 1217352 | 1225974 | N8T11 GI 21 |
| | 2207652 | 2217359 | N8T11 GI 22 |
| pN8T11a | 3943 | 12409 | N8T11 GI 23 |
| | 47703 | 55202 | N8T11 GI 24 |
| | 81575 | 97019 | N8T11 GI 25 |
| pN8T11b | 45200 | 58755 | N8T11 GI 26 |
| pN8T11c | 22090 | 61668 | N8T11 GI 27 |
| pN8T11d | 35490 | 60275 | N8T11 GI 28 |
| 45T2 Chromosome 1 | 225746 | 242784 | 45T2 GI 1 |
| | 1127944 | 1138711 | 45T2 GI 2 |
| | 1501983 | 1512297 | 45T2 GI 3 |
| | 2069013 | 2153741 | 45T2 GI 4 |
| | 2450476 | 2490353 | 45T2 GI 5 |
| | 2692292 | 2700959 | 45T2 GI 6 |
| 45T2 Chromosome 2 | 315689 | 326652 | 45T2 GI 7 |
| | 1585249 | 1601259 | 45T2 GI 8 |
| | 1734700 | 1744005 | 45T2 GI 9 |
| | 1747216 | 1755355 | 45T2 GI 10 |
| | 1756093 | 1765408 | 45T2 GI 11 |
| | 1986151 | 2017027 | 45T2 GI 12 |

Table S8.  IS predicted in *V. harveyi* strains N8T11 and 45T2

| Stain | Start position | End position | Subject | Identity (%) | E-value |
|---|---|---|---|---|---|
| | 1772657 | 1772727 | ISPsy43 | 87.32 | 3.00E-08 |
| | 1724608 | 1724747 | Vnal | 82.27 | 4.00E-07 |
| | 1988387 | 1988841 | ISShes8 | 79.43 | 9.00E-29 |
| | 1988241 | 1988841 | ISShes8 | 90.2 | 4.00E-06 |
| | 1988645 | 1988291 | ISShwo2 | 84.08 | 2.00E-26 |
| | 1988241 | 1988287 | ISShwo2 | 91.49 | 4.00E-06 |
| 45T2 | 2015212 | 2015299 | ISVch8 | 88.64 | 3.00E-16 |
| | 2015142 | 2015183 | ISVch8 | 95.24 | 7.00E-08 |
| | 2015142 | 2015208 | ISV-N52 | 91.04 | 3.00E-13 |
| | 2015212 | 2015299 | ISV-N52 | 84.09 | 1.00E-06 |
| | 1879896 | 1879951 | ISVbsp5 | 92.86 | 2.00E-11 |
| | 1879906 | 1879948 | ISVnal | 93.02 | 4.00E-06 |
| | 1988391 | 1988431 | ISSham1 | 92.68 | 7.00E-05 |
| | 2449063 | 2450305 | ISVal2 | 93.4 | 0 |
| | 1564077 | 1565319 | ISVal2 | 93.4 | 0 |
| | 1593553 | 1594795 | ISVal2 | 93.4 | 0 |
| | 1620799 | 1622041 | ISVal2 | 93.4 | 0 |
| | 1570825 | 1572067 | ISVal2 | 93.4 | 0 |
| | 1562503 | 1563745 | ISVal2 | 93.4 | 0 |
| | 2480679 | 2481921 | ISVal2 | 93.4 | 0 |
| | 1204502 | 1205744 | ISVal2 | 93.4 | 0 |
| | 1201467 | 1202709 | ISVal2 | 93.4 | 0 |
| | 320476 | 321718 | ISVal2 | 93.4 | 0 |
| | 3699299 | 3700283 | ISVa2 | 91.68 | 0 |
| | 3561154 | 3562138 | ISVa2 | 91.68 | 0 |
| | 3554199 | 3555183 | ISVa2 | 91.68 | 0 |
| | 3341688 | 3342672 | ISVa2 | 91.68 | 0 |
| | 504763 | 505747 | ISVa2 | 91.68 | 0 |
| | 549839 | 550823 | ISVa2 | 91.68 | 0 |
| | 672689 | 673673 | ISVa2 | 91.68 | 0 |
| | 679750 | 680734 | ISVa2 | 91.68 | 0 |
| | 854172 | 855156 | ISVa2 | 91.68 | 0 |
| | 865510 | 866494 | ISVa2 | 91.68 | 0 |
| | 1234252 | 1235236 | ISVa2 | 91.68 | 0 |
| | 2559102 | 2560086 | ISVa2 | 91.68 | 0 |
| | 2464325 | 2465309 | ISVa2 | 91.68 | 0 |
| | 1568882 | 1569866 | ISVa2 | 91.68 | 0 |
| | 1605599 | 1606583 | ISVa2 | 91.68 | 0 |
| | 1616556 | 1617540 | ISVa2 | 91.68 | 0 |
| | 1990617 | 1991601 | ISVa2 | 91.68 | 0 |
| | 2285003 | 2285987 | ISVa2 | 91.68 | 0 |
| | 1522257 | 1523241 | ISVa2 | 91.68 | 0 |
| | 2506075 | 2507059 | ISVa2 | 91.68 | 0 |
| | 1243071 | 1244055 | ISVa2 | 91.68 | 0 |
| | 1213094 | 1214078 | ISVa2 | 91.68 | 0 |
| | 1202902 | 1203886 | ISVa2 | 91.68 | 0 |
| | 2698900 | 2699884 | ISVa2 | 91.68 | 0 |
| | 2933655 | 2934639 | ISVa2 | 91.68 | 0 |
| | 3090663 | 3091647 | ISVa2 | 91.68 | 0 |

| | | | | |
|---|---|---|---|---|
| 662306 | 663290 | ISVa2 | 91.68 | 0 |
| 567760 | 568744 | ISVa2 | 91.68 | 0 |
| 457991 | 458975 | ISVa2 | 91.68 | 0 |
| 1196662 | 1197343 | ISVa2 | 92.52 | 0 |
| 3562836 | 3563273 | ISVa2 | 88.81 | 9.00E-132 |
| 3555761 | 3556059 | ISVa2 | 90.97 | 3.00E-101 |
| 1195094 | 1195400 | ISVa2 | 89.9 | 1.00E-96 |
| 3555188 | 3555325 | ISVa2 | 89.86 | 7.00E-34 |
| 3554035 | 3554129 | ISVa2 | 90.53 | 1.00E-22 |
| 3127540 | 3127677 | ISVa2 | 81.88 | 5.00E-10 |
| 3555477 | 3555608 | ISVa2 | 81.82 | 8.00E-09 |
| 2449063 | 2450305 | ISVpa2 | 88.17 | 0 |
| 1564077 | 1565319 | ISVpa2 | 88.17 | 0 |
| 1593553 | 1594795 | ISVpa2 | 88.17 | 0 |
| 1620799 | 1622041 | ISVpa2 | 88.17 | 0 |
| 1570825 | 1572067 | ISVpa2 | 88.17 | 0 |
| 1562503 | 1563745 | ISVpa2 | 88.17 | 0 |
| 2480679 | 2481921 | ISVpa2 | 88.17 | 0 |
| 1204502 | 1205744 | ISVpa2 | 88.17 | 0 |
| 1201467 | 1202709 | ISVpa2 | 88.17 | 0 |
| 320476 | 321718 | ISVpa2 | 88.17 | 0 |
| 3699282 | 3700283 | ISVal1 | 90.92 | 0 |
| 3561137 | 3562138 | ISVal1 | 90.92 | 0 |
| 3554182 | 3555183 | ISVal1 | 90.92 | 0 |
| 3341671 | 3342672 | ISVal1 | 90.92 | 0 |
| 504763 | 505764 | ISVal1 | 90.92 | 0 |
| 549839 | 550840 | ISVal1 | 90.92 | 0 |
| 672689 | 673690 | ISVal1 | 90.92 | 0 |
| 679750 | 680751 | ISVal1 | 90.92 | 0 |
| 854172 | 855173 | ISVal1 | 90.92 | 0 |
| 865510 | 866511 | ISVal1 | 90.92 | 0 |
| 1234252 | 1235253 | ISVal1 | 90.92 | 0 |
| 2559085 | 2560086 | ISVal1 | 90.92 | 0 |
| 2464308 | 2465309 | ISVal1 | 90.92 | 0 |
| 1568882 | 1569883 | ISVal1 | 90.92 | 0 |
| 1605599 | 1606600 | ISVal1 | 90.92 | 0 |
| 1616556 | 1617557 | ISVal1 | 90.92 | 0 |
| 1990617 | 1991618 | ISVal1 | 90.92 | 0 |
| 2285003 | 2286004 | ISVal1 | 90.92 | 0 |
| 1522240 | 1523241 | ISVal1 | 90.92 | 0 |
| 2506075 | 2507076 | ISVal1 | 90.92 | 0 |
| 1243054 | 1244055 | ISVal1 | 90.92 | 0 |
| 1213077 | 1214078 | ISVal1 | 90.92 | 0 |
| 1202885 | 1203886 | ISVal1 | 90.92 | 0 |
| 2698900 | 2699901 | ISVal1 | 90.92 | 0 |
| 2933655 | 2934656 | ISVal1 | 90.92 | 0 |
| 3090663 | 3091664 | ISVal1 | 90.92 | 0 |
| 662289 | 663290 | ISVal1 | 90.92 | 0 |
| 567743 | 568744 | ISVal1 | 90.92 | 0 |
| 457974 | 458975 | ISVal1 | 90.92 | 0 |
| 1196662 | 1197343 | ISVal1 | 93.55 | 0 |
| 3562836 | 3563273 | ISVal1 | 88.36 | 5.00E-127 |

| | | | | |
|---|---|---|---|---|
| 3555760 | 3556056 | ISVal1 | 87.54 | 3.00E-76 |
| 1195077 | 1195401 | ISVal1 | 85.54 | 4.00E-69 |
| 3555188 | 3555457 | ISVal1 | 83.76 | 3.00E-39 |
| 3560706 | 3560779 | ISVal1 | 86.49 | 1.00E-07 |
| 3555567 | 3555608 | ISVal1 | 92.86 | 3.00E-05 |
| 1564077 | 1565320 | ISVisp3 | 86.66 | 0 |
| 1204501 | 1205744 | ISVisp3 | 86.66 | 0 |
| 1201466 | 1202709 | ISVisp3 | 86.66 | 0 |
| 2449063 | 2450305 | ISVisp3 | 86.65 | 0 |
| 1593553 | 1594795 | ISVisp3 | 86.65 | 0 |
| 1620799 | 1622041 | ISVisp3 | 86.65 | 0 |
| 1570825 | 1572067 | ISVisp3 | 86.65 | 0 |
| 1562503 | 1563745 | ISVisp3 | 86.65 | 0 |
| 2480679 | 2481921 | ISVisp3 | 86.65 | 0 |
| 320476 | 321718 | ISVisp3 | 86.65 | 0 |
| 2449071 | 2450305 | ISVpe1 | 86.15 | 0 |
| 1564077 | 1565311 | ISVpe1 | 86.15 | 0 |
| 1593553 | 1594787 | ISVpe1 | 86.15 | 0 |
| 1620807 | 1622041 | ISVpe1 | 86.15 | 0 |
| 1570833 | 1572067 | ISVpe1 | 86.15 | 0 |
| 1562511 | 1563745 | ISVpe1 | 86.15 | 0 |
| 2480679 | 2481913 | ISVpe1 | 86.15 | 0 |
| 1204510 | 1205744 | ISVpe1 | 86.15 | 0 |
| 1201475 | 1202709 | ISVpe1 | 86.15 | 0 |
| 320484 | 321718 | ISVpe1 | 86.15 | 0 |
| 2449071 | 2450305 | ISVisp5 | 86.15 | 0 |
| 1564077 | 1565311 | ISVisp5 | 86.15 | 0 |
| 1593553 | 1594787 | ISVisp5 | 86.15 | 0 |
| 1620807 | 1622041 | ISVisp5 | 86.15 | 0 |
| 1570833 | 1572067 | ISVisp5 | 86.15 | 0 |
| 1562511 | 1563745 | ISVisp5 | 86.15 | 0 |
| 2480679 | 2481913 | ISVisp5 | 86.15 | 0 |
| 1204510 | 1205744 | ISVisp5 | 86.15 | 0 |
| 1201475 | 1202709 | ISVisp5 | 86.15 | 0 |
| 320484 | 321718 | ISVisp5 | 86.15 | 0 |
| 2213215 | 2214124 | ISVch6 | 88.13 | 0 |
| 2214243 | 2214308 | ISVch6 | 89.39 | 5.00E-10 |
| 3699295 | 3700215 | ISVpa3 | 83.82 | 0 |
| 3561150 | 3562070 | ISVpa3 | 83.82 | 0 |
| 3554195 | 3555115 | ISVpa3 | 83.82 | 0 |
| 3341684 | 3342604 | ISVpa3 | 83.82 | 0 |
| 504831 | 505751 | ISVpa3 | 83.82 | 0 |
| 549907 | 550827 | ISVpa3 | 83.82 | 0 |
| 672757 | 673677 | ISVpa3 | 83.82 | 0 |
| 679818 | 680738 | ISVpa3 | 83.82 | 0 |
| 854240 | 855160 | ISVpa3 | 83.82 | 0 |
| 865578 | 866498 | ISVpa3 | 83.82 | 0 |
| 1234320 | 1235240 | ISVpa3 | 83.82 | 0 |
| 2559098 | 2560018 | ISVpa3 | 83.82 | 0 |
| 2464321 | 2465241 | ISVpa3 | 83.82 | 0 |
| 1568950 | 1569870 | ISVpa3 | 83.82 | 0 |
| 1605667 | 1606587 | ISVpa3 | 83.82 | 0 |

| | | | | |
|---|---|---|---|---|
| 1616624 | 1617544 | ISVpa3 | 83.82 | 0 |
| 1990685 | 1991605 | ISVpa3 | 83.82 | 0 |
| 2285071 | 2285991 | ISVpa3 | 83.82 | 0 |
| 1522253 | 1523173 | ISVpa3 | 83.82 | 0 |
| 2506143 | 2507063 | ISVpa3 | 83.82 | 0 |
| 1243067 | 1243987 | ISVpa3 | 83.82 | 0 |
| 1213090 | 1214010 | ISVpa3 | 83.82 | 0 |
| 1202898 | 1203818 | ISVpa3 | 83.82 | 0 |
| 2698968 | 2699888 | ISVpa3 | 83.82 | 0 |
| 2933723 | 2934643 | ISVpa3 | 83.82 | 0 |
| 3090731 | 3091651 | ISVpa3 | 83.82 | 0 |
| 662302 | 663222 | ISVpa3 | 83.82 | 0 |
| 567756 | 568676 | ISVpa3 | 83.82 | 0 |
| 457987 | 458907 | ISVpa3 | 83.82 | 0 |
| 3562836 | 3563271 | ISVpa3 | 85.78 | 2.00E-99 |
| 1196672 | 1197275 | ISVpa3 | 82.12 | 5.00E-90 |
| 1195090 | 1195397 | ISVpa3 | 87.66 | 2.00E-80 |
| 3555216 | 3555608 | ISVpa3 | 82.11 | 4.00E-57 |
| 3555767 | 3555996 | ISVpa3 | 82.68 | 6.00E-25 |
| 3560618 | 3560773 | ISVpa3 | 82.69 | 5.00E-16 |
| 3554027 | 3554132 | ISVpa3 | 85.85 | 8.00E-15 |
| 3699297 | 3700215 | ISVvu5 | 83.46 | 6.00E-173 |
| 3561152 | 3562070 | ISVvu5 | 83.46 | 6.00E-173 |
| 3554197 | 3555115 | ISVvu5 | 83.46 | 6.00E-173 |
| 3341686 | 3342604 | ISVvu5 | 83.46 | 6.00E-173 |
| 504831 | 505749 | ISVvu5 | 83.46 | 6.00E-173 |
| 549907 | 550825 | ISVvu5 | 83.46 | 6.00E-173 |
| 672757 | 673675 | ISVvu5 | 83.46 | 6.00E-173 |
| 679818 | 680736 | ISVvu5 | 83.46 | 6.00E-173 |
| 854240 | 855158 | ISVvu5 | 83.46 | 6.00E-173 |
| 865578 | 866496 | ISVvu5 | 83.46 | 6.00E-173 |
| 1234320 | 1235238 | ISVvu5 | 83.46 | 6.00E-173 |
| 2559100 | 2560018 | ISVvu5 | 83.46 | 6.00E-173 |
| 2464323 | 2465241 | ISVvu5 | 83.46 | 6.00E-173 |
| 1568950 | 1569868 | ISVvu5 | 83.46 | 6.00E-173 |
| 1605667 | 1606585 | ISVvu5 | 83.46 | 6.00E-173 |
| 1616624 | 1617542 | ISVvu5 | 83.46 | 6.00E-173 |
| 1990685 | 1991603 | ISVvu5 | 83.46 | 6.00E-173 |
| 2285071 | 2285989 | ISVvu5 | 83.46 | 6.00E-173 |
| 1522255 | 1523173 | ISVvu5 | 83.46 | 6.00E-173 |
| 2506143 | 2507061 | ISVvu5 | 83.46 | 6.00E-173 |
| 1243069 | 1243987 | ISVvu5 | 83.46 | 6.00E-173 |
| 1213092 | 1214010 | ISVvu5 | 83.46 | 6.00E-173 |
| 1202900 | 1203818 | ISVvu5 | 83.46 | 6.00E-173 |
| 2698968 | 2699886 | ISVvu5 | 83.46 | 6.00E-173 |
| 2933723 | 2934641 | ISVvu5 | 83.46 | 6.00E-173 |
| 3090731 | 3091649 | ISVvu5 | 83.46 | 6.00E-173 |
| 662304 | 663222 | ISVvu5 | 83.46 | 6.00E-173 |
| 567758 | 568676 | ISVvu5 | 83.46 | 6.00E-173 |
| 457989 | 458907 | ISVvu5 | 83.46 | 6.00E-173 |
| 1196664 | 1197275 | ISVvu5 | 82.35 | 9.00E-95 |
| 3562836 | 3563271 | ISVvu5 | 83.94 | 2.00E-80 |

| | | | | |
|---|---|---|---|---|
| 1195092 | 1195397 | ISVvu5 | 85.95 | 3.00E-67 |
| 3555216 | 3555608 | ISVvu5 | 81.37 | 5.00E-50 |
| 3555767 | 3555996 | ISVvu5 | 82.25 | 1.00E-22 |
| 3560618 | 3560791 | ISVvu5 | 83.33 | 6.00E-22 |
| 3554023 | 3554129 | ISVvu5 | 85.05 | 5.00E-13 |
| 3119603 | 3120135 | ISVa19 | 86.87 | 6.00E-136 |
| 3699304 | 3699860 | ISVbsp3 | 83.12 | 2.00E-95 |
| 3561159 | 3561715 | ISVbsp3 | 83.12 | 2.00E-95 |
| 3554204 | 3554760 | ISVbsp3 | 83.12 | 2.00E-95 |
| 3341693 | 3342249 | ISVbsp3 | 83.12 | 2.00E-95 |
| 505186 | 505742 | ISVbsp3 | 83.12 | 2.00E-95 |
| 550262 | 550818 | ISVbsp3 | 83.12 | 2.00E-95 |
| 673112 | 673668 | ISVbsp3 | 83.12 | 2.00E-95 |
| 680173 | 680729 | ISVbsp3 | 83.12 | 2.00E-95 |
| 854595 | 855151 | ISVbsp3 | 83.12 | 2.00E-95 |
| 865933 | 866489 | ISVbsp3 | 83.12 | 2.00E-95 |
| 1234675 | 1235231 | ISVbsp3 | 83.12 | 2.00E-95 |
| 2559107 | 2559663 | ISVbsp3 | 83.12 | 2.00E-95 |
| 2464330 | 2464886 | ISVbsp3 | 83.12 | 2.00E-95 |
| 1569305 | 1569861 | ISVbsp3 | 83.12 | 2.00E-95 |
| 1606022 | 1606578 | ISVbsp3 | 83.12 | 2.00E-95 |
| 1616979 | 1617535 | ISVbsp3 | 83.12 | 2.00E-95 |
| 1991040 | 1991596 | ISVbsp3 | 83.12 | 2.00E-95 |
| 2285426 | 2285982 | ISVbsp3 | 83.12 | 2.00E-95 |
| 1522262 | 1522818 | ISVbsp3 | 83.12 | 2.00E-95 |
| 2506498 | 2507054 | ISVbsp3 | 83.12 | 2.00E-95 |
| 1243076 | 1243632 | ISVbsp3 | 83.12 | 2.00E-95 |
| 1213099 | 1213655 | ISVbsp3 | 83.12 | 2.00E-95 |
| 1202907 | 1203463 | ISVbsp3 | 83.12 | 2.00E-95 |
| 2699323 | 2699879 | ISVbsp3 | 83.12 | 2.00E-95 |
| 2934078 | 2934634 | ISVbsp3 | 83.12 | 2.00E-95 |
| 3091086 | 3091642 | ISVbsp3 | 83.12 | 2.00E-95 |
| 662311 | 662867 | ISVbsp3 | 83.12 | 2.00E-95 |
| 567765 | 568321 | ISVbsp3 | 83.12 | 2.00E-95 |
| 457996 | 458552 | ISVbsp3 | 83.12 | 2.00E-95 |
| 3562836 | 3563266 | ISVbsp3 | 81.67 | 6.00E-56 |
| 1195099 | 1195394 | ISVbsp3 | 83.11 | 1.00E-44 |
| 1196672 | 1196920 | ISVbsp3 | 84.34 | 8.00E-43 |
| 3555216 | 3555608 | ISVbsp3 | 79.9 | 1.00E-35 |
| 679113 | 679248 | ISVbsp3 | 84.67 | 1.00E-16 |
| 3127471 | 3127677 | ISVbsp3 | 80.68 | 2.00E-15 |
| 3699998 | 3700199 | ISVbsp3 | 80.88 | 2.00E-12 |
| 3561853 | 3562054 | ISVbsp3 | 80.88 | 2.00E-12 |
| 3554898 | 3555099 | ISVbsp3 | 80.88 | 2.00E-12 |
| 3342387 | 3342588 | ISVbsp3 | 80.88 | 2.00E-12 |
| 504847 | 505048 | ISVbsp3 | 80.88 | 2.00E-12 |
| 549923 | 550124 | ISVbsp3 | 80.88 | 2.00E-12 |
| 672773 | 672974 | ISVbsp3 | 80.88 | 2.00E-12 |
| 679834 | 680035 | ISVbsp3 | 80.88 | 2.00E-12 |
| 854256 | 854457 | ISVbsp3 | 80.88 | 2.00E-12 |
| 865594 | 865795 | ISVbsp3 | 80.88 | 2.00E-12 |
| 1234336 | 1234537 | ISVbsp3 | 80.88 | 2.00E-12 |

| | | | | |
|---|---|---|---|---|
| 2559801 | 2560002 | ISVbsp3 | 80.88 | 2.00E-12 |
| 2465024 | 2465225 | ISVbsp3 | 80.88 | 2.00E-12 |
| 1568966 | 1569167 | ISVbsp3 | 80.88 | 2.00E-12 |
| 1605683 | 1605884 | ISVbsp3 | 80.88 | 2.00E-12 |
| 1616640 | 1616841 | ISVbsp3 | 80.88 | 2.00E-12 |
| 1990701 | 1990902 | ISVbsp3 | 80.88 | 2.00E-12 |
| 2285087 | 2285288 | ISVbsp3 | 80.88 | 2.00E-12 |
| 1522956 | 1523157 | ISVbsp3 | 80.88 | 2.00E-12 |
| 2506159 | 2506360 | ISVbsp3 | 80.88 | 2.00E-12 |
| 1243770 | 1243971 | ISVbsp3 | 80.88 | 2.00E-12 |
| 1213793 | 1213994 | ISVbsp3 | 80.88 | 2.00E-12 |
| 1203601 | 1203802 | ISVbsp3 | 80.88 | 2.00E-12 |
| 1197058 | 1197259 | ISVbsp3 | 80.88 | 2.00E-12 |
| 2698984 | 2699185 | ISVbsp3 | 80.88 | 2.00E-12 |
| 2933739 | 2933940 | ISVbsp3 | 80.88 | 2.00E-12 |
| 3090747 | 3090948 | ISVbsp3 | 80.88 | 2.00E-12 |
| 663005 | 663206 | ISVbsp3 | 80.88 | 2.00E-12 |
| 568459 | 568660 | ISVbsp3 | 80.88 | 2.00E-12 |
| 458690 | 458891 | ISVbsp3 | 80.88 | 2.00E-12 |
| 3560655 | 3560773 | ISVbsp3 | 81.51 | 8.00E-06 |
| 3554047 | 3554132 | ISVbsp3 | 83.72 | 3.00E-05 |
| 3560550 | 3560964 | ISV-M52 | 85.58 | 5.00E-90 |
| 3700110 | 3700216 | ISV-M52 | 89.72 | 6.00E-25 |
| 3561965 | 3562071 | ISV-M52 | 89.72 | 6.00E-25 |
| 3555010 | 3555116 | ISV-M52 | 89.72 | 6.00E-25 |
| 3342499 | 3342605 | ISV-M52 | 89.72 | 6.00E-25 |
| 504830 | 504936 | ISV-M52 | 89.72 | 6.00E-25 |
| 549906 | 550012 | ISV-M52 | 89.72 | 6.00E-25 |
| 672756 | 672862 | ISV-M52 | 89.72 | 6.00E-25 |
| 679817 | 679923 | ISV-M52 | 89.72 | 6.00E-25 |
| 854239 | 854345 | ISV-M52 | 89.72 | 6.00E-25 |
| 865577 | 865683 | ISV-M52 | 89.72 | 6.00E-25 |
| 1234319 | 1234425 | ISV-M52 | 89.72 | 6.00E-25 |
| 2559913 | 2560019 | ISV-M52 | 89.72 | 6.00E-25 |
| 2465136 | 2465242 | ISV-M52 | 89.72 | 6.00E-25 |
| 1568949 | 1569055 | ISV-M52 | 89.72 | 6.00E-25 |
| 1605666 | 1605772 | ISV-M52 | 89.72 | 6.00E-25 |
| 1616623 | 1616729 | ISV-M52 | 89.72 | 6.00E-25 |
| 1990684 | 1990790 | ISV-M52 | 89.72 | 6.00E-25 |
| 2285070 | 2285176 | ISV-M52 | 89.72 | 6.00E-25 |
| 1523068 | 1523174 | ISV-M52 | 89.72 | 6.00E-25 |
| 2506142 | 2506248 | ISV-M52 | 89.72 | 6.00E-25 |
| 1243882 | 1243988 | ISV-M52 | 89.72 | 6.00E-25 |
| 1213905 | 1214011 | ISV-M52 | 89.72 | 6.00E-25 |
| 1203713 | 1203819 | ISV-M52 | 89.72 | 6.00E-25 |
| 1197170 | 1197276 | ISV-M52 | 89.72 | 6.00E-25 |
| 2698967 | 2699073 | ISV-M52 | 89.72 | 6.00E-25 |
| 2933722 | 2933828 | ISV-M52 | 89.72 | 6.00E-25 |
| 3090730 | 3090836 | ISV-M52 | 89.72 | 6.00E-25 |
| 663117 | 663223 | ISV-M52 | 89.72 | 6.00E-25 |
| 568571 | 568677 | ISV-M52 | 89.72 | 6.00E-25 |
| 458802 | 458908 | ISV-M52 | 89.72 | 6.00E-25 |

| | | | | |
|---|---|---|---|---|
| 3699304 | 3699509 | ISV-M52 | 82.04 | 1.00E-19 |
| 3561159 | 3561364 | ISV-M52 | 82.04 | 1.00E-19 |
| 3554204 | 3554409 | ISV-M52 | 82.04 | 1.00E-19 |
| 3341693 | 3341898 | ISV-M52 | 82.04 | 1.00E-19 |
| 505537 | 505742 | ISV-M52 | 82.04 | 1.00E-19 |
| 550613 | 550818 | ISV-M52 | 82.04 | 1.00E-19 |
| 673463 | 673668 | ISV-M52 | 82.04 | 1.00E-19 |
| 680524 | 680729 | ISV-M52 | 82.04 | 1.00E-19 |
| 854946 | 855151 | ISV-M52 | 82.04 | 1.00E-19 |
| 866284 | 866489 | ISV-M52 | 82.04 | 1.00E-19 |
| 1235026 | 1235231 | ISV-M52 | 82.04 | 1.00E-19 |
| 2559107 | 2559312 | ISV-M52 | 82.04 | 1.00E-19 |
| 2464330 | 2464535 | ISV-M52 | 82.04 | 1.00E-19 |
| 1569656 | 1569861 | ISV-M52 | 82.04 | 1.00E-19 |
| 1606373 | 1606578 | ISV-M52 | 82.04 | 1.00E-19 |
| 1617330 | 1617535 | ISV-M52 | 82.04 | 1.00E-19 |
| 1991391 | 1991596 | ISV-M52 | 82.04 | 1.00E-19 |
| 2285777 | 2285982 | ISV-M52 | 82.04 | 1.00E-19 |
| 1522262 | 1522467 | ISV-M52 | 82.04 | 1.00E-19 |
| 2506849 | 2507054 | ISV-M52 | 82.04 | 1.00E-19 |
| 1243076 | 1243281 | ISV-M52 | 82.04 | 1.00E-19 |
| 1213099 | 1213304 | ISV-M52 | 82.04 | 1.00E-19 |
| 1202907 | 1203112 | ISV-M52 | 82.04 | 1.00E-19 |
| 1195099 | 1195304 | ISV-M52 | 82.04 | 1.00E-19 |
| 2699674 | 2699879 | ISV-M52 | 82.04 | 1.00E-19 |
| 2934429 | 2934634 | ISV-M52 | 82.04 | 1.00E-19 |
| 3091437 | 3091642 | ISV-M52 | 82.04 | 1.00E-19 |
| 662311 | 662516 | ISV-M52 | 82.04 | 1.00E-19 |
| 567765 | 567970 | ISV-M52 | 82.04 | 1.00E-19 |
| 457996 | 458201 | ISV-M52 | 82.04 | 1.00E-19 |
| 3555871 | 3556001 | ISV-M52 | 83.97 | 2.00E-15 |
| 1196676 | 1196953 | ISV-M52 | 78.78 | 2.00E-12 |
| 3562881 | 3562966 | ISV-M52 | 86.05 | 5.00E-10 |
| 3563061 | 3563266 | ISV-M52 | 80.1 | 5.00E-10 |
| 3560550 | 3560964 | ISVch8 | 84.86 | 8.00E-83 |
| 3699304 | 3700283 | ISVch8 | 78.08 | 2.00E-55 |
| 3561159 | 3562138 | ISVch8 | 78.08 | 2.00E-55 |
| 3554204 | 3555183 | ISVch8 | 78.08 | 2.00E-55 |
| 3341693 | 3342672 | ISVch8 | 78.08 | 2.00E-55 |
| 504763 | 505742 | ISVch8 | 78.08 | 2.00E-55 |
| 549839 | 550818 | ISVch8 | 78.08 | 2.00E-55 |
| 672689 | 673668 | ISVch8 | 78.08 | 2.00E-55 |
| 679750 | 680729 | ISVch8 | 78.08 | 2.00E-55 |
| 854172 | 855151 | ISVch8 | 78.08 | 2.00E-55 |
| 865510 | 866489 | ISVch8 | 78.08 | 2.00E-55 |
| 1234252 | 1235231 | ISVch8 | 78.08 | 2.00E-55 |
| 2559107 | 2560086 | ISVch8 | 78.08 | 2.00E-55 |
| 2464330 | 2465309 | ISVch8 | 78.08 | 2.00E-55 |
| 1568882 | 1569861 | ISVch8 | 78.08 | 2.00E-55 |
| 1605599 | 1606578 | ISVch8 | 78.08 | 2.00E-55 |
| 1616556 | 1617535 | ISVch8 | 78.08 | 2.00E-55 |
| 1990617 | 1991596 | ISVch8 | 78.08 | 2.00E-55 |

| | | | | |
|---|---|---|---|---|
| 2285003 | 2285982 | ISVch8 | 78.08 | 2.00E-55 |
| 1522262 | 1523241 | ISVch8 | 78.08 | 2.00E-55 |
| 2506075 | 2507054 | ISVch8 | 78.08 | 2.00E-55 |
| 1243076 | 1244055 | ISVch8 | 78.08 | 2.00E-55 |
| 1213099 | 1214078 | ISVch8 | 78.08 | 2.00E-55 |
| 1202907 | 1203886 | ISVch8 | 78.08 | 2.00E-55 |
| 2698900 | 2699879 | ISVch8 | 78.08 | 2.00E-55 |
| 2933655 | 2934634 | ISVch8 | 78.08 | 2.00E-55 |
| 3090663 | 3091642 | ISVch8 | 78.08 | 2.00E-55 |
| 662311 | 663290 | ISVch8 | 78.08 | 2.00E-55 |
| 567765 | 568744 | ISVch8 | 78.08 | 2.00E-55 |
| 457996 | 458975 | ISVch8 | 78.08 | 2.00E-55 |
| 1196664 | 1197343 | ISVch8 | 78.12 | 7.00E-34 |
| 3562848 | 3563266 | ISVch8 | 79 | 2.00E-27 |
| 1195099 | 1195369 | ISVch8 | 80.07 | 4.00E-20 |
| 3555896 | 3556059 | ISVch8 | 84.34 | 2.00E-15 |
| 3562466 | 3562794 | ISVvu1 | 84.8 | 2.00E-64 |
| 3553897 | 3553958 | ISVvu1 | 91.94 | 2.00E-12 |
| 2213946 | 2214308 | ISVa15 | 83.47 | 1.00E-60 |
| 3538196 | 3538363 | ISVa15 | 81.66 | 8.00E-12 |
| 2213375 | 2213425 | ISVa15 | 90.2 | 8.00E-06 |
| 670726 | 671418 | ISVfl1 | 78.98 | 2.00E-55 |
| 670437 | 670608 | ISVfl1 | 83.24 | 6.00E-19 |
| 1626236 | 1626471 | ISShwo3 | 85.59 | 5.00E-47 |
| 3550905 | 3551112 | ISVa1 | 87.5 | 5.00E-47 |
| 3551296 | 3551492 | ISVa1 | 86.8 | 8.00E-43 |
| 3551668 | 3551818 | ISVa1 | 84.87 | 9.00E-21 |
| 1626238 | 1626471 | ISPtu2 | 86.02 | 1.00E-44 |
| 1626236 | 1626471 | ISSpi5 | 85.23 | 8.00E-43 |
| 1625353 | 1625442 | ISSpi5 | 84.44 | 1.00E-07 |
| 3699805 | 3700208 | ISVba2 | 79.95 | 4.00E-35 |
| 3561660 | 3562063 | ISVba2 | 79.95 | 4.00E-35 |
| 3554705 | 3555108 | ISVba2 | 79.95 | 4.00E-35 |
| 3342194 | 3342597 | ISVba2 | 79.95 | 4.00E-35 |
| 504838 | 505241 | ISVba2 | 79.95 | 4.00E-35 |
| 549914 | 550317 | ISVba2 | 79.95 | 4.00E-35 |
| 672764 | 673167 | ISVba2 | 79.95 | 4.00E-35 |
| 679825 | 680228 | ISVba2 | 79.95 | 4.00E-35 |
| 854247 | 854650 | ISVba2 | 79.95 | 4.00E-35 |
| 865585 | 865988 | ISVba2 | 79.95 | 4.00E-35 |
| 1234327 | 1234730 | ISVba2 | 79.95 | 4.00E-35 |
| 2559608 | 2560011 | ISVba2 | 79.95 | 4.00E-35 |
| 2464831 | 2465234 | ISVba2 | 79.95 | 4.00E-35 |
| 1568957 | 1569360 | ISVba2 | 79.95 | 4.00E-35 |
| 1605674 | 1606077 | ISVba2 | 79.95 | 4.00E-35 |
| 1616631 | 1617034 | ISVba2 | 79.95 | 4.00E-35 |
| 1990692 | 1991095 | ISVba2 | 79.95 | 4.00E-35 |
| 2285078 | 2285481 | ISVba2 | 79.95 | 4.00E-35 |
| 1522763 | 1523166 | ISVba2 | 79.95 | 4.00E-35 |
| 2506150 | 2506553 | ISVba2 | 79.95 | 4.00E-35 |
| 1243577 | 1243980 | ISVba2 | 79.95 | 4.00E-35 |
| 1213600 | 1214003 | ISVba2 | 79.95 | 4.00E-35 |

| | | | | |
|---|---|---|---|---|
| 1203408 | 1203811 | ISVba2 | 79.95 | 4.00E-35 |
| 1196865 | 1197268 | ISVba2 | 79.95 | 4.00E-35 |
| 2698975 | 2699378 | ISVba2 | 79.95 | 4.00E-35 |
| 2933730 | 2934133 | ISVba2 | 79.95 | 4.00E-35 |
| 3090738 | 3091141 | ISVba2 | 79.95 | 4.00E-35 |
| 662812 | 663215 | ISVba2 | 79.95 | 4.00E-35 |
| 568266 | 568669 | ISVba2 | 79.95 | 4.00E-35 |
| 458497 | 458900 | ISVba2 | 79.95 | 4.00E-35 |
| 3560608 | 3560859 | ISVba2 | 81.35 | 2.00E-25 |
| 3555896 | 3556052 | ISVba2 | 86.08 | 2.00E-24 |
| 1626189 | 1626468 | IS492 | 83.39 | 4.00E-35 |
| 1625353 | 1625532 | IS492 | 81.67 | 5.00E-16 |
| 678825 | 679026 | ISShes15 | 84.24 | 2.00E-27 |
| 693859 | 693964 | ISShfr4 | 89.72 | 1.00E-22 |
| 678089 | 678233 | ISSod13 | 84.83 | 2.00E-21 |
| 678801 | 678934 | ISSod13 | 84.33 | 3.00E-17 |
| 677831 | 677868 | ISSod13 | 94.74 | 3.00E-05 |
| 693860 | 694041 | ISSpe3 | 84.24 | 9.00E-21 |
| 3700099 | 3700202 | ISSpu11 | 87.5 | 2.00E-18 |
| 3561954 | 3562057 | ISSpu11 | 87.5 | 2.00E-18 |
| 3554999 | 3555102 | ISSpu11 | 87.5 | 2.00E-18 |
| 3342488 | 3342591 | ISSpu11 | 87.5 | 2.00E-18 |
| 504844 | 504947 | ISSpu11 | 87.5 | 2.00E-18 |
| 549920 | 550023 | ISSpu11 | 87.5 | 2.00E-18 |
| 672770 | 672873 | ISSpu11 | 87.5 | 2.00E-18 |
| 679831 | 679934 | ISSpu11 | 87.5 | 2.00E-18 |
| 854253 | 854356 | ISSpu11 | 87.5 | 2.00E-18 |
| 865591 | 865694 | ISSpu11 | 87.5 | 2.00E-18 |
| 1234333 | 1234436 | ISSpu11 | 87.5 | 2.00E-18 |
| 2559902 | 2560005 | ISSpu11 | 87.5 | 2.00E-18 |
| 2465125 | 2465228 | ISSpu11 | 87.5 | 2.00E-18 |
| 1568963 | 1569066 | ISSpu11 | 87.5 | 2.00E-18 |
| 1605680 | 1605783 | ISSpu11 | 87.5 | 2.00E-18 |
| 1616637 | 1616740 | ISSpu11 | 87.5 | 2.00E-18 |
| 1990698 | 1990801 | ISSpu11 | 87.5 | 2.00E-18 |
| 2285084 | 2285187 | ISSpu11 | 87.5 | 2.00E-18 |
| 1523057 | 1523160 | ISSpu11 | 87.5 | 2.00E-18 |
| 2506156 | 2506259 | ISSpu11 | 87.5 | 2.00E-18 |
| 1243871 | 1243974 | ISSpu11 | 87.5 | 2.00E-18 |
| 1213894 | 1213997 | ISSpu11 | 87.5 | 2.00E-18 |
| 1203702 | 1203805 | ISSpu11 | 87.5 | 2.00E-18 |
| 1197159 | 1197262 | ISSpu11 | 87.5 | 2.00E-18 |
| 2698981 | 2699084 | ISSpu11 | 87.5 | 2.00E-18 |
| 2933736 | 2933839 | ISSpu11 | 87.5 | 2.00E-18 |
| 3090744 | 3090847 | ISSpu11 | 87.5 | 2.00E-18 |
| 663106 | 663209 | ISSpu11 | 87.5 | 2.00E-18 |
| 568560 | 568663 | ISSpu11 | 87.5 | 2.00E-18 |
| 458791 | 458894 | ISSpu11 | 87.5 | 2.00E-18 |
| 3699996 | 3700062 | ISSpu11 | 89.55 | 1.00E-10 |
| 3561851 | 3561917 | ISSpu11 | 89.55 | 1.00E-10 |
| 3554896 | 3554962 | ISSpu11 | 89.55 | 1.00E-10 |
| 3342385 | 3342451 | ISSpu11 | 89.55 | 1.00E-10 |

| | | | | |
|---|---|---|---|---|
| 504984 | 505050 | ISSpu11 | 89.55 | 1.00E-10 |
| 550060 | 550126 | ISSpu11 | 89.55 | 1.00E-10 |
| 672910 | 672976 | ISSpu11 | 89.55 | 1.00E-10 |
| 679971 | 680037 | ISSpu11 | 89.55 | 1.00E-10 |
| 854393 | 854459 | ISSpu11 | 89.55 | 1.00E-10 |
| 865731 | 865797 | ISSpu11 | 89.55 | 1.00E-10 |
| 1234473 | 1234539 | ISSpu11 | 89.55 | 1.00E-10 |
| 2559799 | 2559865 | ISSpu11 | 89.55 | 1.00E-10 |
| 2465022 | 2465088 | ISSpu11 | 89.55 | 1.00E-10 |
| 1569103 | 1569169 | ISSpu11 | 89.55 | 1.00E-10 |
| 1605820 | 1605886 | ISSpu11 | 89.55 | 1.00E-10 |
| 1616777 | 1616843 | ISSpu11 | 89.55 | 1.00E-10 |
| 1990838 | 1990904 | ISSpu11 | 89.55 | 1.00E-10 |
| 2285224 | 2285290 | ISSpu11 | 89.55 | 1.00E-10 |
| 1522954 | 1523020 | ISSpu11 | 89.55 | 1.00E-10 |
| 2506296 | 2506362 | ISSpu11 | 89.55 | 1.00E-10 |
| 1243768 | 1243834 | ISSpu11 | 89.55 | 1.00E-10 |
| 1213791 | 1213857 | ISSpu11 | 89.55 | 1.00E-10 |
| 1203599 | 1203665 | ISSpu11 | 89.55 | 1.00E-10 |
| 1197056 | 1197122 | ISSpu11 | 89.55 | 1.00E-10 |
| 2699121 | 2699187 | ISSpu11 | 89.55 | 1.00E-10 |
| 2933876 | 2933942 | ISSpu11 | 89.55 | 1.00E-10 |
| 3090884 | 3090950 | ISSpu11 | 89.55 | 1.00E-10 |
| 663003 | 663069 | ISSpu11 | 89.55 | 1.00E-10 |
| 568457 | 568523 | ISSpu11 | 89.55 | 1.00E-10 |
| 458688 | 458754 | ISSpu11 | 89.55 | 1.00E-10 |
| 3555883 | 3555987 | ISSpu11 | 83.81 | 2.00E-09 |
| 3699304 | 3699572 | ISSpu11 | 78.44 | 2.00E-09 |
| 3561159 | 3561427 | ISSpu11 | 78.44 | 2.00E-09 |
| 3554204 | 3554472 | ISSpu11 | 78.44 | 2.00E-09 |
| 3341693 | 3341961 | ISSpu11 | 78.44 | 2.00E-09 |
| 505474 | 505742 | ISSpu11 | 78.44 | 2.00E-09 |
| 550550 | 550818 | ISSpu11 | 78.44 | 2.00E-09 |
| 673400 | 673668 | ISSpu11 | 78.44 | 2.00E-09 |
| 680461 | 680729 | ISSpu11 | 78.44 | 2.00E-09 |
| 854883 | 855151 | ISSpu11 | 78.44 | 2.00E-09 |
| 866221 | 866489 | ISSpu11 | 78.44 | 2.00E-09 |
| 1234963 | 1235231 | ISSpu11 | 78.44 | 2.00E-09 |
| 2559107 | 2559375 | ISSpu11 | 78.44 | 2.00E-09 |
| 2464330 | 2464598 | ISSpu11 | 78.44 | 2.00E-09 |
| 1569593 | 1569861 | ISSpu11 | 78.44 | 2.00E-09 |
| 1606310 | 1606578 | ISSpu11 | 78.44 | 2.00E-09 |
| 1617267 | 1617535 | ISSpu11 | 78.44 | 2.00E-09 |
| 1991328 | 1991596 | ISSpu11 | 78.44 | 2.00E-09 |
| 2285714 | 2285982 | ISSpu11 | 78.44 | 2.00E-09 |
| 1522262 | 1522530 | ISSpu11 | 78.44 | 2.00E-09 |
| 2506786 | 2507054 | ISSpu11 | 78.44 | 2.00E-09 |
| 1243076 | 1243344 | ISSpu11 | 78.44 | 2.00E-09 |
| 1213099 | 1213367 | ISSpu11 | 78.44 | 2.00E-09 |
| 1202907 | 1203175 | ISSpu11 | 78.44 | 2.00E-09 |
| 1195099 | 1195367 | ISSpu11 | 78.44 | 2.00E-09 |
| 2699611 | 2699879 | ISSpu11 | 78.44 | 2.00E-09 |

| | | | | |
|---|---|---|---|---|
| 2934366 | 2934634 | ISSpu11 | 78.44 | 2.00E-09 |
| 3091374 | 3091642 | ISSpu11 | 78.44 | 2.00E-09 |
| 662311 | 662579 | ISSpu11 | 78.44 | 2.00E-09 |
| 567765 | 568033 | ISSpu11 | 78.44 | 2.00E-09 |
| 457996 | 458264 | ISSpu11 | 78.44 | 2.00E-09 |
| 3699745 | 3699836 | ISSpu11 | 84.78 | 8.00E-09 |
| 3561600 | 3561691 | ISSpu11 | 84.78 | 8.00E-09 |
| 3554645 | 3554736 | ISSpu11 | 84.78 | 8.00E-09 |
| 3342134 | 3342225 | ISSpu11 | 84.78 | 8.00E-09 |
| 505210 | 505301 | ISSpu11 | 84.78 | 8.00E-09 |
| 550286 | 550377 | ISSpu11 | 84.78 | 8.00E-09 |
| 673136 | 673227 | ISSpu11 | 84.78 | 8.00E-09 |
| 680197 | 680288 | ISSpu11 | 84.78 | 8.00E-09 |
| 854619 | 854710 | ISSpu11 | 84.78 | 8.00E-09 |
| 865957 | 866048 | ISSpu11 | 84.78 | 8.00E-09 |
| 1234699 | 1234790 | ISSpu11 | 84.78 | 8.00E-09 |
| 2559548 | 2559639 | ISSpu11 | 84.78 | 8.00E-09 |
| 2464771 | 2464862 | ISSpu11 | 84.78 | 8.00E-09 |
| 1569329 | 1569420 | ISSpu11 | 84.78 | 8.00E-09 |
| 1606046 | 1606137 | ISSpu11 | 84.78 | 8.00E-09 |
| 1617003 | 1617094 | ISSpu11 | 84.78 | 8.00E-09 |
| 1991064 | 1991155 | ISSpu11 | 84.78 | 8.00E-09 |
| 2285450 | 2285541 | ISSpu11 | 84.78 | 8.00E-09 |
| 1522703 | 1522794 | ISSpu11 | 84.78 | 8.00E-09 |
| 2506522 | 2506613 | ISSpu11 | 84.78 | 8.00E-09 |
| 1243517 | 1243608 | ISSpu11 | 84.78 | 8.00E-09 |
| 1213540 | 1213631 | ISSpu11 | 84.78 | 8.00E-09 |
| 1203348 | 1203439 | ISSpu11 | 84.78 | 8.00E-09 |
| 1196805 | 1196896 | ISSpu11 | 84.78 | 8.00E-09 |
| 2699347 | 2699438 | ISSpu11 | 84.78 | 8.00E-09 |
| 2934102 | 2934193 | ISSpu11 | 84.78 | 8.00E-09 |
| 3091110 | 3091201 | ISSpu11 | 84.78 | 8.00E-09 |
| 662752 | 662843 | ISSpu11 | 84.78 | 8.00E-09 |
| 568206 | 568297 | ISSpu11 | 84.78 | 8.00E-09 |
| 458437 | 458528 | ISSpu11 | 84.78 | 8.00E-09 |
| 3555767 | 3555836 | ISSpu11 | 87.14 | 1.00E-07 |
| 529035 | 529112 | ISEc52 | 91.03 | 3.00E-17 |
| 1601998 | 1602075 | ISEc52 | 91.03 | 3.00E-17 |
| 1553751 | 1553828 | ISEc52 | 91.03 | 3.00E-17 |
| 1196264 | 1196341 | ISEc52 | 91.03 | 3.00E-17 |
| 529050 | 529119 | ISSlo2 | 92.86 | 3.00E-17 |
| 1601991 | 1602060 | ISSlo2 | 92.86 | 3.00E-17 |
| 1553744 | 1553813 | ISSlo2 | 92.86 | 3.00E-17 |
| 1196257 | 1196326 | ISSlo2 | 92.86 | 3.00E-17 |
| 1625352 | 1625696 | ISSde13 | 79.19 | 3.00E-17 |
| 1625845 | 1626054 | ISSde13 | 79.05 | 1.00E-07 |
| 677831 | 678027 | ISSde2 | 81.73 | 3.00E-17 |
| 678860 | 678938 | ISSde2 | 89.87 | 2.00E-15 |
| 3133129 | 3133186 | ISVvu4 | 96.55 | 3.00E-17 |
| 678075 | 678165 | ISVvu4 | 86.81 | 5.00E-13 |
| 678885 | 678937 | ISVvu4 | 92.45 | 2.00E-09 |
| 529048 | 529112 | IS911 | 93.85 | 1.00E-16 |

| | | | | | |
|---|---|---|---|---|---|
| | 1601998 | 1602062 | IS911 | 93.85 | 1.00E-16 |
| | 1553751 | 1553815 | IS911 | 93.85 | 1.00E-16 |
| | 1196264 | 1196328 | IS911 | 93.85 | 1.00E-16 |
| | 678077 | 678204 | ISErsp1 | 84.38 | 5.00E-16 |
| | 678872 | 678934 | ISErsp1 | 87.3 | 8.00E-06 |
| | 678801 | 678934 | ISSma11 | 83.58 | 8.00E-15 |
| | 678044 | 678216 | ISSma11 | 80.35 | 2.00E-09 |
| | 529014 | 529119 | ISCfr25 | 85.85 | 8.00E-15 |
| | 1601991 | 1602096 | ISCfr25 | 85.85 | 8.00E-15 |
| | 1553744 | 1553849 | ISCfr25 | 85.85 | 8.00E-15 |
| | 1196257 | 1196362 | ISCfr25 | 85.85 | 8.00E-15 |
| | 529436 | 529494 | ISCfr25 | 89.83 | 3.00E-08 |
| | 1601616 | 1601674 | ISCfr25 | 89.83 | 3.00E-08 |
| | 1553369 | 1553427 | ISCfr25 | 89.83 | 3.00E-08 |
| | 1195882 | 1195940 | ISCfr25 | 89.83 | 3.00E-08 |
| | 528713 | 528746 | ISCfr25 | 97.06 | 3.00E-05 |
| | 1602364 | 1602397 | ISCfr25 | 97.06 | 3.00E-05 |
| | 1554117 | 1554150 | ISCfr25 | 97.06 | 3.00E-05 |
| | 1196630 | 1196663 | ISCfr25 | 97.06 | 3.00E-05 |
| | 529047 | 529112 | ISLad1 | 92.42 | 8.00E-15 |
| | 1601998 | 1602063 | ISLad1 | 92.42 | 8.00E-15 |
| | 1553751 | 1553816 | ISLad1 | 92.42 | 8.00E-15 |
| | 1196264 | 1196329 | ISLad1 | 92.42 | 8.00E-15 |
| | 3700111 | 3700187 | ISSpu20 | 89.61 | 3.00E-14 |
| | 3561966 | 3562042 | ISSpu20 | 89.61 | 3.00E-14 |
| N8T11 | 3555011 | 3555087 | ISSpu20 | 89.61 | 3.00E-14 |
| | 3342500 | 3342576 | ISSpu20 | 89.61 | 3.00E-14 |
| | 504859 | 504935 | ISSpu20 | 89.61 | 3.00E-14 |
| | 549935 | 550011 | ISSpu20 | 89.61 | 3.00E-14 |
| | 672785 | 672861 | ISSpu20 | 89.61 | 3.00E-14 |
| | 679846 | 679922 | ISSpu20 | 89.61 | 3.00E-14 |
| | 854268 | 854344 | ISSpu20 | 89.61 | 3.00E-14 |
| | 865606 | 865682 | ISSpu20 | 89.61 | 3.00E-14 |
| | 1234348 | 1234424 | ISSpu20 | 89.61 | 3.00E-14 |
| | 2559914 | 2559990 | ISSpu20 | 89.61 | 3.00E-14 |
| | 2465137 | 2465213 | ISSpu20 | 89.61 | 3.00E-14 |
| | 1568978 | 1569054 | ISSpu20 | 89.61 | 3.00E-14 |
| | 1605695 | 1605771 | ISSpu20 | 89.61 | 3.00E-14 |
| | 1616652 | 1616728 | ISSpu20 | 89.61 | 3.00E-14 |
| | 1990713 | 1990789 | ISSpu20 | 89.61 | 3.00E-14 |
| | 2285099 | 2285175 | ISSpu20 | 89.61 | 3.00E-14 |
| | 1523069 | 1523145 | ISSpu20 | 89.61 | 3.00E-14 |
| | 2506171 | 2506247 | ISSpu20 | 89.61 | 3.00E-14 |
| | 1243883 | 1243959 | ISSpu20 | 89.61 | 3.00E-14 |
| | 1213906 | 1213982 | ISSpu20 | 89.61 | 3.00E-14 |
| | 1203714 | 1203790 | ISSpu20 | 89.61 | 3.00E-14 |
| | 1197171 | 1197247 | ISSpu20 | 89.61 | 3.00E-14 |
| | 2698996 | 2699072 | ISSpu20 | 89.61 | 3.00E-14 |
| | 2933751 | 2933827 | ISSpu20 | 89.61 | 3.00E-14 |
| | 3090759 | 3090835 | ISSpu20 | 89.61 | 3.00E-14 |
| | 663118 | 663194 | ISSpu20 | 89.61 | 3.00E-14 |
| | 568572 | 568648 | ISSpu20 | 89.61 | 3.00E-14 |

| | | | | |
|---|---|---|---|---|
| 458803 | 458879 | ISSpu20 | 89.61 | 3.00E-14 |
| 3555893 | 3555972 | ISSpu20 | 85 | 2.00E-06 |
| 529041 | 529113 | ISSpu4 | 90.41 | 3.00E-14 |
| 1601997 | 1602069 | ISSpu4 | 90.41 | 3.00E-14 |
| 1553750 | 1553822 | ISSpu4 | 90.41 | 3.00E-14 |
| 1196263 | 1196335 | ISSpu4 | 90.41 | 3.00E-14 |
| 529035 | 529112 | ISBrsa1 | 88.46 | 2.00E-12 |
| 1601998 | 1602075 | ISBrsa1 | 88.46 | 2.00E-12 |
| 1553751 | 1553828 | ISBrsa1 | 88.46 | 2.00E-12 |
| 1196264 | 1196341 | ISBrsa1 | 88.46 | 2.00E-12 |
| 677831 | 677947 | ISAlw35 | 85.47 | 8.00E-12 |
| 529041 | 529119 | ISVch4 | 87.34 | 1.00E-10 |
| 1601991 | 1602069 | ISVch4 | 87.34 | 1.00E-10 |
| 1553744 | 1553822 | ISVch4 | 87.34 | 1.00E-10 |
| 1196257 | 1196335 | ISVch4 | 87.34 | 1.00E-10 |
| 3700117 | 3700199 | ISSod12 | 86.75 | 1.00E-10 |
| 3561972 | 3562054 | ISSod12 | 86.75 | 1.00E-10 |
| 3555902 | 3555984 | ISSod12 | 86.75 | 1.00E-10 |
| 3555017 | 3555099 | ISSod12 | 86.75 | 1.00E-10 |
| 3342506 | 3342588 | ISSod12 | 86.75 | 1.00E-10 |
| 504847 | 504929 | ISSod12 | 86.75 | 1.00E-10 |
| 549923 | 550005 | ISSod12 | 86.75 | 1.00E-10 |
| 672773 | 672855 | ISSod12 | 86.75 | 1.00E-10 |
| 679834 | 679916 | ISSod12 | 86.75 | 1.00E-10 |
| 854256 | 854338 | ISSod12 | 86.75 | 1.00E-10 |
| 865594 | 865676 | ISSod12 | 86.75 | 1.00E-10 |
| 1234336 | 1234418 | ISSod12 | 86.75 | 1.00E-10 |
| 2559920 | 2560002 | ISSod12 | 86.75 | 1.00E-10 |
| 2465143 | 2465225 | ISSod12 | 86.75 | 1.00E-10 |
| 1568966 | 1569048 | ISSod12 | 86.75 | 1.00E-10 |
| 1605683 | 1605765 | ISSod12 | 86.75 | 1.00E-10 |
| 1616640 | 1616722 | ISSod12 | 86.75 | 1.00E-10 |
| 1990701 | 1990783 | ISSod12 | 86.75 | 1.00E-10 |
| 2285087 | 2285169 | ISSod12 | 86.75 | 1.00E-10 |
| 1523075 | 1523157 | ISSod12 | 86.75 | 1.00E-10 |
| 2506159 | 2506241 | ISSod12 | 86.75 | 1.00E-10 |
| 1243889 | 1243971 | ISSod12 | 86.75 | 1.00E-10 |
| 1213912 | 1213994 | ISSod12 | 86.75 | 1.00E-10 |
| 1203720 | 1203802 | ISSod12 | 86.75 | 1.00E-10 |
| 1197177 | 1197259 | ISSod12 | 86.75 | 1.00E-10 |
| 2698984 | 2699066 | ISSod12 | 86.75 | 1.00E-10 |
| 2933739 | 2933821 | ISSod12 | 86.75 | 1.00E-10 |
| 3090747 | 3090829 | ISSod12 | 86.75 | 1.00E-10 |
| 663124 | 663206 | ISSod12 | 86.75 | 1.00E-10 |
| 568578 | 568660 | ISSod12 | 86.75 | 1.00E-10 |
| 458809 | 458891 | ISSod12 | 86.75 | 1.00E-10 |
| 3699760 | 3699836 | ISSod12 | 85.71 | 5.00E-07 |
| 3561615 | 3561691 | ISSod12 | 85.71 | 5.00E-07 |
| 3554660 | 3554736 | ISSod12 | 85.71 | 5.00E-07 |
| 3342149 | 3342225 | ISSod12 | 85.71 | 5.00E-07 |
| 505210 | 505286 | ISSod12 | 85.71 | 5.00E-07 |
| 550286 | 550362 | ISSod12 | 85.71 | 5.00E-07 |

| | | | | |
|---|---|---|---|---|
| 673136 | 673212 | ISSod12 | 85.71 | 5.00E-07 |
| 680197 | 680273 | ISSod12 | 85.71 | 5.00E-07 |
| 854619 | 854695 | ISSod12 | 85.71 | 5.00E-07 |
| 865957 | 866033 | ISSod12 | 85.71 | 5.00E-07 |
| 1234699 | 1234775 | ISSod12 | 85.71 | 5.00E-07 |
| 2559563 | 2559639 | ISSod12 | 85.71 | 5.00E-07 |
| 2464786 | 2464862 | ISSod12 | 85.71 | 5.00E-07 |
| 1569329 | 1569405 | ISSod12 | 85.71 | 5.00E-07 |
| 1606046 | 1606122 | ISSod12 | 85.71 | 5.00E-07 |
| 1617003 | 1617079 | ISSod12 | 85.71 | 5.00E-07 |
| 1991064 | 1991140 | ISSod12 | 85.71 | 5.00E-07 |
| 2285450 | 2285526 | ISSod12 | 85.71 | 5.00E-07 |
| 1522718 | 1522794 | ISSod12 | 85.71 | 5.00E-07 |
| 2506522 | 2506598 | ISSod12 | 85.71 | 5.00E-07 |
| 1243532 | 1243608 | ISSod12 | 85.71 | 5.00E-07 |
| 1213555 | 1213631 | ISSod12 | 85.71 | 5.00E-07 |
| 1203363 | 1203439 | ISSod12 | 85.71 | 5.00E-07 |
| 1196820 | 1196896 | ISSod12 | 85.71 | 5.00E-07 |
| 2699347 | 2699423 | ISSod12 | 85.71 | 5.00E-07 |
| 2934102 | 2934178 | ISSod12 | 85.71 | 5.00E-07 |
| 3091110 | 3091186 | ISSod12 | 85.71 | 5.00E-07 |
| 662767 | 662843 | ISSod12 | 85.71 | 5.00E-07 |
| 568221 | 568297 | ISSod12 | 85.71 | 5.00E-07 |
| 458452 | 458528 | ISSod12 | 85.71 | 5.00E-07 |
| 3699304 | 3699542 | ISSod12 | 78.24 | 8.00E-06 |
| 3561159 | 3561397 | ISSod12 | 78.24 | 8.00E-06 |
| 3554204 | 3554442 | ISSod12 | 78.24 | 8.00E-06 |
| 3341693 | 3341931 | ISSod12 | 78.24 | 8.00E-06 |
| 505504 | 505742 | ISSod12 | 78.24 | 8.00E-06 |
| 550580 | 550818 | ISSod12 | 78.24 | 8.00E-06 |
| 673430 | 673668 | ISSod12 | 78.24 | 8.00E-06 |
| 680491 | 680729 | ISSod12 | 78.24 | 8.00E-06 |
| 854913 | 855151 | ISSod12 | 78.24 | 8.00E-06 |
| 866251 | 866489 | ISSod12 | 78.24 | 8.00E-06 |
| 1234993 | 1235231 | ISSod12 | 78.24 | 8.00E-06 |
| 2559107 | 2559345 | ISSod12 | 78.24 | 8.00E-06 |
| 2464330 | 2464568 | ISSod12 | 78.24 | 8.00E-06 |
| 1569623 | 1569861 | ISSod12 | 78.24 | 8.00E-06 |
| 1606340 | 1606578 | ISSod12 | 78.24 | 8.00E-06 |
| 1617297 | 1617535 | ISSod12 | 78.24 | 8.00E-06 |
| 1991358 | 1991596 | ISSod12 | 78.24 | 8.00E-06 |
| 2285744 | 2285982 | ISSod12 | 78.24 | 8.00E-06 |
| 1522262 | 1522500 | ISSod12 | 78.24 | 8.00E-06 |
| 2506816 | 2507054 | ISSod12 | 78.24 | 8.00E-06 |
| 1243076 | 1243314 | ISSod12 | 78.24 | 8.00E-06 |
| 1213099 | 1213337 | ISSod12 | 78.24 | 8.00E-06 |
| 1202907 | 1203145 | ISSod12 | 78.24 | 8.00E-06 |
| 1195099 | 1195337 | ISSod12 | 78.24 | 8.00E-06 |
| 2699641 | 2699879 | ISSod12 | 78.24 | 8.00E-06 |
| 2934396 | 2934634 | ISSod12 | 78.24 | 8.00E-06 |
| 3091404 | 3091642 | ISSod12 | 78.24 | 8.00E-06 |
| 662311 | 662549 | ISSod12 | 78.24 | 8.00E-06 |

| | | | | |
|---|---|---|---|---|
| 567765 | 568003 | ISSod12 | 78.24 | 8.00E-06 |
| 457996 | 458234 | ISSod12 | 78.24 | 8.00E-06 |
| 529035 | 529112 | ISPrre1 | 87.18 | 5.00E-10 |
| 1601998 | 1602075 | ISPrre1 | 87.18 | 5.00E-10 |
| 1553751 | 1553828 | ISPrre1 | 87.18 | 5.00E-10 |
| 1196264 | 1196341 | ISPrre1 | 87.18 | 5.00E-10 |
| 529047 | 529119 | ISVsa11 | 87.67 | 2.00E-09 |
| 1601991 | 1602063 | ISVsa11 | 87.67 | 2.00E-09 |
| 1553744 | 1553816 | ISVsa11 | 87.67 | 2.00E-09 |
| 1196257 | 1196329 | ISVsa11 | 87.67 | 2.00E-09 |
| 1626279 | 1626403 | ISCps7 | 83.2 | 2.00E-09 |
| 3562545 | 3562612 | ISEcret9 | 88.24 | 8.00E-09 |
| 1928552 | 1928619 | ISPsy43 | 88.24 | 8.00E-09 |
| 2214257 | 2214308 | ISSpu23 | 92.31 | 8.00E-09 |
| 678054 | 678216 | ISSma12 | 80.37 | 3.00E-08 |
| 678872 | 678934 | ISSma12 | 87.3 | 8.00E-06 |
| 529044 | 529081 | ISSba5 | 97.37 | 1.00E-07 |
| 1602029 | 1602066 | ISSba5 | 97.37 | 1.00E-07 |
| 1553782 | 1553819 | ISSba5 | 97.37 | 1.00E-07 |
| 1196295 | 1196332 | ISSba5 | 97.37 | 1.00E-07 |
| 677975 | 678027 | ISSpu18 | 90.57 | 5.00E-07 |
| 677831 | 677898 | ISSpu18 | 88.24 | 5.00E-07 |
| 2449275 | 2449314 | ISAbe16 | 95 | 2.00E-06 |
| 1565068 | 1565107 | ISAbe16 | 95 | 2.00E-06 |
| 1594544 | 1594583 | ISAbe16 | 95 | 2.00E-06 |
| 1621011 | 1621050 | ISAbe16 | 95 | 2.00E-06 |
| 1571037 | 1571076 | ISAbe16 | 95 | 2.00E-06 |
| 1562715 | 1562754 | ISAbe16 | 95 | 2.00E-06 |
| 2481670 | 2481709 | ISAbe16 | 95 | 2.00E-06 |
| 1204714 | 1204753 | ISAbe16 | 95 | 2.00E-06 |
| 1201679 | 1201718 | ISAbe16 | 95 | 2.00E-06 |
| 320688 | 320727 | ISAbe16 | 95 | 2.00E-06 |
| 529048 | 529099 | ISEc31 | 90.38 | 2.00E-06 |
| 1602011 | 1602062 | ISEc31 | 90.38 | 2.00E-06 |
| 1553764 | 1553815 | ISEc31 | 90.38 | 2.00E-06 |
| 1196277 | 1196328 | ISEc31 | 90.38 | 2.00E-06 |
| 529048 | 529119 | ISAlg | 86.11 | 2.00E-06 |
| 1601991 | 1602062 | ISAlg | 86.11 | 2.00E-06 |
| 1553744 | 1553815 | ISAlg | 86.11 | 2.00E-06 |
| 1196257 | 1196328 | ISAlg | 86.11 | 2.00E-06 |
| 2449932 | 2449999 | ISShes1 | 88.57 | 2.00E-06 |
| 1564383 | 1564450 | ISShes1 | 88.57 | 2.00E-06 |
| 1593859 | 1593926 | ISShes1 | 88.57 | 2.00E-06 |
| 1621668 | 1621735 | ISShes1 | 88.57 | 2.00E-06 |
| 1571694 | 1571761 | ISShes1 | 88.57 | 2.00E-06 |
| 1563372 | 1563439 | ISShes1 | 88.57 | 2.00E-06 |
| 2480985 | 2481052 | ISShes1 | 88.57 | 2.00E-06 |
| 1205371 | 1205438 | ISShes1 | 88.57 | 2.00E-06 |
| 1202336 | 1202403 | ISShes1 | 88.57 | 2.00E-06 |
| 321345 | 321412 | ISShes1 | 88.57 | 2.00E-06 |
| 529041 | 529075 | ISPa107 | 97.14 | 8.00E-06 |
| 1602035 | 1602069 | ISPa107 | 97.14 | 8.00E-06 |

| | | | | |
|---|---|---|---|---|
| 1553788 | 1553822 | ISPa107 | 97.14 | 8.00E-06 |
| 1196301 | 1196335 | ISPa107 | 97.14 | 8.00E-06 |
| 529041 | 529075 | ISPa98 | 97.14 | 8.00E-06 |
| 1602035 | 1602069 | ISPa98 | 97.14 | 8.00E-06 |
| 1553788 | 1553822 | ISPa98 | 97.14 | 8.00E-06 |
| 1196301 | 1196335 | ISPa98 | 97.14 | 8.00E-06 |
| 529047 | 529081 | ISShfr6 | 97.14 | 8.00E-06 |
| 1602029 | 1602063 | ISShfr6 | 97.14 | 8.00E-06 |
| 1553782 | 1553816 | ISShfr6 | 97.14 | 8.00E-06 |
| 1196295 | 1196329 | ISShfr6 | 97.14 | 8.00E-06 |
| 678965 | 679026 | ISAlw24 | 88.71 | 3.00E-05 |
| 2214279 | 2214308 | ISEc40 | 100 | 3.00E-05 |
| 693195 | 693244 | ISShes13 | 90 | 3.00E-05 |
| 2214997 | 2215981 | ISVa2 | 91.68 | 0 |
| 181913 | 182897 | ISVa2 | 91.68 | 0 |
| 394379 | 395363 | ISVa2 | 91.68 | 0 |
| 1705432 | 1706416 | ISVa2 | 91.68 | 0 |
| 1689549 | 1690533 | ISVa2 | 91.68 | 0 |
| 1007499 | 1008483 | ISVa2 | 91.68 | 0 |
| 1436559 | 1437543 | ISVa2 | 91.68 | 0 |
| 787323 | 788307 | ISVa2 | 91.68 | 0 |
| 774160 | 775144 | ISVa2 | 91.68 | 0 |
| 1591966 | 1592950 | ISVa2 | 91.68 | 0 |
| 1646306 | 1647290 | ISVa2 | 91.68 | 0 |
| 1665095 | 1666079 | ISVa2 | 91.68 | 0 |
| 1798644 | 1799628 | ISVa2 | 91.68 | 0 |
| 2125083 | 2126067 | ISVa2 | 91.68 | 0 |
| 2214980 | 2215981 | ISVal1 | 90.92 | 0 |
| 181913 | 182914 | ISVal1 | 90.92 | 0 |
| 394379 | 395380 | ISVal1 | 90.92 | 0 |
| 1705415 | 1706416 | ISVal1 | 90.92 | 0 |
| 1689532 | 1690533 | ISVal1 | 90.92 | 0 |
| 1007482 | 1008483 | ISVal1 | 90.92 | 0 |
| 1436559 | 1437560 | ISVal1 | 90.92 | 0 |
| 787306 | 788307 | ISVal1 | 90.92 | 0 |
| 774143 | 775144 | ISVal1 | 90.92 | 0 |
| 1591966 | 1592967 | ISVal1 | 90.92 | 0 |
| 1646306 | 1647307 | ISVal1 | 90.92 | 0 |
| 1665095 | 1666096 | ISVal1 | 90.92 | 0 |
| 1798644 | 1799645 | ISVal1 | 90.92 | 0 |
| 2125083 | 2126084 | ISVal1 | 90.92 | 0 |
| 2214993 | 2215913 | ISVpa3 | 83.82 | 0 |
| 181981 | 182901 | ISVpa3 | 83.82 | 0 |
| 394447 | 395367 | ISVpa3 | 83.82 | 0 |
| 1705428 | 1706348 | ISVpa3 | 83.82 | 0 |
| 1689545 | 1690465 | ISVpa3 | 83.82 | 0 |
| 1007495 | 1008415 | ISVpa3 | 83.82 | 0 |
| 1436627 | 1437547 | ISVpa3 | 83.82 | 0 |
| 787319 | 788239 | ISVpa3 | 83.82 | 0 |
| 774156 | 775076 | ISVpa3 | 83.82 | 0 |
| 1592034 | 1592954 | ISVpa3 | 83.82 | 0 |
| 1646374 | 1647294 | ISVpa3 | 83.82 | 0 |

| | | | | |
|---|---|---|---|---|
| 1665163 | 1666083 | ISVpa3 | 83.82 | 0 |
| 1798712 | 1799632 | ISVpa3 | 83.82 | 0 |
| 2125151 | 2126071 | ISVpa3 | 83.82 | 0 |
| 2214995 | 2215913 | ISVvu5 | 83.46 | 4.00E-173 |
| 181981 | 182899 | ISVvu5 | 83.46 | 4.00E-173 |
| 394447 | 395365 | ISVvu5 | 83.46 | 4.00E-173 |
| 1705430 | 1706348 | ISVvu5 | 83.46 | 4.00E-173 |
| 1689547 | 1690465 | ISVvu5 | 83.46 | 4.00E-173 |
| 1007497 | 1008415 | ISVvu5 | 83.46 | 4.00E-173 |
| 1436627 | 1437545 | ISVvu5 | 83.46 | 4.00E-173 |
| 787321 | 788239 | ISVvu5 | 83.46 | 4.00E-173 |
| 774158 | 775076 | ISVvu5 | 83.46 | 4.00E-173 |
| 1592034 | 1592952 | ISVvu5 | 83.46 | 4.00E-173 |
| 1646374 | 1647292 | ISVvu5 | 83.46 | 4.00E-173 |
| 1665163 | 1666081 | ISVvu5 | 83.46 | 4.00E-173 |
| 1798712 | 1799630 | ISVvu5 | 83.46 | 4.00E-173 |
| 2125151 | 2126069 | ISVvu5 | 83.46 | 4.00E-173 |
| 2215002 | 2215558 | ISVbsp3 | 83.12 | 1.00E-95 |
| 182336 | 182892 | ISVbsp3 | 83.12 | 1.00E-95 |
| 394802 | 395358 | ISVbsp3 | 83.12 | 1.00E-95 |
| 1705437 | 1705993 | ISVbsp3 | 83.12 | 1.00E-95 |
| 1689554 | 1690110 | ISVbsp3 | 83.12 | 1.00E-95 |
| 1007504 | 1008060 | ISVbsp3 | 83.12 | 1.00E-95 |
| 1436982 | 1437538 | ISVbsp3 | 83.12 | 1.00E-95 |
| 787328 | 787884 | ISVbsp3 | 83.12 | 1.00E-95 |
| 774165 | 774721 | ISVbsp3 | 83.12 | 1.00E-95 |
| 1592389 | 1592945 | ISVbsp3 | 83.12 | 1.00E-95 |
| 1646729 | 1647285 | ISVbsp3 | 83.12 | 1.00E-95 |
| 1665518 | 1666074 | ISVbsp3 | 83.12 | 1.00E-95 |
| 1799067 | 1799623 | ISVbsp3 | 83.12 | 1.00E-95 |
| 2125506 | 2126062 | ISVbsp3 | 83.12 | 1.00E-95 |
| 2215696 | 2215897 | ISVbsp3 | 80.88 | 1.00E-12 |
| 181997 | 182198 | ISVbsp3 | 80.88 | 1.00E-12 |
| 394463 | 394664 | ISVbsp3 | 80.88 | 1.00E-12 |
| 1706131 | 1706332 | ISVbsp3 | 80.88 | 1.00E-12 |
| 1690248 | 1690449 | ISVbsp3 | 80.88 | 1.00E-12 |
| 1008198 | 1008399 | ISVbsp3 | 80.88 | 1.00E-12 |
| 1436643 | 1436844 | ISVbsp3 | 80.88 | 1.00E-12 |
| 788022 | 788223 | ISVbsp3 | 80.88 | 1.00E-12 |
| 774859 | 775060 | ISVbsp3 | 80.88 | 1.00E-12 |
| 1592050 | 1592251 | ISVbsp3 | 80.88 | 1.00E-12 |
| 1646390 | 1646591 | ISVbsp3 | 80.88 | 1.00E-12 |
| 1665179 | 1665380 | ISVbsp3 | 80.88 | 1.00E-12 |
| 1798728 | 1798929 | ISVbsp3 | 80.88 | 1.00E-12 |
| 2125167 | 2125368 | ISVbsp3 | 80.88 | 1.00E-12 |
| 2215002 | 2215981 | ISVch8 | 78.08 | 1.00E-55 |
| 181913 | 182892 | ISVch8 | 78.08 | 1.00E-55 |
| 394379 | 395358 | ISVch8 | 78.08 | 1.00E-55 |
| 1705437 | 1706416 | ISVch8 | 78.08 | 1.00E-55 |
| 1689554 | 1690533 | ISVch8 | 78.08 | 1.00E-55 |
| 1007504 | 1008483 | ISVch8 | 78.08 | 1.00E-55 |
| 1436559 | 1437538 | ISVch8 | 78.08 | 1.00E-55 |

| | | | | |
|---|---|---|---|---|
| 787328 | 788307 | ISVch8 | 78.08 | 1.00E-55 |
| 774165 | 775144 | ISVch8 | 78.08 | 1.00E-55 |
| 1591966 | 1592945 | ISVch8 | 78.08 | 1.00E-55 |
| 1646306 | 1647285 | ISVch8 | 78.08 | 1.00E-55 |
| 1665095 | 1666074 | ISVch8 | 78.08 | 1.00E-55 |
| 1798644 | 1799623 | ISVch8 | 78.08 | 1.00E-55 |
| 2125083 | 2126062 | ISVch8 | 78.08 | 1.00E-55 |
| 476409 | 476485 | ISVch8 | 89.74 | 1.00E-12 |
| 2215503 | 2215906 | ISVba2 | 79.95 | 3.00E-35 |
| 181988 | 182391 | ISVba2 | 79.95 | 3.00E-35 |
| 394454 | 394857 | ISVba2 | 79.95 | 3.00E-35 |
| 1705938 | 1706341 | ISVba2 | 79.95 | 3.00E-35 |
| 1690055 | 1690458 | ISVba2 | 79.95 | 3.00E-35 |
| 1008005 | 1008408 | ISVba2 | 79.95 | 3.00E-35 |
| 1436634 | 1437037 | ISVba2 | 79.95 | 3.00E-35 |
| 787829 | 788232 | ISVba2 | 79.95 | 3.00E-35 |
| 774666 | 775069 | ISVba2 | 79.95 | 3.00E-35 |
| 1592041 | 1592444 | ISVba2 | 79.95 | 3.00E-35 |
| 1646381 | 1646784 | ISVba2 | 79.95 | 3.00E-35 |
| 1665170 | 1665573 | ISVba2 | 79.95 | 3.00E-35 |
| 1798719 | 1799122 | ISVba2 | 79.95 | 3.00E-35 |
| 2125158 | 2125561 | ISVba2 | 79.95 | 3.00E-35 |
| 2215808 | 2215914 | ISV-M52 | 89.72 | 4.00E-25 |
| 181980 | 182086 | ISV-M52 | 89.72 | 4.00E-25 |
| 394446 | 394552 | ISV-M52 | 89.72 | 4.00E-25 |
| 1706243 | 1706349 | ISV-M52 | 89.72 | 4.00E-25 |
| 1690360 | 1690466 | ISV-M52 | 89.72 | 4.00E-25 |
| 1008310 | 1008416 | ISV-M52 | 89.72 | 4.00E-25 |
| 1436626 | 1436732 | ISV-M52 | 89.72 | 4.00E-25 |
| 788134 | 788240 | ISV-M52 | 89.72 | 4.00E-25 |
| 774971 | 775077 | ISV-M52 | 89.72 | 4.00E-25 |
| 1592033 | 1592139 | ISV-M52 | 89.72 | 4.00E-25 |
| 1646373 | 1646479 | ISV-M52 | 89.72 | 4.00E-25 |
| 1665162 | 1665268 | ISV-M52 | 89.72 | 4.00E-25 |
| 1798711 | 1798817 | ISV-M52 | 89.72 | 4.00E-25 |
| 2125150 | 2125256 | ISV-M52 | 89.72 | 4.00E-25 |
| 2215002 | 2215207 | ISV-M52 | 82.04 | 8.00E-20 |
| 182687 | 182892 | ISV-M52 | 82.04 | 8.00E-20 |
| 395153 | 395358 | ISV-M52 | 82.04 | 8.00E-20 |
| 1705437 | 1705642 | ISV-M52 | 82.04 | 8.00E-20 |
| 1689554 | 1689759 | ISV-M52 | 82.04 | 8.00E-20 |
| 1007504 | 1007709 | ISV-M52 | 82.04 | 8.00E-20 |
| 1437333 | 1437538 | ISV-M52 | 82.04 | 8.00E-20 |
| 787328 | 787533 | ISV-M52 | 82.04 | 8.00E-20 |
| 774165 | 774370 | ISV-M52 | 82.04 | 8.00E-20 |
| 1592740 | 1592945 | ISV-M52 | 82.04 | 8.00E-20 |
| 1647080 | 1647285 | ISV-M52 | 82.04 | 8.00E-20 |
| 1665869 | 1666074 | ISV-M52 | 82.04 | 8.00E-20 |
| 1799418 | 1799623 | ISV-M52 | 82.04 | 8.00E-20 |
| 2125857 | 2126062 | ISV-M52 | 82.04 | 8.00E-20 |
| 476487 | 476553 | ISV-M52 | 89.55 | 8.00E-11 |
| 476416 | 476484 | ISV-M52 | 87.14 | 2.00E-05 |

| | | | | |
|---|---|---|---|---|
| 2215797 | 2215900 | ISSpu11 | 87.5 | 1.00E-18 |
| 181994 | 182097 | ISSpu11 | 87.5 | 1.00E-18 |
| 394460 | 394563 | ISSpu11 | 87.5 | 1.00E-18 |
| 1706232 | 1706335 | ISSpu11 | 87.5 | 1.00E-18 |
| 1690349 | 1690452 | ISSpu11 | 87.5 | 1.00E-18 |
| 1008299 | 1008402 | ISSpu11 | 87.5 | 1.00E-18 |
| 1436640 | 1436743 | ISSpu11 | 87.5 | 1.00E-18 |
| 788123 | 788226 | ISSpu11 | 87.5 | 1.00E-18 |
| 774960 | 775063 | ISSpu11 | 87.5 | 1.00E-18 |
| 1592047 | 1592150 | ISSpu11 | 87.5 | 1.00E-18 |
| 1646387 | 1646490 | ISSpu11 | 87.5 | 1.00E-18 |
| 1665176 | 1665279 | ISSpu11 | 87.5 | 1.00E-18 |
| 1798725 | 1798828 | ISSpu11 | 87.5 | 1.00E-18 |
| 2125164 | 2125267 | ISSpu11 | 87.5 | 1.00E-18 |
| 2215694 | 2215760 | ISSpu11 | 89.55 | 8.00E-11 |
| 182134 | 182200 | ISSpu11 | 89.55 | 8.00E-11 |
| 394600 | 394666 | ISSpu11 | 89.55 | 8.00E-11 |
| 1706129 | 1706195 | ISSpu11 | 89.55 | 8.00E-11 |
| 1690246 | 1690312 | ISSpu11 | 89.55 | 8.00E-11 |
| 1008196 | 1008262 | ISSpu11 | 89.55 | 8.00E-11 |
| 1436780 | 1436846 | ISSpu11 | 89.55 | 8.00E-11 |
| 788020 | 788086 | ISSpu11 | 89.55 | 8.00E-11 |
| 774857 | 774923 | ISSpu11 | 89.55 | 8.00E-11 |
| 1592187 | 1592253 | ISSpu11 | 89.55 | 8.00E-11 |
| 1646527 | 1646593 | ISSpu11 | 89.55 | 8.00E-11 |
| 1665316 | 1665382 | ISSpu11 | 89.55 | 8.00E-11 |
| 1798865 | 1798931 | ISSpu11 | 89.55 | 8.00E-11 |
| 2125304 | 2125370 | ISSpu11 | 89.55 | 8.00E-11 |
| 2215002 | 2215270 | ISSpu11 | 78.44 | 1.00E-09 |
| 182624 | 182892 | ISSpu11 | 78.44 | 1.00E-09 |
| 395090 | 395358 | ISSpu11 | 78.44 | 1.00E-09 |
| 1705437 | 1705705 | ISSpu11 | 78.44 | 1.00E-09 |
| 1689554 | 1689822 | ISSpu11 | 78.44 | 1.00E-09 |
| 1007504 | 1007772 | ISSpu11 | 78.44 | 1.00E-09 |
| 1437270 | 1437538 | ISSpu11 | 78.44 | 1.00E-09 |
| 787328 | 787596 | ISSpu11 | 78.44 | 1.00E-09 |
| 774165 | 774433 | ISSpu11 | 78.44 | 1.00E-09 |
| 1592677 | 1592945 | ISSpu11 | 78.44 | 1.00E-09 |
| 1647017 | 1647285 | ISSpu11 | 78.44 | 1.00E-09 |
| 1665806 | 1666074 | ISSpu11 | 78.44 | 1.00E-09 |
| 1799355 | 1799623 | ISSpu11 | 78.44 | 1.00E-09 |
| 2125794 | 2126062 | ISSpu11 | 78.44 | 1.00E-09 |
| 2215443 | 2215534 | ISSpu11 | 84.78 | 5.00E-09 |
| 182360 | 182451 | ISSpu11 | 84.78 | 5.00E-09 |
| 394826 | 394917 | ISSpu11 | 84.78 | 5.00E-09 |
| 1705878 | 1705969 | ISSpu11 | 84.78 | 5.00E-09 |
| 1689995 | 1690086 | ISSpu11 | 84.78 | 5.00E-09 |
| 1007945 | 1008036 | ISSpu11 | 84.78 | 5.00E-09 |
| 1437006 | 1437097 | ISSpu11 | 84.78 | 5.00E-09 |
| 787769 | 787860 | ISSpu11 | 84.78 | 5.00E-09 |
| 774606 | 774697 | ISSpu11 | 84.78 | 5.00E-09 |
| 1592413 | 1592504 | ISSpu11 | 84.78 | 5.00E-09 |

| | | | | |
|---|---|---|---|---|
| 1646753 | 1646844 | ISSpu11 | 84.78 | 5.00E-09 |
| 1665542 | 1665633 | ISSpu11 | 84.78 | 5.00E-09 |
| 1799091 | 1799182 | ISSpu11 | 84.78 | 5.00E-09 |
| 2125530 | 2125621 | ISSpu11 | 84.78 | 5.00E-09 |
| 724794 | 724871 | ISEc52 | 91.03 | 2.00E-17 |
| 724809 | 724878 | ISSlo2 | 92.86 | 2.00E-17 |
| 724807 | 724871 | IS911 | 93.85 | 8.00E-17 |
| 724773 | 724878 | ISCfr25 | 85.85 | 5.00E-15 |
| 725195 | 725253 | ISCfr25 | 89.83 | 2.00E-08 |
| 724472 | 724505 | ISCfr25 | 97.06 | 2.00E-05 |
| 724806 | 724871 | ISLad1 | 92.42 | 5.00E-15 |
| 2215809 | 2215885 | ISSpu20 | 89.61 | 2.00E-14 |
| 182009 | 182085 | ISSpu20 | 89.61 | 2.00E-14 |
| 394475 | 394551 | ISSpu20 | 89.61 | 2.00E-14 |
| 1706244 | 1706320 | ISSpu20 | 89.61 | 2.00E-14 |
| 1690361 | 1690437 | ISSpu20 | 89.61 | 2.00E-14 |
| 1008311 | 1008387 | ISSpu20 | 89.61 | 2.00E-14 |
| 1436655 | 1436731 | ISSpu20 | 89.61 | 2.00E-14 |
| 788135 | 788211 | ISSpu20 | 89.61 | 2.00E-14 |
| 774972 | 775048 | ISSpu20 | 89.61 | 2.00E-14 |
| 1592062 | 1592138 | ISSpu20 | 89.61 | 2.00E-14 |
| 1646402 | 1646478 | ISSpu20 | 89.61 | 2.00E-14 |
| 1665191 | 1665267 | ISSpu20 | 89.61 | 2.00E-14 |
| 1798740 | 1798816 | ISSpu20 | 89.61 | 2.00E-14 |
| 2125179 | 2125255 | ISSpu20 | 89.61 | 2.00E-14 |
| 724800 | 724872 | ISSpu4 | 90.41 | 2.00E-14 |
| 724794 | 724871 | ISBrsa1 | 88.46 | 1.00E-12 |
| 724800 | 724878 | ISVch4 | 87.34 | 8.00E-11 |
| 2215815 | 2215897 | ISSod12 | 86.75 | 8.00E-11 |
| 181997 | 182079 | ISSod12 | 86.75 | 8.00E-11 |
| 394463 | 394545 | ISSod12 | 86.75 | 8.00E-11 |
| 1706250 | 1706332 | ISSod12 | 86.75 | 8.00E-11 |
| 1690367 | 1690449 | ISSod12 | 86.75 | 8.00E-11 |
| 1008317 | 1008399 | ISSod12 | 86.75 | 8.00E-11 |
| 1436643 | 1436725 | ISSod12 | 86.75 | 8.00E-11 |
| 788141 | 788223 | ISSod12 | 86.75 | 8.00E-11 |
| 774978 | 775060 | ISSod12 | 86.75 | 8.00E-11 |
| 1592050 | 1592132 | ISSod12 | 86.75 | 8.00E-11 |
| 1646390 | 1646472 | ISSod12 | 86.75 | 8.00E-11 |
| 1665179 | 1665261 | ISSod12 | 86.75 | 8.00E-11 |
| 1798728 | 1798810 | ISSod12 | 86.75 | 8.00E-11 |
| 2125167 | 2125249 | ISSod12 | 86.75 | 8.00E-11 |
| 2215458 | 2215534 | ISSod12 | 85.71 | 3.00E-07 |
| 182360 | 182436 | ISSod12 | 85.71 | 3.00E-07 |
| 394826 | 394902 | ISSod12 | 85.71 | 3.00E-07 |
| 1705893 | 1705969 | ISSod12 | 85.71 | 3.00E-07 |
| 1690010 | 1690086 | ISSod12 | 85.71 | 3.00E-07 |
| 1007960 | 1008036 | ISSod12 | 85.71 | 3.00E-07 |
| 1437006 | 1437082 | ISSod12 | 85.71 | 3.00E-07 |
| 787784 | 787860 | ISSod12 | 85.71 | 3.00E-07 |
| 774621 | 774697 | ISSod12 | 85.71 | 3.00E-07 |
| 1592413 | 1592489 | ISSod12 | 85.71 | 3.00E-07 |

| | | | | |
|---|---|---|---|---|
| 1646753 | 1646829 | ISSod12 | 85.71 | 3.00E-07 |
| 1665542 | 1665618 | ISSod12 | 85.71 | 3.00E-07 |
| 1799091 | 1799167 | ISSod12 | 85.71 | 3.00E-07 |
| 2125530 | 2125606 | ISSod12 | 85.71 | 3.00E-07 |
| 2215002 | 2215240 | ISSod12 | 78.24 | 4.00E-06 |
| 182654 | 182892 | ISSod12 | 78.24 | 4.00E-06 |
| 395120 | 395358 | ISSod12 | 78.24 | 4.00E-06 |
| 1705437 | 1705675 | ISSod12 | 78.24 | 4.00E-06 |
| 1689554 | 1689792 | ISSod12 | 78.24 | 4.00E-06 |
| 1007504 | 1007742 | ISSod12 | 78.24 | 4.00E-06 |
| 1437300 | 1437538 | ISSod12 | 78.24 | 4.00E-06 |
| 787328 | 787566 | ISSod12 | 78.24 | 4.00E-06 |
| 774165 | 774403 | ISSod12 | 78.24 | 4.00E-06 |
| 1592707 | 1592945 | ISSod12 | 78.24 | 4.00E-06 |
| 1647047 | 1647285 | ISSod12 | 78.24 | 4.00E-06 |
| 1665836 | 1666074 | ISSod12 | 78.24 | 4.00E-06 |
| 1799385 | 1799623 | ISSod12 | 78.24 | 4.00E-06 |
| 2125824 | 2126062 | ISSod12 | 78.24 | 4.00E-06 |
| 724794 | 724871 | ISPrre1 | 87.18 | 3.00E-10 |
| 724806 | 724878 | ISVsa11 | 87.67 | 1.00E-09 |
| 724803 | 724840 | ISSba5 | 97.37 | 7.00E-08 |
| 725642 | 725682 | ISSba5 | 92.68 | 7.00E-05 |
| 725701 | 725733 | ISSba5 | 96.97 | 7.00E-05 |
| 479862 | 479905 | ISVa15 | 93.18 | 1.00E-06 |
| 724807 | 724858 | ISEc31 | 90.38 | 1.00E-06 |
| 725701 | 725733 | ISEc31 | 96.97 | 7.00E-05 |
| 724807 | 724878 | ISAlg | 86.11 | 1.00E-06 |
| 724800 | 724834 | ISPa107 | 97.14 | 4.00E-06 |
| 724800 | 724834 | ISPa98 | 97.14 | 4.00E-06 |
| 724806 | 724840 | ISShfr6 | 97.14 | 4.00E-06 |
| 725642 | 725682 | ISShfr6 | 92.68 | 7.00E-05 |
| 2215509 | 2215553 | ISEc28 | 91.11 | 7.00E-05 |
| 182341 | 182385 | ISEc28 | 91.11 | 7.00E-05 |
| 394807 | 394851 | ISEc28 | 91.11 | 7.00E-05 |
| 1705944 | 1705988 | ISEc28 | 91.11 | 7.00E-05 |
| 1690061 | 1690105 | ISEc28 | 91.11 | 7.00E-05 |
| 1008011 | 1008055 | ISEc28 | 91.11 | 7.00E-05 |
| 1436987 | 1437031 | ISEc28 | 91.11 | 7.00E-05 |
| 787835 | 787879 | ISEc28 | 91.11 | 7.00E-05 |
| 774672 | 774716 | ISEc28 | 91.11 | 7.00E-05 |
| 1592394 | 1592438 | ISEc28 | 91.11 | 7.00E-05 |
| 1646734 | 1646778 | ISEc28 | 91.11 | 7.00E-05 |
| 1665523 | 1665567 | ISEc28 | 91.11 | 7.00E-05 |
| 1799072 | 1799116 | ISEc28 | 91.11 | 7.00E-05 |
| 2125511 | 2125555 | ISEc28 | 91.11 | 7.00E-05 |
| 89544 | 90783 | ISVal2 | 92.92 | 0 |
| 74387 | 75620 | ISVal2 | 92.44 | 0 |
| 73395 | 74389 | ISVal2 | 91.71 | 0 |
| 89544 | 90783 | ISVpa2 | 87.77 | 0 |
| 74387 | 75620 | ISVpa2 | 87.21 | 0 |
| 73395 | 74389 | ISVpa2 | 85.51 | 0 |
| 89544 | 90783 | ISVisp3 | 86.16 | 0 |

| | | | | |
|---|---|---|---|---|
| 74387 | 75620 | ISVisp3 | 85.84 | 0 |
| 73394 | 74389 | ISVisp3 | 84.43 | 0 |
| 89544 | 90775 | ISVpe1 | 85.67 | 0 |
| 74387 | 75612 | ISVpe1 | 85.43 | 0 |
| 73395 | 74389 | ISVpe1 | 83.42 | 0 |
| 89544 | 90775 | ISVisp5 | 85.67 | 0 |
| 74387 | 75612 | ISVisp5 | 85.43 | 0 |
| 73395 | 74389 | ISVisp5 | 83.42 | 0 |
| 5434 | 5659 | ISVa19 | 92.04 | 8.00E-81 |
| 75369 | 75408 | ISAbe16 | 95 | 5.00E-08 |
| 90532 | 90571 | ISAbe16 | 95 | 5.00E-08 |
| 75501 | 75561 | ISSpu1 | 86.89 | 3.00E-06 |
| 90664 | 90724 | ISSpu1 | 86.89 | 3.00E-06 |
| 74154 | 74192 | ISShes1 | 92.31 | 5.00E-05 |
| 75143 | 75181 | ISShes1 | 92.31 | 5.00E-05 |
| 90306 | 90344 | ISShes1 | 92.31 | 5.00E-05 |
| 75369 | 75407 | ISPto4 | 92.31 | 5.00E-05 |
| 90532 | 90570 | ISPto4 | 92.31 | 5.00E-05 |
| 3604 | 4153 | ISVal1 | 92.73 | 0 |
| 3228 | 3607 | ISVal1 | 87.11 | 7.00E-99 |
| 61766 | 61917 | ISVal1 | 86.93 | 7.00E-28 |
| 3604 | 4153 | ISVa2 | 92.73 | 0 |
| 3245 | 3605 | ISVa2 | 89.75 | 4.00E-116 |
| 61765 | 61917 | ISVa2 | 91.5 | 2.00E-49 |
| 3241 | 3590 | ISVpa3 | 88 | 1.00E-97 |
| 3641 | 4085 | ISVpa3 | 82.7 | 8.00E-71 |
| 61772 | 61842 | ISVpa3 | 85.92 | 1.00E-07 |
| 3243 | 3590 | ISVvu5 | 86.78 | 6.00E-87 |
| 3641 | 4085 | ISVvu5 | 83.37 | 6.00E-78 |
| 61772 | 61842 | ISVvu5 | 84.51 | 4.00E-05 |
| 3250 | 3587 | ISVbsp3 | 83.14 | 1.00E-54 |
| 3641 | 3730 | ISVbsp3 | 92.22 | 5.00E-26 |
| 3868 | 4069 | ISVbsp3 | 80.88 | 4.00E-14 |
| 3675 | 4078 | ISVba2 | 79.95 | 8.00E-37 |
| 61805 | 61835 | ISVba2 | 96.77 | 4.00E-05 |
| 3250 | 3599 | ISVch8 | 80 | 8.00E-31 |
| 3980 | 4153 | ISVch8 | 83.43 | 4.00E-20 |
| 61766 | 61917 | ISVch8 | 83.66 | 2.00E-16 |
| 3639 | 3760 | ISVch8 | 81.15 | 6.00E-07 |
| 62180 | 62351 | ISVsa9 | 84.3 | 3.00E-27 |
| 3980 | 4086 | ISV-M52 | 89.72 | 1.00E-26 |
| 3250 | 3455 | ISV-M52 | 82.04 | 3.00E-21 |
| 3562 | 3607 | ISV-M52 | 93.48 | 2.00E-09 |
| 3969 | 4072 | ISSpu11 | 87.5 | 4.00E-20 |
| 3866 | 3932 | ISSpu11 | 89.55 | 2.00E-12 |
| 3250 | 3518 | ISSpu11 | 78.44 | 4.00E-11 |
| 3615 | 3706 | ISSpu11 | 84.78 | 1.00E-10 |
| 3981 | 4057 | ISSpu20 | 89.61 | 6.00E-16 |
| 2973 | 3031 | ISVha3 | 93.22 | 2.00E-12 |
| 3987 | 4069 | ISSod12 | 86.75 | 2.00E-12 |
| 3630 | 3706 | ISSod12 | 85.71 | 9.00E-09 |
| 3250 | 3488 | ISSod12 | 78.24 | 1.00E-07 |

| | | | | |
|---|---|---|---|---|
| 3681 | 3725 | ISEc28 | 91.11 | 2.00E-06 |
| 3681 | 3723 | ISPrre2 | 90.7 | 4.00E-05 |
| 24164 | 24316 | ISVa2 | 91.5 | 2.00E-49 |
| 29181 | 29416 | ISShwo3 | 85.59 | 8.00E-49 |
| 28298 | 28477 | ISShwo3 | 80.56 | 5.00E-13 |
| 29183 | 29416 | ISPtu2 | 86.02 | 2.00E-46 |
| 29181 | 29416 | ISSpi5 | 85.23 | 1.00E-44 |
| 28298 | 28387 | ISSpi5 | 84.44 | 2.00E-09 |
| 29134 | 29413 | IS492 | 83.39 | 7.00E-37 |
| 28298 | 28477 | IS492 | 81.67 | 9.00E-18 |
| 28555 | 28646 | IS492 | 82.61 | 8.00E-06 |
| 24165 | 24316 | ISVal1 | 86.93 | 6.00E-28 |
| 28297 | 28641 | ISSde13 | 79.19 | 6.00E-19 |
| 28790 | 28999 | ISSde13 | 79.05 | 2.00E-09 |
| 24165 | 24316 | ISVch8 | 83.66 | 1.00E-16 |
| 29224 | 29348 | ISCps7 | 83.2 | 3.00E-11 |
| 24171 | 24241 | ISVpa3 | 85.92 | 1.00E-07 |
| 24204 | 24234 | ISVba2 | 96.77 | 3.00E-05 |
| 24171 | 24241 | ISVvu5 | 84.51 | 3.00E-05 |
| 56553 | 57634 | ISVa3 | 80.99 | 4.00E-146 |
| 52106 | 52921 | ISVa3 | 80.07 | 5.00E-90 |
| 56082 | 56153 | ISVa3 | 87.5 | 1.00E-10 |
| 59311 | 59499 | ISVa2 | 91.53 | 1.00E-63 |
| 59332 | 59499 | ISVal1 | 88.76 | 7.00E-40 |
| 53748 | 53993 | ISVvu1 | 81.45 | 1.00E-25 |
| 59312 | 59499 | ISVch8 | 82.54 | 5.00E-19 |
| 59332 | 59428 | ISV-M52 | 87.63 | 2.00E-18 |
| 59332 | 59425 | ISVba2 | 87.23 | 1.00E-16 |
| 59334 | 59407 | ISSpu11 | 90.54 | 1.00E-16 |
| 59334 | 59407 | ISSod12 | 90.54 | 1.00E-16 |
| 59324 | 59404 | ISSpu20 | 88.89 | 5.00E-16 |
| 59332 | 59428 | ISVpa3 | 86.6 | 5.00E-16 |
| 59332 | 59428 | ISVvu5 | 85.57 | 1.00E-13 |
| 55540 | 55586 | ISVha3 | 91.49 | 3.00E-05 |

**Table S9.** Prophage regions and associated genes in *V. harveyi* strain N8T11

| Region | Start Position | End Position | Orientation | Predicted Function |
|---|---|---|---|---|
| Chromosome 1 region 1 | 1981357 | 1982742 | - | Phage-like protein |
| | 1982747 | 1983091 | - | Hypothetical protein |
| | 1983093 | 1984583 | - | Phage-like protein |
| | 1984752 | 1984994 | - | Phage-like protein |
| | 1985000 | 1985227 | - | Phage-like protein |
| | 1985231 | 1985587 | - | Hypothetical protein |
| | 1985591 | 1986736 | - | Phage-like protein |
| | 1986720 | 1986935 | - | Hypothetical protein |
| | 1986938 | 1987210 | - | Hypothetical protein |
| | 1987466 | 1987834 | + | Hypothetical protein |
| | 1988003 | 1989733 | - | Hypothetical protein |
| | 1989723 | 1990574 | - | Hypothetical protein |
| | 1990692 | 1991612 | + | Transposase |
| Chromosome 2 region 1 | 589273 | 589587 | - | Hypothetical protein |
| | 589589 | 591190 | - | Hypothetical protein |
| | 591320 | 591550 | - | Hypothetical protein |
| | 591561 | 591806 | - | Hypothetical protein |
| | 591817 | 592155 | - | Phage-like protein |
| | 592157 | 593236 | - | Phage-like protein |
| | 593880 | 594164 | + | Hypothetical protein |
| | 594362 | 594607 | - | Hypothetical protein |
| | 594915 | 596054 | - | Hypothetical protein |
| | 596051 | 596365 | - | Hypothetical protein |
| | 596367 | 597959 | - | Hypothetical protein |
| | 598093 | 598323 | - | Hypothetical protein |
| | 598334 | 598579 | - | Hypothetical protein |
| | 598590 | 598940 | - | Hypothetical protein |
| | 598951 | 600255 | - | Phage-like protein |
| | 600424 | 600714 | + | Phage-like protein |
| | 601256 | 601996 | - | Hypothetical protein |
| Chromosome 2 region 2 | 1914443 | 1914459 | + | Attachment site |
| | 1914604 | 1915626 | - | Hypothetical protein |
| | 1915638 | 1916168 | - | Hypothetical protein |
| | 1916201 | 1916740 | - | Hypothetical protein |
| | 1916765 | 1917409 | - | Hypothetical protein |
| | 1917559 | 1917804 | + | Hypothetical protein |
| | 1917915 | 1918454 | + | Hypothetical protein |
| | 1918464 | 1918799 | + | Hypothetical protein |
| | 1918866 | 1919369 | + | Hypothetical protein |
| | 1919371 | 1919655 | + | Hypothetical protein |
| | 1919652 | 1920053 | + | Hypothetical protein |
| | 1920055 | 1920282 | + | Hypothetical protein |
| | 1920286 | 1920906 | + | Hypothetical protein |
| | 1920903 | 1921331 | + | Hypothetical protein |
| | 1921342 | 1924008 | + | Hypothetical protein |
| | 1924022 | 1924213 | + | Hypothetical protein |
| | 1924296 | 1924628 | - | Hypothetical protein |
| | 1924874 | 1925128 | + | Hypothetical protein |

| | | | |
|---|---|---|---|
| 1925147 | 1925398 | - | Hypothetical protein |
| 1925477 | 1926508 | - | Hypothetical protein |
| 1926505 | 1928256 | - | Hypothetical protein |
| 1928476 | 1929354 | + | Hypothetical protein |
| 1929394 | 1930407 | + | Hypothetical protein |
| 1930418 | 1931134 | + | Hypothetical protein |
| 1931267 | 1931686 | + | Hypothetical protein |
| 1931683 | 1932174 | + | Hypothetical protein |
| 1932158 | 1932814 | + | Hypothetical protein |
| 1932818 | 1933948 | + | Phage-like protein |
| 1933952 | 1934407 | + | Phage-like protein |
| 1934420 | 1934638 | + | Phage-like protein |
| 1934635 | 1935132 | + | Hypothetical protein |
| 1935129 | 1935359 | + | Hypothetical protein |
| 1935362 | 1935649 | + | Hypothetical protein |
| 1935646 | 1935909 | + | Hypothetical protein |
| 1935951 | 1936094 | + | Hypothetical protein |
| 1936120 | 1938003 | + | Phage-like protein |
| 1938003 | 1938344 | + | Phage-like protein |
| 1938344 | 1939531 | + | Hypothetical protein |
| 1939626 | 1940120 | + | Hypothetical protein |
| 1940132 | 1942561 | + | Phage-like protein |
| 1942561 | 1942830 | + | Hypothetical protein |
| 1942841 | 1943377 | + | Phage-like protein |
| 1943387 | 1944076 | + | Phage-like protein |
| 1944067 | 1944762 | + | Phage-like protein |
| 1944764 | 1944961 | + | Phage-like protein |
| 1945571 | 1945825 | + | Phage-like protein |
| 1945934 | 1945950 | + | Attachment site |

**Table S10.** Genetic features of plasmids in *V. harveyi* strain N8T11

| Plasmid | Gene name/ gene locus | Description |
|---|---|---|
| pN8T11a | AAIA71_28370 | Hypothetical protein |
| | AAIA71_28375 | Hypothetical protein |
| | AAIA71_28380 | Group II intron maturase-specific domain-containing protein |
| | AAIA71_28385 | Hypothetical protein |
| | AAIA71_ 28390 | AAA family ATPase |
| | AAIA71_28395 | Regulator |
| | AAIA71_ 28400 | IS256 family transposase |
| | AAIA71_ 28405 | Cyclic peptide export ABC transporter |
| | AAIA71_ 28410 | Extracellular solute-binding protein |
| | AAIA71_ 28415 | ABC transporter permease subunit |
| | AAIA71_ 28420 | Hypothetical protein |
| | AAIA71_ 28425 | ABC transporter permease subunit |
| | AAIA71_28430 | ABC transporter ATP-binding protein |
| | AAIA71_ 28435 | Nitro reductase |
| | AAIA71_ 28440 | 4'-phosphopantetheinyl transferase superfamily protein |
| | AAIA71_28445 | TonB-dependent siderophore receptor |
| | AAIA71_ 28450 | MbtH family NRPS accessory protein |
| | AAIA71_ 28455 | TauD/TfdA family dioxygenase |
| | AAIA71_ 28460 | Non-ribosomal peptide synthetase |
| | AAIA71_ 28465 | Amino acid adenylation domain-containing protein |
| | AAIA71_ 28470 | Phosphopantetheine-binding protein |
| | AAIA71_ 28475 | Amino acid adenylation domain-containing protein |
| | AAIA71_ 28480 | Thioesterase domain-containing protein |
| | AAIA71_ 28485 | Hypothetical protein |
| | AAIA71_ 28490 | Hypothetical protein |
| | AAIA71_ 28495 | Hypothetical protein |
| | AAIA71_ 28500 | Hypothetical protein |
| | AAIA71_ 28505 | Tyrosine-type recombinase integrase |
| | AAIA71_ 28510 | Hypothetical protein |
| | AAIA71_ 28515 | Hypothetical protein |
| | AAIA71_ 28520 | Tyrosine-type recombinase/integrase |
| | AAIA71_ 28525 | Hypothetical protein |
| | AAIA71_ 28530 | Replication initiator protein RctB domain-containing protein |
| | AAIA71_ 28535 | Hypothetical protein |
| | AAIA71_ 28540 | DNA replication terminus site-binding protein |
| | AAIA71_ 28545 | Hypothetical protein |
| | AAIA71_ 28550 | Hypothetical protein |
| | AAIA71_ 28555 | Hypothetical protein |
| | AAIA71_ 28560 | UyrD-helicase domain-containing protein |
| | AAIA71_ 28565 | Hypothetical protein |

| | | |
|---|---|---|
| | AAIA71_ 28570 | PD-(D/E)XK nuclease-like domain-containing protein |
| | AAIA71_ 28575 | ParA, family protein |
| | AAIA71_ 28580 | Hypothetical protein |
| | AAIA71_28585 | Hypothetical protein |
| | AAIA71_28590 | IS3 family transposase |
| | AAIA71_ 28595 | IS3 family transposase |
| | AAIA71_ 28600 | Hypothetical protein |
| | AAIA71_ 28605 | Hypothetical protein |
| | AAIA71_ 28610 | Site-specific integrase |
| | AAIA71_28615 | DUF2913 family protein |
| | AAIA71_ 28620 | Type Il toxin-antitoxin system Pemk/MazF family toxin |
| | AAIA71_28625 | Hypothetical protein |
| | AAIA71_ 28630 | Hypothetical protein |
| | AAIA71_ 28635 | Hypothetical protein |
| | AAIA71_28640 | Hypothetical protein |
| | AAIA71_28645 | Hypothetical protein |
| | AAIA71_ 28650 | Hypothetical protein |
| | AAIA71_ 28655 | Hypothetical protein |
| | AAIA71_ 28660 | Hypothetical protein |
| | AAIA71_ 28665 | Hypothetical protein |
| | AAIA71_ 28670 | Hypothetical protein |
| | AAIA71_ 28675 | Hypothetical protein |
| | AAIA71_28680 | Hypothetical protein |
| | AAIA71_ 28685 | Hypothetical protein |
| | AAIA71_ 28690 | P-loop NTPase fold protein |
| | AAIA71_ 28695 | IS3 family transposase |
| | AAIA71_ 28700 | Hypothetical protein |
| | AAIA71_ 28705 | Hypothetical protein |
| | AAIA71_28710 | DUF2913 family protein |
| | AAIA71_ 28715 | Hypothetical protein |
| | AAIA71_ 28720 | Hypothetical protein |
| | AAIA71_ 28725 | DNA cytosine methyltransferase |
| | AAIA71_ 28735 | Tolc family outer membrane protein |
| | AAIA71_ 28740 | OmpA family protein |
| | AAIA71_28745 | IS5 family transposase |
| | AAIA71_ 28750 | Hypothetical protein |
| | AAIA71_ 28755 | Hypothetical protein |
| | AAIA71_28760 | ParA family protein |
| pN8T11b | AAIA71_28765 | Rpn family recombination-promoting nuclease/putative transposase |
| | AAIA71_28770 | Hypothetical protein |
| | AAIA71_ 28775 | Hypothetical protein |
| | AAIA71_28780 | TraY domain-containing protein |
| | AAIA71_ 28780 | Type IV conjugative transfer system pilin TraA |
| | AAIA71_ 28785 | Type IV conjugative transfer system protein Tral |

| | |
|---|---|
| AAIA71_ 28790 | Type IV conjugative transfer system protein TraE |
| AAIA71_ 28795 | Type-F conjugative transfer system secretin TraK |
| AAIA71_ 28805 | Trbl/virB10 family protein |
| AAIA71_ 28805 | Type IV conjugative transfer system lipoprotein TraV |
| AAIA71_28815 | Hypothetical protein |
| AAIA71_ 28815 | Type IV secretion system protein TraC |
| TAAIA71_ 28820 | Type-F conjugative transfer system protein TraW |
| AAIA71_ 28830 | Hypothetical protein |
| AAIA71_ 28830 | Conjugal transfer pilus assembly protein TraU |
| AAIA71_ 28840 | Hypothetical protein |
| AAIA71_ 28840 | Type-F conjugative transfer system pilin assembly protein TrbC |
| AAIA71_ 28845 | Type-F conjugative transfer system mating-pair stabilization protein TraN |
| AAIA71_ 28855 | Hypothetical protein |
| AAIA71_ 28860 | Hypothetical protein |
| AAIA71_ 28865 | Hypothetical protein |
| AAIA71_ 28870 | Hypothetical protein |
| AAIA71_ 28875 | Hypothetical protein |
| AAIA71_ 28875 | Type-F conjugative transfer system pilin assembly protein TraF |
| AAIA71_28885 | ATP-binding protein |
| AAIA71_ 28885 | Type-F conjugative transfer system pilin assembly thiol-disulfide isomerase |
| AAIA71_ 28895 | Conjugal transfer protein TraH |
| AAIA71_ 28900 | Conjugal transfer protein TraG N-terminal domain-containing protein |
| AAIA71_ 28905 | Hypothetical protein |
| AAIA71_28910 | Hypothetical protein |
| AAIA71_ 28910 | Type IV conjugative transfer system coupling protein TraD |
| AAIA71_ 28920 | Hypothetical protein |
| AAIA71_ 28925 | Adenosine deaminase |
| AAIA71_ 28930 | Hypothetical protein |
| AAIA71_ 28935 | Hypothetical protein |
| AAIA71_ 28940 | Hypothetical protein |
| AAIA71_ 28945 | Hypothetical protein |
| AAIA71_ 28950 | Hypothetical protein |
| AAIA71_ 28950 | Conjugative transfer relaxase/helicase Tral |
| AAIA71_28960 | Hypothetical protein |
| AAIA71_ 28965 | Lytic trans glycosylases domain-containing protein |
| AAIA71_ 28970 | Type I restriction endonuclease subunit M |
| AAIA71_ 28975 | Hypothetical protein |
| AAIA71_28980 | Hypothetical protein |
| AAIA71_28985 | Hok /Gef family protein |
| AAIA71_ 28990 | DUF2913 family protein |

| | | |
|---|---|---|
| | AAIA71_28995 | Hypothetical protein |
| | AAIA71_ 29000 | Panacea domain-containing protein |
| | AAIA71_ 29005 | Hypothetical protein |
| | AAIA71_ 29010 | ATP-binding protein |
| | AAIA71_ 29015 | Recombinase family protein |
| | AAIA71_ 29020 | HEPN domain-containing protein |
| | AAIA71_ 29025 | Type II toxin-antitoxin system Phd/efM family antitoxin |
| | AAIA71_29030 | Type II toxin-antitoxin system RelE/ParE family toxin |
| | AAIA71_ 29035 | Transposase |
| | AAIA71_29040 | DUF5710 domain-containing protein |
| | AAIA71_ 29045 | Hypothetical protein |
| | AAIA71_29050 | IS91 family transposase |
| | AAIA71_ 29055 | Domain-containing protein |
| | AAIA71_ 28730 | Lg-like domain-containing protein |
| | AAIA71_ 29060 | Type IV conjugative transfer system coupling protein TraD |
| | AAIA71_29065 | Hypothetical protein |
| | AAIA71_29070 | Adenosine deaminase |
| | AAIA71_ _29075 | Hypothetical protein |
| | AAIA71_ 29080 | Conjugative transfer relaxase/helicase Tral |
| | AAIA71_ 29085 | Hypothetical protein |
| | AAIA71_ 29090 | Lytic trans glycosylase domain-containing protein |
| | AAIA71_ 29095 | Type I restriction endonuclease subunit M |
| | AAIA71_ 29100 | Hypothetical protein |
| | AAIA71_ 29105 | Hypothetical protein |
| | AAIA71_ 29110 | Outer membrane beta-barrel protein |
| | AAIA71_ 29115 | Hypothetical protein |
| | AAIA71_ 29120 | DNA, repair protein RadC |
| | AAIA71_ 29125 | Hypothetical protein |
| pN8T11c | AAIA71_ 29130 | Hypothetical protein |
| | AAIA71_29135 | Hypothetical protein |
| | AAIA71_ 29140 | Hypothetical protein |
| | AAIA71_ 29145 | Hypothetical protein |
| | AAIA71_29150 | Hypothetical protein |
| | AAIA71_29155 | FRG domain-containing protein |
| | AAIA71_ 29160 | Hypothetical protein |
| | AAIA71_ 29165 | Hypothetical protein |
| | AAIA71_29170 | Hypothetical protein |
| | AAIA71_ 29175 | DNA-binding protein |
| | AAIA71_ 29180 | Type II toxin-antitoxin system HigB family toxin |
| | AAIA71_ 29185 | CesT family type Ill secretion system chaperone |
| | AAIA71_ 29190 | Hypothetical protein |
| | AAIA71_ 29195 | Hypothetical protein |
| | AAIA71_ _29200 | Hypothetical protein |
| | AAIA71_ 29205 | Hypothetical protein |

| | |
|---|---|
| AAIA71_29210 | Recombinase family protein |
| AAIA71_29215 | Hypothetical protein |
| AAIA71_ 29220 | Type II toxin-antitoxin system RelE/ParE family toxin |
| AAIA71_ 29225 | Type II toxin-antitoxin system Phd/yefM family antitoxin |
| AAIA71_ 29230 | Hypothetical protein |
| AAIA71_ 29235 | IS110 family transposase |
| AAIA71_ 29240 | Hypothetical protein |
| AAIA71_29245 | DUF3265 domain-containing protein |
| AAIA71_ 29250 | Restriction endonucleases |
| AAIA71_29255 | Hypothetical protein |
| AAIA71_ 29260 | Abi family protein |
| AAIA71_ 29265 | Hypothetical protein |
| AAIA71_ 29270 | Hypothetical Protel |
| AAIA71_ 29275 | Hypothetical protein |
| AAIA71_29280 | ParA family protein |
| AAIA71_ 29285 | Rpn family recombination-promoting nuclease/putative transposase |
| AAIA71_ 29290 | Hypothetical protein |
| AAIA71_29295 | TraY domain-containing protein |
| AAIA71_ 29300 | Type IV conjugative transfer system pilin TraA |
| AAIA71_ 29305 | Type IV conjugative transfer system protein Tral |
| AAIA71_ 28790 | Type IV conjugative transfer system protein TraE |
| AAIA71_ 28795 | Type-F conjugative transfer system secretin TraK |
| AAIA71_ 29320 | Trbl virB10 family protein |
| AAIA71_ 29325 | Type IV conjugative transfer system lipoprotein TraV |
| AAIA71_ 29330 | Type IV secretion system protein TraC |
| AAIA71_ 29335 | Type-F conjugative transfer system protein TraW |
| AAIA71_ 29340 | Hypothetical protein |
| AAIA71_ 29345 | Hypothetical protein |
| TAAIA71_ 29350 | Conjugal transfer pilus assembly protein TraU |
| AAIA71_ 29355 | Type-F conjugative transfer system pilin assembly protein TrbC |
| AAIA71_ 29360 | Type-F conjugative transfer system mating-pair stabilization protein TraN |
| AAIA71_ 29365 | Hypothetical protein |
| AAIA71_29370 | Hypothetical protein |
| AAIA71_ 29375 | Hypothetical protein |
| AAIA71_29380 | Hypothetical protein |
| AAIA71_ 29385 | Type-F conjugative transfer system pilin assembly protein TraF |
| AAIA71_ 29390 | Type-F conjugative transfer system pilin assembly thiol-disulfide isomerase TrbB |
| AAIA71_ 29395 | Conjugal transfer protein TraH |
| AAIA71_ 29400 | Conjugal transfer mating-pair stabilization protein TraG |
| AAIA71_ 29405 | Hypothetical protein |
| AAIA71_ 29410 | Hypothetical protein |

| | | |
|---|---|---|
| | AAIA71_ 29415 | Hok/Gef family protein |
| | AAIA71_ 29420 | TonB-dependent hemoglobin receptor |
| | AAIA71_ 29425 | Recombinase family protein |
| | AAIA71_ 29430 | Helix-turn-helix domain-containing protein |
| | AAIA71_ 29435 | PIN domain-containing protein |
| | AAIA71_ 29440 | Hypothetical protein |
| | AAIA71_ 29445 | YegP family protein |
| | AAIA71_29450 | 3'-5' exonuclease |
| | AAIA71_ 29455 | Hypothetical protein |
| | AAIA71_ 29460 | Hypothetical protein |
| | AAIA71_29465 | Type I restriction endonuclease subunit M |
| | AAIA71_ 29470 | Lytic trans glycosylase domain-containing protein |
| | AAIA71_ 29475 | Hypothetical protein |
| | AAIA71_ 29495 | Conjugative transfer relaxase/helicase Tral |
| | AAIA71_29485 | Hypothetical protein |
| | AAIA71_ 29490 | Hypothetical protein |
| | AAIA71_29495 | Hypothetical protein |
| | AAIA71_ 29500 | Adenosine deaminase |
| | AAIA71_ 29505 | Hypothetical protein |
| | AAIA71_ 29510 | Type Iv conjugative transfer system coupling protein TraD |
| | AAIA71_ 29515 | Hypothetical protein |
| | AAIA71_ 29520 | Hypothetical protein |
| pN8T11d | AAIA71_ 29525 | Conjugal transfer mating-pair stabilization protein TraG |
| | AAIA71_ 29530 | Conjugal transfer protein TraH |
| | AAIA71_ 29535 | Type-F conjugative transfer system pilin assembly thiol-disulfide isomerase TrbB |
| | AAIA71_ 29540 | ATP-binding protein |
| | AAIA71_ 29545 | Type-F conjugative transfer system pilin assembly protein |
| | AAIA71_ 29550 | Hypothetical protein |
| | AAIA71_ 29555 | Hypothetical protein |
| | AAIA71_ 29560 | Hypothetical protein |
| | AAIA71_ 29565 | Hypothetical protein |
| | AAIA71_ 29570 | Hypothetical protein |
| | AAIA71_ 29575 | Type-F conjugative transfer system mating-pair stabilization protein |
| | AAIA71_ 29580 | Type-F conjugative transfer system pilin assembly protein TrbC |
| | AAIA71_ 29585 | Hypothetical protein |
| | AAIA71_ 29590 | Conjugal transfer pilus assembly protein TraU |
| | AAIA71_ 29595 | Hypothetical protein |
| | AAIA71_ 29600 | Type-F conjugative transfer system protein Trav |
| | AAIA71_ 29605 | Type IV secretion system protein TraC |
| | AAIA71_ 29610 | Hypothetical protein |
| | AAIA71_ 29615 | Type IV conjugative transfer system lipoprotein Traw |

| | | |
|---|---|---|
| | AAIA71_ 29620 | TrbI/virB 10 family protein |
| | AAIA71_ 29625 | Type-F conjugative transfer system secretin TraK |
| | AAIA71_ 29630 | Type IV conjugative transfer system protein TraE |
| | AAIA71_ 29635 | Type IV conjugative transfer system protein TraI |
| | AAIA71_ 29640 | Type IV conjugative transfer system pilin TraA |
| | AAIA71_ 29645 | TraY domain-containing protein |
| | AAIA71_ 29650 | Helix-turn-helix transcriptional regulator |
| | AAIA71_ 29655 | Hypothetical protein |
| | AAIA71_ 29660 | Ron family recombination-promoting nuclease/putative transposase |
| | AAIA71_ 29665 | ParA family protein |
| | AAIA71_ 29670 | Hypothetical protein |
| | AAIA71_ 29675 | Hypothetical protein |
| | AAIA71_ 29680 | IS91 family transposases |
| | AAIA71_ 29685 | IS1 family transposase |
| | AAIA71_ 29690 | Hypothetical protein |
| | AAIA71_ 29695 | Hypothetical protein |
| | AAIA71_ 29700 | IS91 family transposase |
| | AAIA71_ 29705 | Hydroxyisourate hydrolase |
| | AAIA71_ 29710 | IS5/1S1182 family transposase |
| pN8T11e | AAIA71_ 29715 | A lactococcal endoribonuclease belonging to the type III toxin–antitoxin system |

**Table S11.** Plasmids from other sources similar to those found in N8T11

| Plasmid ref | Results plasmid accession num | Results plasmid name | Shared hashes | Mash Neighbor Distance | Host species | Results plasmid host stain ID | Biosample location |
|---|---|---|---|---|---|---|---|
| pN8T11b | NZ CP018682.2 | QT520 p1 | 263 | 0 | *V. harveyi* | QT520 | China: Chenmai |
| | NZ CP150471.1 | Pvh4 | 255 | 0.0434809 | *V. harveyi* | NH-LM1 | China: Bohai Bay |
| | NZ CP150472.1 | Pvh5 | 255 | 0.0583726 | *V. harveyi* | NH-LM1 | China: Bohai Bay |
| | NZ CP150469.1 | Pvh2 | 236 | 0.0563082 | *V. harveyi* | NH-LM1 | China: Bohai Bay |
| | NZ CP150470.1 | Pvh3 | 156 | 0.0465005 | *V. harveyi* | NH-LM1 | China: Bohai Bay |
| | NZ CP025540.1 | P345-67 | 83 | 0 | *V. harveyi* | 345 | China: Shenzhen |
| | NZ CP018684.2 | QT520 p3 | 82 | 0 | *V. harveyi* | QT520 | China: Chenmai |
| | NZ CP150908.1 | pHLE-202006 | 81 | 0.0450347 | *V. parahaemolyticus* | Vp-HL-202006 | China: Fujian |
| | NZ CP150915.1 | pHLE-202008 | 81 | 0.0450347 | *V. parahaemolyticus* | Vp-HL-202008 | China: Fujian |
| | NZ CP117056.1 | unnamed1 | 68 | 0.0567566 | *V. harveyi* | VH21FL | South Korea: Pohang |
| | NZ CP018683.2 | QT520 p2 | 67 | 0 | *V. harveyi* | QT520 | China: Chenmai |
| pN8T11c | NZ CP018683.2 | QT520 p2 | 250 | 0 | *V. harveyi* | QT520 | China: Chenmai |
| | NZ CP117056.1 | unnamed1 | 241 | 0.0567566 | *V. harveyi* | VH21FL | China: Bohai Bay |
| | NZ CP150470.1 | Pvh3 | 225 | 0.0465005 | *V. harveyi* | NH-LM1 | China: Bohai Bay |
| | NZ CP150908.1 | PHLE-202006 | 205 | 0.0450347 | *V. parahaemolyticus* | Vp-HL-202006 | China: Fujian |
| | NZ CP150915.1 | PHLE-202008 | 205 | 0.0450347 | *V. parahaemolyticus* | Vp-HL-202008 | China: Fujian |
| | NZ CP018684.2 | QT520 p3 | 193 | 0 | *V. harveyi* | QT520 | China: Chenmai |
| | NZ CP150473.1 | Pvh6 | 185 | 0.0455159 | *V. harveyi* | NH-LM1 | China: Bohai Bay |

| | | | | | | | |
|---|---|---|---|---|---|---|---|
| | NZ CP025540.1 | p345-67 | 162 | 0 | *V. harveyi* | 345 | China: Shenzhen |
| | NZ CP150468.1 | Pvh1 | 156 | 0.0615839 | *V. harveyi* | NH-LM1 | China: Bohai Bay |
| | NZ CP150469.1 | Pvh2 | 142 | 0.0563082 | *V. harveyi* | NH-LM1 | China: Bohai Bay |
| | NZ CP150472.1 | Pvh5 | 88 | 0.0583726 | *V. harveyi* | NH-LM1 | China: Bohai Bay |
| | NZ CP150469.1 | Pvh2 | 284 | 0.0563082 | *V. harveyi* | NH-LM1 | China: Bohai Bay |
| | NZ CP150471.1 | Pvh4 | 275 | 0.0434809 | *V. harveyi* | NH-LM1 | China: Bohai Bay |
| | NZ CP150472.1 | Pvh5 | 213 | 0.0583726 | *V. harveyi* | NH-LM1 | China: Bohai Bay |
| | NZ CP018682.2 | QT520 p1 | 191 | 0 | *V. harveyi* | QT520 | China: Chenmai |
| | NZ CP150470.1 | Pvh3 | 136 | 0.0465005 | *V. harveyi* | NH-LM1 | China: Bohai Bay |
| | NZ CP018684.2 | QT520 p3 | 100 | 0 | *V. harveyi* | QT520 | China: Chenmai |
| pN8T11d | NZ CP012741.1 | unnamed | 99 | 0 | *V. vulnificus* | FORC_017 | South Korea: Jeju island |
| | NC 009703.1 | PC4602-2 | 97 | 0.0123533 | *V. vulnificus* | CECT4602 | Spain |
| | NZ CP014638.1 | PR99 | 97 | 0 | *V. vulnificus* | CECT 4999 | Spain |
| | NZ CP044208.1 | PVv180806 | 95 | 0.0123533 | *V. vulnificus* | Vv180806 | China: Guangzhou |
| | NZ CP134786.1 | PVv GCU-01-2 | 89 | 0.0165552 | *V. vulnificus* | GCU-01 | South Korea: Kimpo |
| | NZ CP025540.1 | P345-67 | 86 | 0 | *V. harveyi* | 345 | China: Shenzhen |
| | NZ CP117056.1 | Unnamed1 | 84 | 0.0567566 | *V. harveyi* | VH21FL | South Korea: Pohang |
| | NZ CP018683.2 | QT520 p2 | 79 | 0 | *V. harveyi* | QT520 | China: Chenmai |
| | NZ CP150473.1 | Pvh6 | 71 | 0.0455159 | *V. harveyi* | NH-LM1 | China: Bohai Bay |
| | NZ CP150908.1 | PHLE-202006 | 70 | 0.0450347 | *V. parahaemolyticus* | Vp-HL-202006 | China: Fujian |
| | NZ CP150915.1 | PHLE-202008 | 70 | 0.0450347 | *V. parahaemolyticus* | Vp-HL-202008 | China: Fujian |
